# Genomic and transcriptomic analysis unveils population evolution and development of pesticide resistance in fall armyworm *Spodoptera frugiperda*

**DOI:** 10.1007/s13238-020-00795-7

**Published:** 2020-10-27

**Authors:** Furong Gui, Tianming Lan, Yue Zhao, Wei Guo, Yang Dong, Dongming Fang, Huan Liu, Haimeng Li, Hongli Wang, Ruoshi Hao, Xiaofang Cheng, Yahong Li, Pengcheng Yang, Sunil Kumar Sahu, Yaping Chen, Le Cheng, Shuqi He, Ping Liu, Guangyi Fan, Haorong Lu, Guohai Hu, Wei Dong, Bin Chen, Yuan Jiang, Yongwei Zhang, Hanhong Xu, Fei Lin, Bernard Slippers, Alisa Postma, Matthew Jackson, Birhan Addisie Abate, Kassahun Tesfaye, Aschalew Lemma Demie, Meseret Destaw Bayeleygne, Dawit Tesfaye Degefu, Feng Chen, Paul K. Kuria, Zachary M. Kinyua, Tong-Xian Liu, Huanming Yang, Fangneng Huang, Xin Liu, Jun Sheng, Le Kang

**Affiliations:** 1 State Key Laboratory for Conservation and Utilization of Bioresources in Yunnan, Yunnan Agricultural University, Kunming, 650201 China; 2 State Key Laboratory of Agricultural Genomics, BGI-Shenzhen, Shenzhen, 518083 China; 3 State Key Laboratory of Integrated Management of Pest Insects and Rodents, Institute of Zoology, Chinese Academy of Sciences, Beijing, 100101 China; 4 Beijing Institutes of Life Science, Chinese Academy of Sciences, Beijing, 100101 China; 5 MGI, BGI-Shenzhen, Shenzhen, 518083 China; 6 BGI-Qingdao, BGI-Shenzhen, Qingdao, 266555 China; 7 BGI-Yunnan, No. 389 Haiyuan Road, High-tech Development Zone, Kunming, 650106 China; 8 China National GeneBank, Jinsha Road, Dapeng New District, Shenzhen, 518120 China; 9 Department of Biology, University of Copenhagen, 2100 Copenhagen, Denmark; 10 Guangdong Provincial Key Laboratory of Genome Read and Write, BGI-Shenzhen, Shenzhen, 518120 China; 11 Guangdong Provincial Academician Workstation of BGI Synthetic Genomics, BGI-Shenzhen, Shenzhen, 518120 China; 12 Yunnan Plateau Characteristic Agriculture Industry Research Institute, Kunming, 650201 China; 13 College of Plant Health and Medicine, Qingdao Agricultural University, Qingdao, 266109 China; 14 Department of Entomology, Louisiana State University Agricultural Center, Baton Rouge, LA 70803 USA; 15 CAS Center for Excellence in Biotic Interactions, University of Chinese Academy of Sciences, Beijing, 100101 China; 16 Yunnan Plant Protection and Quarantine Station, Kunming, 650034 China; 17 State Key Laboratory for Conservation and Utilization of Subtropical Agro-Bioresources, South China Agricultural University, Guangzhou, 510642 China; 18 BGI-Americas, One Broadway, 14th Floor, Cambridge, MA 02142 USA; 19 Department of Biochemistry, Genetics and Microbiology, Forestry and Agricultural Biotechnology Institute, University of Pretoria, Pretoria, South Africa; 20 Ethiopian Biotechnology Institute, Addis Ababa, Ethiopia; 21 College of Natural Science, Addis Ababa University, Addis Ababa, Ethiopia; 22 Melkassa Agricultural Research Center, Ethiopian Institute of Agricultural Research, Melkassa, Addis Ababa, Ethiopia; 23 Kenya Agricultural and Livestock Research Organization, P.O. Box 57811, Nairobi, 00800 Kenya

**Keywords:** *Spodoptera frugiperda*, chromosome-level genome, population differentiation, cytochrome p450, pesticides

## Abstract

The fall armyworm (FAW), *Spodoptera frugiperda*, is a destructive pest native to America and has recently become an invasive insect pest in China. Because of its rapid spread and great risks in China, understanding of FAW genetic background and pesticide resistance is urgent and essential to develop effective management strategies. Here, we assembled a chromosome-level genome of a male FAW (SFynMstLFR) and compared re-sequencing results of the populations from America, Africa, and China. Strain identification of 163 individuals collected from America, Africa and China showed that both C and R strains were found in the American populations, while only C strain was found in the Chinese and African populations. Moreover, population genomics analysis showed that populations from Africa and China have close relationship with significantly genetic differentiation from American populations. Taken together, FAWs invaded into China were most likely originated from Africa. Comparative genomics analysis displayed that the cytochrome p450 gene family is extremely expanded to 425 members in FAW, of which 283 genes are specific to FAW. Treatments of Chinese populations with twenty-three pesticides showed the variant patterns of transcriptome profiles, and several detoxification genes such as *AOX*, *UGT* and *GST* specially responded to the pesticides. These findings will be useful in developing effective strategies for management of FAW in China and other invaded areas.

**Electronic supplementary material:**

The online version of this article (10.1007/s13238-020-00795-7) contains supplementary material, which is available to authorized users.

## INTRODUCTION

The fall armyworm (FAW), *Spodoptera frugiperda*, is a devastating and highly migratory insect herbivore native to tropical and sub-tropical areas in America, and was first reported more than 100 years ago in the USA as a damaging pest to maize and other crops (Hinds and Dew, 1915). Currently, FAW invasion has been reported in more than 100 countries worldwide (Fig. S1). FAW larvae may attack more than 350 host plant species belonging to 76 plant families (Montezano et al., 2018), causing serious damage to crops including maize, rice, sugarcane, sorghum, and cotton (Sparks, 1979). The strong migration capacity of FAW adults has led to its recently rapid and wide spread throughout Africa, the Middle East, and several Asian countries including India, Bangladesh, Sri Lanka, Thailand, Yemen, Myanmar and China since their first appearance in west Africa in early 2016 (Goergen et al., 2016; Mallapur et al., 2018; CABI, 2019; Farmer, 2019). In January 2019, FAW was first reported in Yunnan Province of China (Jing et al., 2019), and then rapidly spread to 13 provinces and cities (Yunnan, Guizhou, Sichuan, Chongqing, Guangxi, Guangdong, Hainan, Hunan, Hubei, Fujian, Zhejiang, Jiangxi and Henan) of China within only four months (Wu et al., 2019). With the fast dispersal rates and available suitable habitats in the southwest, central and northern regions of China, the pest had already attacked 1,366 counties (cities and districts) across 24 provinces of China until August 17, 2019 (Xiao et al., 2020). The recent FAW invasion in China has caused tremendous losses of agricultural production in the invaded regions and has become a great threat to the corn production in eastern China, the major regions of corn production in China (Li et al., 2019).

The FAW consists of two morphological identical but genetically distinct strains, the corn strain (C strain) and rice strain (R strain) (Pashley, 1986; Juárez et al., 2014; Nagoshi et al., 2018). The C strain is the major type that widely spreads around the world. Each strain shows specific physiological traits thereby exhibiting strain-specific response to biological and chemical agents (Nagoshi and Meagher, 2004; Meagher and Nagoshi, 2012; Unbehend et al., 2014). The C strain larvae are more tolerant to pesticides methyl parathion, cypermethrin and *Bacillus thuringiensis* (*Bt*) ∂-endotoxins than the R strain (Adamczyk et al., 1997). Therefore, the confirmation of FAW strain type(s) invading China is critical for the effective management of the pest.

FAW management would benefit from detailed and comprehensive genomic analysis. However, the FAW genomes from its cell lines or strains are fragmented without anchoring on chromosomes before the year 2019 (Gouin et al., 2017; Kakumani et al., 2014; Nam et al., 2018, 2019; Nandakumar et al., 2017). With the advancement in genome sequencing and assembly technology, three chromosome-level genome assemblies of FAW have been published or pre-printed (Nam et al., 2019; Zhang et al., 2019, 2020; Xiao et al., 2020). Nevertheless, only two of them are available to the public (Xiao et al., 2020; Zhang et al., 2020). In addition, the genome sizes of the three assemblies varied dramatically from 384 to 486 Mb, even considering the sequencing technologies and potential strain differences. The quality of the genome assemblies is the foundation of population genetic analysis; therefore, more accurate FAW genome assembly and population genomes are urgently needed.

In this study, we comprehensively analyzed the results from *de novo* genome sequences, re-sequencing data of America, Africa, and China populations, as well as transcriptomic profiles after pesticide treatments. We assembled a chromosome-level genome using one male FAW collected from Yunnan, the first place of the pest occurred in China. We also analyzed the strain types of the FAW population that invaded Yunnan, and also discussed the possible origin of the invaded FAW populations in China. In addition, we screened expanded gene families in FAW to determine key genes associated with the function of polyphagia and resistance to pesticides. The results provide essential information for managing FAW invaded in China.

## RESULTS

### Genome assembly, evaluation and annotation

**Figure 1:**
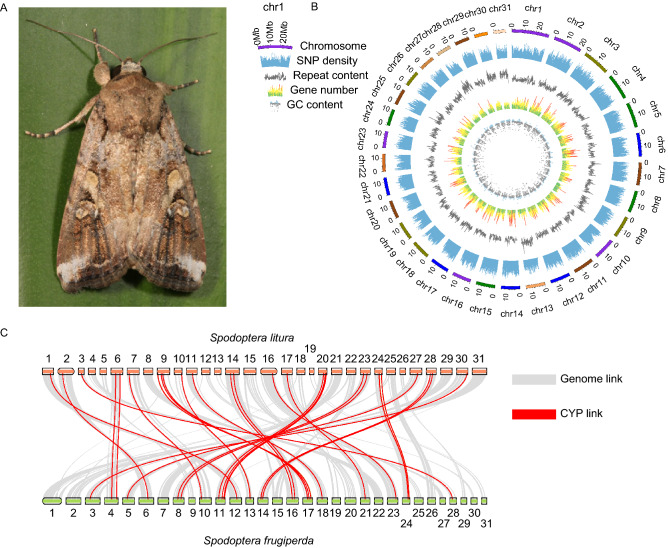
**FAW genomic characterization and synteny with Spodoptera litura.** (A) The male adult of fall armyworm (FAW) collected in Yunnan used for genome assembly. (B) Genomic characterization of FAW genome, it shows the GC content, gene number, repeat content, Single Nucleotide Polymorphism (SNP) density and chromosome from the center to the edge. (C) Synteny between FAW and *Spodoptera litura* genome. The gray lines reveal the genome-wide collinearity, and the red lines reveal the Cytochrome P450 (*CYP*) gene collinearity

A total of 110.93 Gb high-quality single tube long fragment read (stLFR) sequencing data was generated from a male individual from Yunnan Province (Table S1 and Fig. 1A). The size of the primary genome assembled by the stLFR data is 542.42 Mb, and the scaffold N50 is 507.12 kb. Using the High-throughput chromosome conformation capture (Hi-C) technology, the scaffolds were finally concatenated to 31 chromosomes, whereby scaffold N50 is 14.16 Mb. Genomes size assembled in this study fell within the scope of 246 Mb to 809 Mb of the *Lepidopteran* species (Triant et al., 2018). However, the size of the genome reported in the current study is larger than that of the previously assembled FAW genomes (Kakumani et al., 2014; Gouin et al., 2017; Nam et al., 2018, 2019; Nandakumar et al., 2017; Zhang et al., 2019, 2020; Xiao et al., 2020) (Table 1). The extra regions of our FAW genome compared with that published by Xiao (Xiao et al., 2020) are evenly distributed on 31 chromosomes (Fig. S2). We mapped the raw data (raw reads used for assembly of our genome and Xiao’s genome) to those regions, and the mapping rates are more than 30%, suggesting the validity of the extra regions. Besides, 1,494 genes were identified in the extra regions, of which 1,421 genes were expressed gene (average FPKM value of control > 0) (Fig. S3) and 512 genes were differential expression genes (DEGs) in 23 pesticide treatments (Fig. S4), indicating the assembled extra regions might be genuine and functional.

**Table 1:** **Comparison of SFynMstLFR and other published** ***Spodoptera frugiperda* genome assemblies.**

Assembly	ASM221328v1	SF_CORN_1	Sf-RVN WGS	ASM75363v2	SFynMstLFR
Bioproject	PRJNA380964	PRJEB13110	PRJNA344686	PRJNA257248	–
Assembly approach	Canu	–	CLC NGS Cell	SOAPdenovo	supernovo
Sequencing platform	PacBio	–	Illumina-HiSeq 2500	Illumina/454/SOLiD	MGISEQ-2000
Number of contigs	2,844	50,014	89,958	119,777	29,584
Contig N50 (bp)	516,067	21,390	10,170	6,276	91,970
Number of scaffolds	2,396	41,562	66,318	37,235	31 + 21809
Scaffold N50 (bp)	601,127	52,317	12,379	53,779	14,162,803
Total gap length (bp)	890,870	11,379,136	2,171,005	27,684,486	37,953,553
Total bases (bp)	514,225,903	436,269,483	391,647,540	358,047,567	543,659,128
Ungapped bases (bp)	513,335,033	424,890,347	389,476,535	330,363,081	505,705,575

The GC content of FAW genome is 36.52% (Fig. 1B) which fell within the scope of 31.6% to 37.7% of its closely related *Lepidopteran* species, *Bicyclus anynana* (Nowell et al., 2017). The genome covers 95% for complete BUSCO genes with duplicated BUSCO components of 9.8% (Table S2). The quality of these data was largely improved, because of more information available from other published FAW genomes (PRJNA380964, PRJNA257248, PRJEB13110, and PRJNA344686) (Table 1), including the recently published FAW assembled using the Pacbio RSII technology (Xiao et al., 2020). The sequencing data generated from libraries for Hi-C, WGS, RNA-seq were also mapped to the assembled genome, and all the mapping rates and sequencing coverage were above 90% (Tables S3 and S4), indicated that most of the sequencing data can be mapped back to the genome, and the K-mer analysis showed that the inferred genome size were various, indicating the possibly big differences on genome size between different individuals (Fig. S5 and Table S5). Moreover, the FAW expressed sequence tags (EST) and transcripts from NCBI were perfectly mapped to the genome with > 90% and > 80% mapping ratios, respectively (Table S6).

Combining *de novo* and homology-based search, we identified 153 Mb repeat elements, accounting for 28.24% of the FAW genome (Fig. 1B). A total of 22,201 genes were annotated using the repeat-masked genome based on *de novo*, RNA-seq and homology-based methods (Tables S7 and S8). The gene set covered 94.2% of complete BUSCO genes, which presented a more complete gene set than previously published FAW genome (Table S9). Totally, 93.48% of the identified genes in the gene set were identified as functional genes (Table S8). The FAW genome established in this study revealed a high sequence coverage and identity with its closely related species, *Spodoptera litura* (Fig. 1C), another important insect pest on vegetable and horticultural plants.

### Comparative genomics revealed the possible genetic basis of pesticides resistance

**Figure 2:**
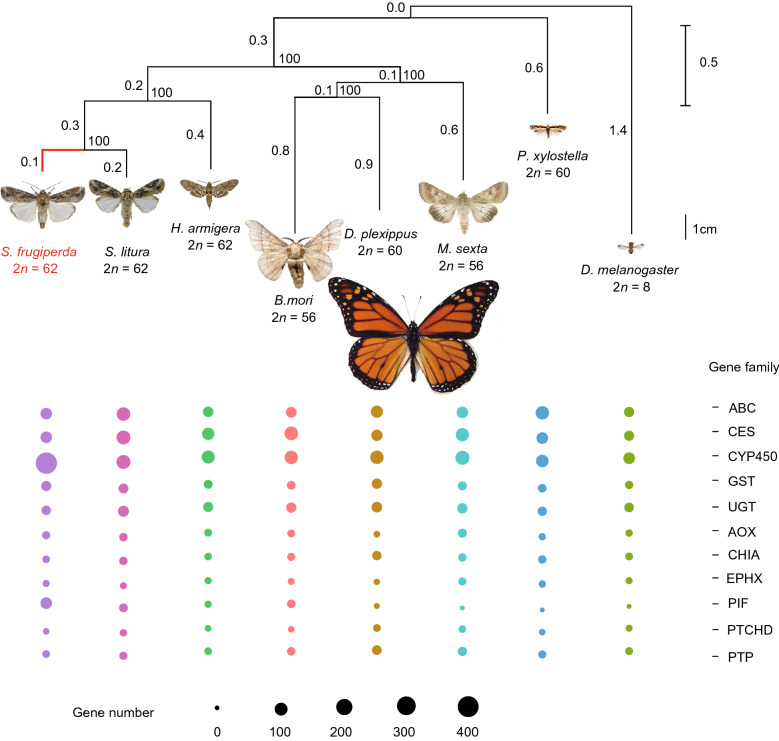
**Phylogenetic relationships among seven lepidopteran and D. melanogaster genomes.** Below: the gene family number of ATP-binding cassette (*ABC*), Carboxylesterase (*CES*), Cytochrome P450 (*CYP*), Glutathione S-transferase (*GST*), UDP-glucuronosyl transferase (*UGT*), Aldehyde Oxidase (*AOX*), Chitinase Acidic (*CHIA*), Epoxide Hydrolase (*EPHX*), PIF1 5′-to-3′ DNA helicase (*PIF*), Patched Domain Containing (*PTCHD*) and protein tyrosine phosphatase (*PTP*) genes

Based on the 4,056,205 bp fourfold degenerate synonymous site (4DTv) sequences across the genomes of FAW and other seven *Lepidopteran* species, the phylogenetic analysis showed that FAW is closely related with *S*. *litura* (Fig. 2). Branch-site likelihood ratio test was performed to identify positively selected genes (PSGs) based on 20,051 single-copy orthologous genes of FAW and *Bombyx mori* abstracted by syntenic alignment, and a total of 363 PSGs were identified, which were enriched in reverse transcriptase domain (IPR000477), endonuclease/exonuclease/phosphatase (IPR005135), zinc finger, CCHC-type (IPR001878), fructose-2,6-bisphosphatase (IPR003094), etc. (Table S10). Of them, *ABCC4*, *UGT* and *ChuaMOX* genes were related to detoxification function.

**Figure 3:**
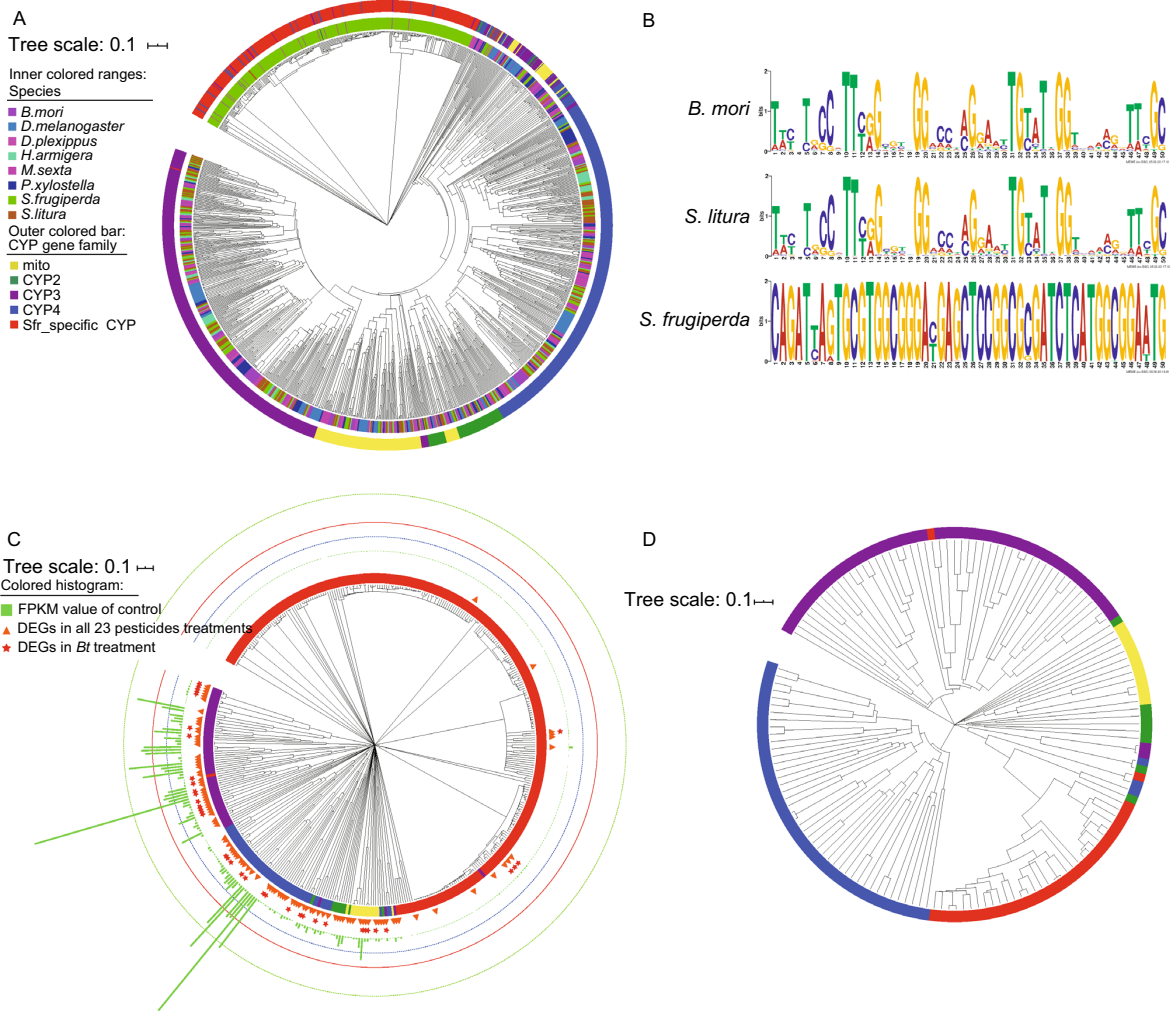
**The gene tree of Cytochrome P450 gene family.** (A) 1,256 Cytochrome P450 (CYP) genes in 8 species, the label color shows different species, the bar color shows different CYP gene sub-families. (B) The seq-logo plot of the motif and 10 bp flanking sequences of CYP genes in 3 species. (C) The 425 CYP genes of FAW, the bar color shows different CYP gene sub-families, 107 differential expression genes (DGEs) between all treatments and control are shown as orange triangle, 37 DGEs between *Bt* treatment and control are shown as red star, and the histogram means CYP gene FPKM value of control. (D) The 152 CYP genes filtered by CD-HIT, the bar color shows different CYP gene sub-families

Several insect gene families are supposed to be associated with detoxification function, including ATP-binding cassette (*ABC*) (Koenig et al., 2015), Carboxyl esterase (*CES*) (Satoh and Hosokawa, 2009), Cytochrome P450 (*CYP*) (Zhu et al., 2013), Glutathione S-transferase (*GST*) (Hayes and Pulford, 2008), UDP-glucuronosyl transferase (*UGT*) (Bock, 2016), Aldehyde Oxidase (*AOX*) (Chang et al., 2010), Chitinase Acidic (*CHIA*) (Downing et al., 2000), Epoxide Hydrolase (*EPHX*) (Iarmarcovai et al., 2008), PIF1 5′-to-3′ DNA helicase (*PIF*) (Erlandson 2009), Patched Domain Containing (*PTCHD*) and Protein Tyrosine Phosphatase (*PTP*) (Herraiz et al., 2006) gene families. Here, we scanned all of these genes or gene families to detect expanded detoxification-related genes (Fig. 2 and Table S11). A total of 1,256 *CYP* genes were detected from these eight insect species, and 425 *CYP* genes were identified in the FAW genome, indicating that they are extremely expanded in FAW than the other seven *Lepidopteran* species. The significant expansion trend is similar with the published FAW genome (Xiao et al., 2020). Besides, the expanded *CYP* genes from FAW shows quite short-time divergence, and 283 genes are specific to FAW (Fig. 3A, 3C and Table S12). According to the DNA sequence identity, the 425 *CYP* genes of FAW were merged into 152 gene clusters (Fig. 3D).

Furthermore, the MEME software verified the accuracy of the *CYP* gene family for the FAW-specific *CYP* gene set based on the control *CYP* genes from *B*. *mori* and *S*. *litura*. The conservation of motif and 10 bp flanking sequences is shown as a seq-logo plot (Fig. 3B). From the highlighted seq-logo plot, the conservation of motifs in the three species (FAW, *B*. *mori*, and *S*. *litura*) displayed a striking variant. The motif sequence in FAW is more conserved than in the other two species.

### Whole genome resequencing

#### *Variation discovery*

**Figure 4:**
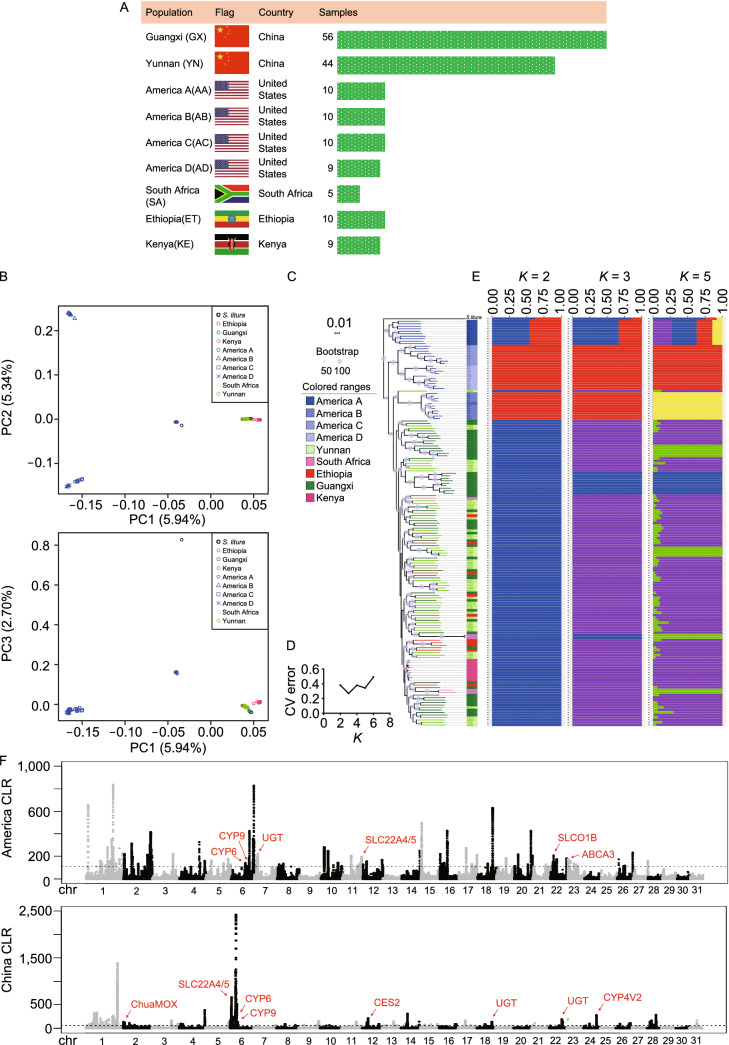
**Phylogeny and population structure of FAW.** (A) Sampling locations of six geographical populations. (Ethiopia: red, Kenya: light red, America: blue, South Africa: pink, Guangxi: green and Yunnan: light green) (B) PCA plots of the first three components of major FAW accessions using whole-genome SNP data. (C) Maximum likelihood phylogenetic tree of FAW population (from what data). Fabricius (*Spodoptera litura*) was used as an outgroup. (D) Cross-validation (CV) error of ADMIXTURE. (E) Population structure of major FAW categories estimated by ADMIXTURE. Each color represents one ancestral population. Each accession is represented by a bar, and the length of each colored segment in the bar represents the proportion contributed by that ancestral population. (F) CLR scores calculated by SweeD across the genome in both American D and Yunnan accessions. The dashed lines mark the regions at the top 0.5%. The red arrow indicates the detoxification candidate gene regions

To assess the genetic diversity among FAW accessions, 163 individuals from America (*n* = 39), Africa (*n* = 24) and China (*n* = 100) (Table S20) were mapped to the FAW assembly (Fig. 4A). The average mapping rate and sequencing coverage were 95.60% ± 3.34% and 90.41% ± 2.38%, respectively (Table S4). After filtration (See “METHODS”), we identified 45,569,102 single nucleotide polymorphism (SNPs) and 11,391,362 short genomic insertions and deletions (InDels) (less than 40 bp) with a minor allele frequency (MAF) > 0.01. About 71.71% of the SNPs are located in the intergenic regions, whereas 3.99% of them are in the coding sequences. The non-synonymous-to-synonymous substitution ratio for the SNPs in the coding regions is 0.242, indicating high quality of the SNP call sets. Moreover, 72.94% of the InDels are located in the intergenic regions, whereas 0.38% of them in the coding regions. About 68.77% of the InDels in the coding regions were estimated to cause frame shift mutations. The SNP density is shown in Figs. 1B and S6.

#### *African origin of FAW populations in China*

The core set of SNPs were used to analyze the phylogeny and population structure of FAW. Principal component analysis (PCA) showed substantial genetic diversity among the six FAW populations, PC1, PC2 and PC3 revealing a total genetic variance of 5.49%, 5.34% and 2.70%, respectively (Fig. 4B). PC1 separated American FAW from other five accessions, PC2 evidently separated the four American accessions and PC3 separated AA accessions from the rest. American populations showed relatively large genetic divergence within populations, compared to Chinese and African populations.

Maximum likelihood (ML) phylogenetic analysis revealed distinct monophyletic clades for Ethiopia (ET), Kenya (KE), America (A), South Africa (SA), Guangxi (GX), and Yunnan (YN) FAW populations (Fig. 4C). This ML-tree further illustrated that the American group could be marginally divided into subgroups AA and AB-AC-AD (Fig. 4C). Chinese (YN, GX) and African (ET, KE, SA) clades displayed close phylogenetic relationship (Fig. 4C). Structure analysis showed that populations in China and Africa comprise accordant components (Fig. 4D and 4E, K = 5). Varying the number of *K* (presumed ancestral populations) revealed genetic distinctions between American and other accessions. When *K* = 2, we observed two separate clusters: American (A) and other accessions, and when *K* = 3, three separate clusters were exhibited: AA, AB-AC-AD and other accessions. Similar patterns are also supported by the PCA and ML-tree analyses (Fig. 4B and 4C), suggesting that Chinese accessions (YN and GX) shared much closer genetic relationships with African accessions (SA, ET, KE) than that of America (AA, AB, AC, AD). Taken together, FAW populations in China (YN, GX) migrated directly from Africa, because the genetic structure of these populations is highly mixed. Besides, we estimated admixture graphs of different geographically defined groups using TreeMix. Extensive gene flow (45.17%) were found from AB to AC when m = 1 (Fig. S7). Additional gene flow from KE to ET (14.66%) and YN (15.17%) were also found when setting the parameter m = 2 and m = 3, respectively. These findings proved that American accessions are deep divergent from other accessions, further supporting that Chinese accessions was derived from Africa, rather than directly from America.

#### *All the samples were identified as C strain in FAW populations invading China*

In our study, we identified 240 samples by using TpiE4-183, including 163 resequencing samples, 72 RNA-seq samples used for pesticide resistance analysis, one sample used for genome assembly survey, one sample used for stLFR genome assembly, one sample used for Hi-C sequencing and two RNA-seq samples used for gene annotation (Tables S1 and S20). Interestingly, all 100 samples from China and 24 samples from Africa were identified as C strain, while both C strain and R strain were found in the American samples (Fig. S8). At present, only C strain was found in the Chinese and African populations, indicating all these samples have similar genomic backgrounds.

#### *Differentiation of FAW demographic histories*

To reveal the demographic and geophylogenic history of FAW, a pairwise sequentially Markovian coalescent (PSMC) model was used to analyze SNP data from FAW populations of America (*n* = 10, three from AA, three from AB, two from AC, and two from AD), Africa (*n* = 10, four from ET, four from KE and two from SA) and China (*n* = 10, five from YN and five from GX). The results were scaled to the real time by assuming a generation time of 0.25 year and a neutral mutation rate of 2.9 × 10^−9^ per year (See “METHODS”). From the one Mya (million years ago), the effective population size of each subgroup was found to be less than 800,000 (Fig. S9). Subsequently, African populations (except for KE), all Chinese populations and AB population from America exhibited a mild *Ne* expansion in 100–400 Kya (thousand years ago) (*Ne* ≈ 500,000–1,000,000), which levels off during 10–100 Kya (*Ne* ≈ 750,000–1,000,000). However, American accessions (except for AB), as well as the KE population from Africa, exhibited a rapid *Ne* expansion during 9–200 Kya (*Ne* from 500,000 to even 16,000,000). Subsequently in 100 Kya, Chinese accessions, as well as ET, SA and AB populations had a rapid *Ne* contraction (*Ne* ≈ 100,000). At 7–10 Kya, American accessions (AA, AC and AD) had a rapid *Ne* contraction as well (*Ne* ≈ 50,000). Overall, we found extremely similar demographic history within Chinese FAW populations, which displayed more similar demographic distribution patterns with most African populations than with American populations.

#### Genetic diversity, population differentiation and natural selections

Across the FAW genome, the global nucleotide diversity (*π*) is well correlated to the global SNP and InDel density (Fig. S6). Subsequent analysis revealed that America accessions were highly polymorphic with AB (*π* = 1.02 × 10^−3^) being less polymorphic than AA (*π* = 2.59 × 10^−3^). AC had a nucleotide diversity of 1.95 × 10^−3^, whereas that of AD was 1.74 × 10^−3^. KE accession exhibited the highest nucleotide diversity of 3.51 × 10^−3^ (Fig. S10 and Table S13).

*F*_ST_ (Weir and Cockerham’s estimator) analysis indicated that AB branch had the greatest genetic divergence compared with the other eight accessions (Table S14), consistent with the result of nucleotide diversity analysis (Fig. S10 and Table S13). AD branch displayed great genetic divergence among SA, ET, KE, YN and GX accessions (Table S14) compared to American accessions. Thus, Chinese FAW populations were evolutionally closer to African FAW than American FAW, further suggesting that Chinese FAW originated from Africa.

We also performed to investigate potential selective signals in the genomes of each FAW group by identifying regions (phromosome length/1000) that scored top 0.5% in the model. The selective sweep signals and harbored gene number are shown in Table S15. As the different management strategies between America and China, which *Bt* crop has been used in America for a long history, whereas chemical pesticides have been largely applied in China, American accession (AA, AB, AC and AD) and Chinese accession (YN and GX) were selected to detect the differences. In American accession, *CYP6*, *CYP9*, *UGT*, *SLC22A4*/*5*, *SLCO1B* and *ABCA3* genes were under positive selection. In Yunnan accession, *ChuaMOX*, *SLC22A4*/*5*, *CYP6*, *CYP9*, *CES2*, *UGT* and *CYP4V2* genes were detected to have signals of selective sweep (Fig. 4F).

### Transcriptomic responses of FAW to pesticides exposure

**Figure 5:**
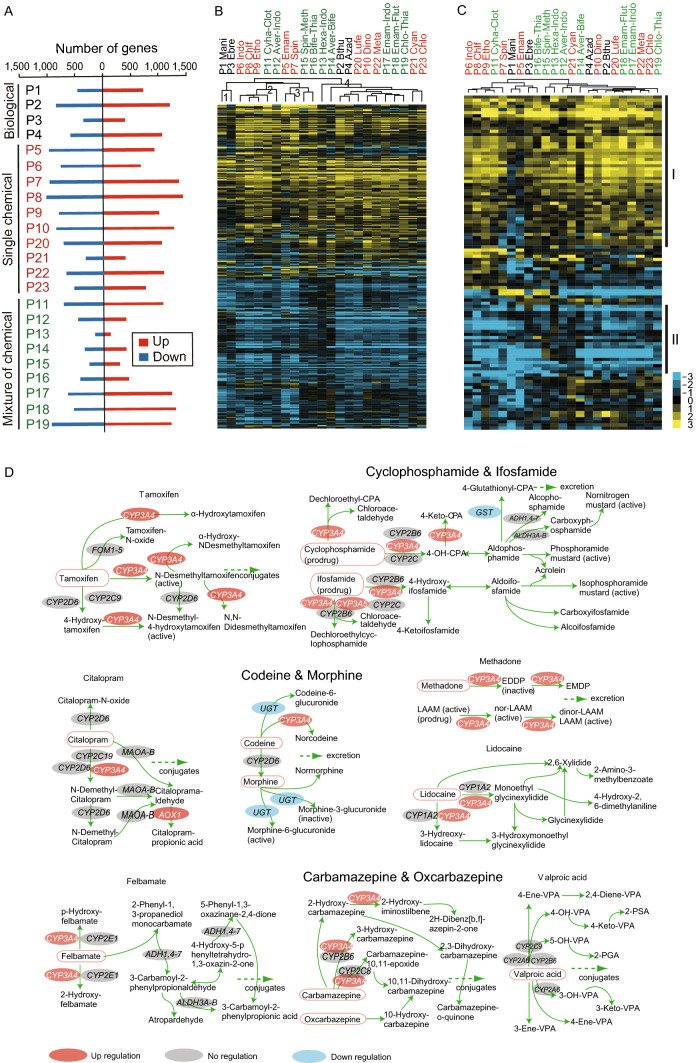
**Gene expression profiling after twenty-three pesticides exposure.** (A) Number of differential expression genes (DEGs) after each pesticide treatment. (B) Pesticides clusters using the combined 7991 DEGs. (C) The heatmap of 107 DGEs of FAW cytochrome P450 genes. X axial shows the different treatment (P1–P23), the color shows the fold change of DGEs, yellow means up regulation, blue means down regulation, black means the gene is not DEG in one/some treatment(s). (D) DGEs of *Bt* involved in hsa00982 pathway. The red means up-regulated DEGs, the blue means down-regulated DEGs

To investigate the transcriptomic profiles of FAW to pesticides, twenty-three pesticides including four biological, ten single and nine mixed chemical pesticides were applied to the third-instar larvae of FAW (Fig. 5 and Table S16). The FAW displayed differential responses to different pesticides with regard to the transcriptional expressions (Fig. 5A). Among the biological pesticides, *Bacillus thuringiensis* (P2) induced the most dramatically transcriptional expression with 1,231 up-regulated and 824 down-regulated genes. In the single and mixed chemical pesticides, eleven pesticides treatments induced the differential expressions of more than 1,500 genes. Then we obtained a union set with 7,991 differential expression genes (DEGs) by merging the DEGs after 23 pesticides treatment. Further clustering of the 7,991 DEGs categorized the 23 pesticides-treated expression profiles into four clusters (Fig. 5B). The four pesticide clusters represent differentially functional groups that effective (Clusters 3 and 4) or ineffective (Clusters 1 and 2) to FAW pest control. Several most effective chemical pesticides in FAW management, such as emamectin benzoate, spinetoram, chlorantraniliprole and cyantraniliprole are concentrated in Clusters 3 and 4, irrespective of single or mixing with other chemicals. Ryanodine receptor-targeted pesticides, such as flutolanil (P18), thiamethoxam (P19), cyantraniliprole (P21) and chlorantraniliprole (P23) were clustered together in Cluster 4. Emamectin benzoate-containing mixture pesticides were clustered with ryanodine receptor-targeted pesticides in Cluster 4. Different with other two biological pesticides, *Bacillus thuringiensis* and azadirachtin (P2 and P4) were also clustered in Cluster 4 (Fig. 5B).

The 107 differentially expressed P450 genes were further clustered (Fig. 5C). Cluster I represented 48 up-regulated genes, including 2 *CYP*2, 26 *CYP*3, 5 *CYP*4, 6 mitochondrial *CYP*s and 9 FAW-specific *CYP*s after 23 pesticides treatment (Fig. 5C). Cluster II represented 22 down-regulated genes, including 3 *CYP*3, 13 *CYP*4, 2 mitochondrial *CYP*s and 4 FAW-specific *CYP*s after 23 pesticides treatment (Fig. 5C). Further KEGG enrichment analysis showed that cutin, suberine and wax biosynthesis (map00073) pathways were mutually up-regulated after nineteen pesticides exposure (Table S17). Meanwhile, many sugar metabolism pathways, such as fructose and mannose metabolism (map00051), amino sugar and nucleotide sugar metabolism (map00520), galactose metabolism (map00052) and pentose and glucuronate interconversions (map00040) pathways were mutually down-regulated after variant pesticides treatment (Table S17).

In addition, we focused on some detoxification gene families such as *CYP*, *UGT*, *GST*, *ABC* and *CES* to identify the potential pesticides resistance genes:

#### CYP gene

Under the selection of pesticide, many insects have evolved to produce new *CYPs*, or *CYP* gene family expands, like *Plutella xylostella* (You et al., 2013), *Aphis glycines* (Wenger et al., 2017) and *Bemisia tabaci* (Chen et al., 2016) and so on. New *CYP* genes or largely expanded *CYP* gene family have potential roles in detoxification and/or pesticide resistance.

In this study, a remarkable change in the expression of the *CYP* genes was observed when FAW individuals were treated with the pesticides. Indeed, many *CYP* genes (107/425) were differently expressed as shown in Fig. 3C and 3D. Fold change values between treatments and controls in the 107 DEGs are shown in Table S18. There were 2,055 DEGs between *Bt*-treated larvae and controls, which contained 37 *CYP* genes including *CYP*4, *CYP*6, *CYP*9, *CYP*12 and *CYP*49 (Figs. 3C and S11). *CYP*49 and most of *CYP*4 showed down regulation, while *CYP*6, *CYP*9 and *CYP*12 exhibited up regulation, which related to pesticide resistance even the cross resistance in multiple pesticides. *Bt* could be an effective pesticide target at many gene like *CYP*. Moreover, the expression of DEGs was significantly elevated in drug metabolism—cytochrome P450 (hsa00982, Fig. 5D) and metabolism of xenobiotics by cytochrome P450 (hsa00980).

#### UGT gene

Glucuronidation is a major conjugation reaction catalyzed by *UGT* family, which is associated with detoxification process reported in many insect genomes, such as *P*. *xylostella* (You et al., 2013) and *B*. *tabaci* (Chen et al., 2016). Transcriptome analysis indicated that all 49 *UGT* genes were detected as expressed genes (FPKM > 0 in any repetition) (Fig. S12), of which 22 genes were DEGs between 23 treatments and control. Notably, there were 9 down-regulated DEGs between chlorfenapyr treatment and control as well as 8 down-regulated DEGs between spinetoram water treatment and control. Glucuronic acid could combine with many harmful substances in insect liver, aiding in the detoxification process, indicating that the two types of pesticides may target *UGT* gene effectively.

#### GST gene

*GST* catalyzes the binding of nucleophilic glutathione to various electron-friendly exogenous chemicals, critical in toxicology. Isomers of *GST* display unusual broad-spectrum catalysis as well as the ability to sequester non-substrate drugs and hormones. Twenty-seven out of 29 *GST* genes were identified as expressed genes (Fig. S13), and 14 genes were DEGs between 23 treatments and control. There were 9 down-regulated DEGs between spinetoram water treatment and control, as well as 6 down-regulated DEGs between chlorfenapyr treatment and control. As a critical role of *GST* gene in toxicology, these two types of pesticides may target at *GST* gene effectively.

#### ABC gene

Research shows that transporters, particularly subfamilies B and C, both of which are involved in detoxification and multidrug resistance, are up-regulated in the gut when larvae feed on plants (Koenig et al., 2015). Besides, *ABC* transporters are targets of *Bacillus thuringiensis* insecticidal toxins (Tay et al., 2015). In this study, 57 out of 58 *ABC* gene families (*ABCA3* and *ABCC4*) were expressed genes (Fig. S14), and 21 genes were DEGs between 23 treatments and control. There were seven down-regulated DEGs between Emamectin*Flutolanil microemulsion treatment and control, implying the *ABC* gene family may be a potential effective target for combination of Emamectin and Flutolanil.

#### CES gene

The *CES* genes encode members of the large carboxylesterase family, which are also involved in detoxification or metabolic activation of various drugs, pesticides, environmental toxins and carcinogens (Satoh and Hosokawa, 2009). In this study, all 30 *CES* genes (*CES1* and *CES2*) were detected as expressed genes (Fig. S15), and of which, 16 genes were DEGs between 23 treatments and control. There were eight down-regulated DEGs between *Metarhizium anisopliae* treatment and control as well as between chlorantraniliprole treatment and control, indicating that the *CES* gene might be a potentially effective target for *Metarhizium anisopliae* and chlorantraniliprole.

#### Other genes

Apart from the above genes (families), there were other DEGs under varied treatments. *SLC22A3*, *SLC22A4* and *SLC22A5* participated in poly-specific transportation of organic cations in the many organs, critical for elimination of many endogenous small organic cations as well as a wide array of drugs and environmental toxins. There were 51 expressed solute carrier family 22 (*SLC22A*) gene member (Fig. S16). Interestingly, there was at least one DEG between each treatment and control, and the total DEG number was thirty-one. There were 21 DEGs between spinetoram water treatment and control, and 16 DEGs between chlorantraniliprole*thiamethoxam treatment and control.

All 60 *ChuaMOX* genes were detected as expressed genes (Fig. S17), and 41 genes were DEGs between all 23 treatments and control. Enzyme encoded by *ChuaMOX* is similar to one produced in sacs of millipede *Chamberlinius hualienensis*. In millipedes, the sacs rupture during defensive behavior, producing benzoyl cyanide—a protective chemical. There were over 15 *ChuaMOX* DEGs in seven treatments (one biological, four single and two mixed chemical pesticides). Besides, *SULT1E1*, *NNT*, *PTPMT1* and *JHEH* genes showed differential expression in different treatments.

Azadirachtin is a chemical compound in limonoid group in seeds as a secondary metabolite. There were 1,657 DEGs between azadirachtin treatment and control, which significantly enriched in detoxification (hsa04146), drug metabolism—cytochrome P450 (hsa00982, Fig. 5D) and metabolism of xenobiotics by cytochrome P450 (hsa00980) about 34 KEGG pathways (Table S19). There were 15 DEGs (Fig. S18) involved in hsa00982 and hsa00980, indicating that similar to *Bt* toxin, azadirachtin pesticide regulates expression of detox gene as well.

*Bt*, *Empedobacter brevis* and azadirachtin act on the digestive system of insects. There were 199 DEGs (Fig. S19) overlapped among them, including *PTPMT1* and *JHEH*, *CES1*, *ChuaMOX*, *SLC22A4* and *SLC22A5* genes associated with detoxification were also among the overlapped DEGs.

## DISCUSSION

Taking the advantages of stLFR and Hi-C technologies with long fragment reads, super high coverage and reads quality, we *de novo* assembled a high-quality FAW genome, which is so far the largest FAW genome assembly with a size of 542.42 Mb (Nandakumar et al., 2017; Zhang et al., 2019, 2020; Xiao et al., 2020). The genome of FAW was greatly different (Kakumani et al., 2014; Gouin et al., 2017; Nam et al., 2018, 2019; Nandakumar et al., 2017; Zhang et al., 2019, 2020; Xiao et al., 2020) (Table 1), which led to the different genome size and unmapped reads among different individuals. However, the FAW genome assembled in the current study is a high-quality genome in most of the key parameters, including the BUSCO evaluation, WGS mapping rate and RNA mapping rate. The quality improvement of the genome may facilitate further biological studies of FAW and help to develop effective strategies for management of this severely invasive pest worldwide. And the individual difference among FAW will be further studied by a pan-genome in the future.

In this study, we identified extremely expanded *CYP* gene family with 425 members (clustered into 152 clusters) compared with those in other assembled FAW genomes or closely related insect species. And the 169 *CYP* genes were identified in the latest research (Xiao et al., 2020), which is close with the *CYP* cluster number in this study. As the *CYP* gene family has the function of intensified detoxification (Cheng et al., 2017), the expansion of *CYP* gene may be associated with the highly polyphagous feeding behaviors of FAW, and its capability to inactivate or degrade xenobiotics to harmless chemicals. Moreover, FAW is frequently controlled by *Bt* crops in America, where the insect pest is originally from, and may have developed multiple resistance to not only pesticides, but biological agents and transgenic crops as well (Morillo and Notz, 2001; Santos-Amaya et al., 2016). The expansion event of *CYP* genes occurred in a short time, implying a rapid adaptation after the application of pesticides. On the other hand, the *CYP* genes annotated as known clan nearly all be DEGs, while the FAW-specific *CYP* genes can hardly be DEGs, which may due to the recent birth of these genes so there is no mature or complete function. However, the resistance caused by the expansion and evolution of *CYP* could hinder the control of FAW and may become the barrier against the usage of *Bt* crops over time. The *CYP* gene family might be valuable genetic targets in developing effective pesticides for FAW management. The results indicated that FAW is highly polyphagous and may be more pesticide-resistant than the other seven species in this study. The identification and mechanism of detoxification of FAW-specific *CYP* requires further investigation.

Resequencing genomic data of FAW from America presented a high genetic diversity, which was consistent with the American origin of FAW (Goergen et al., 2016; Mallapur et al., 2018; Jing et al., 2019). However, the genetic relationship between American populations and African and Chinese group is much far than that between African and Chinese populations. Instead, the extensive allele admixture between the FAW from Africa and China further support that FAW in China was highly likely from African populations, but not from America directly. Among four American populations, AA, AB and AC/AD displayed dramatic discrimination, which is probably partly due to strain differences, because we identified different predominantly strains in different populations (Fig. S8C). In addition, the identification of FAW subtypes in China using *Tpi* gene showed only the C strain, demonstrating an overwhelming proportion in the Chinese FAW population, which was in consistent with the result from Nagoshi et al. (2020). *Tpi* gene was frequently used for subtype identification, but with limitations therefore more markers should be developed in the future to make more accurate determination. The strain identification of the Chinese FAW populations is extremely important for making effective strategies to manage the pest in the country.

One of the main factors making pest control difficult is the evolution of pesticide resistance. Resistance in pest populations usually evolves within 2 to 20 years after application of new types of pesticides such as carbamates, organophosphates, pyrethroids, and *Bt* as a microbial or transgene pesticide (Daly et al., 1998; Weston et al., 2013). The study evaluated the differential expression of genes following exposure to 23 pesticides, which would provide theoretical evidence for appropriate selection and application of pesticides. Most pesticides exhibited similar KEGG pathway enrichments, particularly detoxification (hsa04146), drug metabolism—cytochrome P450 (hsa00982) and metabolism of xenobiotics by cytochrome P450 (hsa00980). This implies that different detoxification genes were up-regulated to resist variant pesticides in FAW. Nonetheless, we conclude that *CYP* plays a pivotal role in resisting pesticides. Thus, our findings on DEGs, particularly *CYP* genes, provide a strong understanding on general resistance to pest-control interventions.

In summary, we assembled a complete chromosome-level genome of a male FAW from Yunnan Province, China. A total of 22,201 genes were predicted in this genome. Cytochrome P450 gene family in FAW was found extremely expanded, which was closely related to detoxification and tolerance to pesticides. Transcriptome analysis of 23 pesticide treatments revealed several candidate target genes. Besides, resequencing analysis of nine populations from six geographical locations in China, Africa and America demonstrated that the FAW in China was likely from Africa. Together, these findings are essential for developing effective strategies to manage the invasive FAW in China and other countries.

## Materials and methods

### Samples and treatments

A total of 240 FAW samples were collected in this study, including 5 samples used for genome assembly, 72 samples used for RNA-seq analysis with 23 pesticide treatments and one control (3 biological sample duplicates (Table S16)), and 163 samples used for population resequencing. These samples collected from three continents, which were America (*n* = 39, A); Africa (*n* = 24; including South Africa (*n* = 5, SA), Ethiopia (*n* = 10, ET), Kenya (*n* = 9, KE)); and Asia (China, *n* = 177; including Guangxi (*n* = 56, GX), Yunnan (*n* = 118, YN) and Guangdong (*n* = 3)) (Table S20). American population was divided into four sub-populations: Population A was collected from grasses (braidleaf signalgrass, *Urochloa platyphylla*) in Franklin Parish, Louisiana on August 1st, 2019. Field-collected larvae were reared in a meridic diet (Ward’s Stonefly Heliothis diet, Rochester, NY) in the lab until adult emergence. Population B was collected from rice in a greenhouse in East Baton Rouge Parish, Louisiana in 2017, and has been reared in the meridic diet mentioned above since it was collected from the greenhouse. This population survived well on rice plants in greenhouse trials. Population C was collected from corn field in Franklin Parish, Louisiana in 2016. It has been reared in the meridic diet mentioned above since it was collected from the field, and survived well on corn plants. Population D was a hybrid of three populations, which were collected from corn plants in Collier County, Florida in 2011, Tift County, Georgia in 2013 and Franklin Parish, Louisiana in 2016, respectively. These field-collected larvae have been reared in the meridic diet mentioned above since they were collected from the field, and also survived well on corn plants.

All samples were subjected to intestinal and ovarian cleaning before DNA and RNA extraction.

### DNA extraction, library preparation, sequencing, and genome assembly and annotation

High molecular weight DNA was extracted using the separated muscle tissue following the protocol recommended by Wang et al (2019). A single tube long fragment read (stLFR) technology (Wang et al., 2019) was used to prepare the co-barcoding DNA libraries using the MGIEasy stLFR Library Prep Kit (lot number: 1000005622). The libraries were sequenced following the MGISEQ-2000 protocol (Huang et al., 2017). A DNA library with a 5 kb insert size was constructed to verify the accuracy of genome assembly and sequenced by BGISEQ-500 sequencer. To finally ligate the scaffolds to chromosomes, Hi-C technology (Lieberman-Aiden et al., 2009) was used to capture the chromosome conformations using one adult FAW. The Hi-C libraries were prepared using the muscle tissues, as described previously in Lieberman-Aiden et al. (2009). Briefly, 1) Chromatin was cross-linked in the intact nucleus with formaldehyde; 2) Restriction endonuclease Hind III was then used for digestion to generate the cohesive terminus; 3) Filling ends of DNA fragments while marking with biotin; 4) Ligation of the neighboring DNA fragments; 5) Purifying and shearing the DNA fragments and performed the biotin pull down and adaptor ligation. The libraries were subjected to BGISEQ-500 sequencer for pair-end sequencing. The Hi-C library provided ~ 167 × (87.49 Gb) sequencing data.

Before the genome assembly, we did the k-mer based genome survey analysis by using the GCE software (v1.0.0) (Liu et al., 2013) with the parameter: -k 17 -a 0 -o 0 -d 0 -t 8 -c 0.9 (Fig. S5 and Table S5). The primary genome was assembled using the supernova (v2.1.1) software (Weisenfeld et al., 2017), *–maxreads* = *700M*. Gaps were filled using GapCloser (Luo et al., 2012) and GapCloser stLFR (unpublished method) with default parameters. Finally, chromosome concatenation was performed using the Hi-C generated data through the 3d-DNA pipeline (Dudchenko et al., 2017).

After the complete genome assembly, we performed the annotation pipeline. First, the Repeat Modeler (v1.0.11), LTR finder (v1.0.5) and repeatscount (v1.0.5) methods were used to identify *de novo* repeat motifs by *ab initio* modeling. The repeat motifs were added in the RepBase (Bao et al., 2015) library as known repeat elements. The Repeat Masker (Saha et al., 2008) was then performed to mask the assembly using the combined RepBase library.

Gene prediction was performed using homology-based, RNA-seq based and *de novo* methods. Before the gene prediction, we masked repeat sequences by changing the A/T/C/G to N all across the genome (hard mask). We used Augustus, glimmerHMM, and SNAP for the *de novo* prediction (Table S7). For the homology-based approaches, *Bombyx mori*, *Danaus plexippus*, *Drosophila melanogaster*, and *Spodoptera litura* genomes were used for homology alignments using the TblastN. Moreover, gene sets were merged to form a non-redundant gene set with GLEAN for transcripts predicted by RNA-seq. Eventually, functional annotation of the proteins was performed using KEGG annotation.

### Comparative genomics analysis

#### Phylogenetic analysis

Data for comparative genomics analysis contained *Spodoptera frugiperda* and other seven species from NCBI (https://www.ncbi.nlm.nih.gov/) including *B*. *mori*, *Danaus plexippus*, *Drosophila melanogaster*, *Helicoverpa armigera*, *Manduca sexta*, *Plutella xylostella*, and *S*. *litura* (Table S21). Based on the *S*. *frugiperda*-ref MAF alignment sequences from the other seven species, four degenerate sites for the 8 species were extracted to reconstruct the Maximum-likelihood tree using iqtree software with a bootstrap of 1000.

#### Genome synteny analysis of S. frugiperda and S. litura

To identify the syntenic blocks in the whole genome, the coding sequences (CDSs) from *S*. *frugiperda* and *S*. *litura* were extracted using a custom python script, and the pairwise synteny was determined using LAST (Kielbasa et al., 2011). Tandem duplications and weak hits were then removed using module McScan (Python version) (Wang et al., 2012) from jcvi (https://github.com/tanghaibao/jcvi). We extracted a subset of blocks using the options, “–minspan = 30 –simple”. The high-quality syntenic karyotype was visualized by McScan from jcvi using the command, “python-m jcvi.graphics.karyotype seqids layout”.

#### Identifying gene families

The specific gene families of eight species were identified using the HMMER3 (https://hmmer.janelia.org/) software which obtain *ABC* domain (PF00005), *COE* domain (PF000135), Cytochrome P450 (*CYP*) domain (PF00067), *GST* domain (PF00043), *UGT* domain (PF00201), *AOX* domain (PF01315), *CHIA* domain (PF00704), *EPHX* domain (PF06441), *PIF* domain (PF05970), *PTCHD* domain (PF02460), and *PTP* domain (PF00782), respectively. The Pfam-A dataset was downloaded from PFAM (https://pfam.janelia.org/) using the hmmscan program. The domain file was used as the first template to scan the gene family whereby the output genes were filtered out with an E-value lower than 1e−10. The filtered genes were used as second templates for another scanning of the gene families. Similarly, the second phase of output genes was filtered out with an E-value lower than 1e−10. Finally, the putative genes from the gene family were identified.

#### Analyzing and defining the CYP gene family

The putative *CYP* gene family of eight species databases was carried out using the HMMER3 software as in section “Identifying gene families”. Since the KEGG annotation result on *CYP* gene was unclear, all the *CYP* genes of the eight species were annotated using the Kobas database (cabbage looper as annotation reference) by blast to reclassify the *CYP* gene subfamily into the following categories: *CYP*2, *CYP*3, *CYP*4, and mito-*CYP*. A large proportion of *CYP* for *S*. *frugiperda* was unannotated, therefore, we classified them as *S*.*fr*-specific *CYP*. According to the identity, the *CYP* sequences were clustered into 152 clusters by CD-HIT-EST (Fu et al., 2012) program with parameter “-c 0.95”.

To confirm the accuracy of *CYP* scanning, the *S. fr*-specific *CYP* set and the *CYP* genes from *B. mori* and *S. litura* were subjected to the MEME program (Bailey et al., 2006) using the command “meme cyp.fasta-dna-o cyp-maxsize 2,000,000”. The motif result and 10 bp flanking sequences were uploaded to the website (https://weblogo.berkeley.edu/logo.cgi) to obtain the motif logo thereby displaying the conservation of the motif.

#### Identifying the positively selected genes (PSGs)

Based on the *S*. *frugiperda*-ref MAF alignment sequences from the *B*. *mori*, single-copy orthologous genes of *S*. *frugiperda* and *B*. *mori* were extracted to perform the positive selection analyses. A positive selection in FAW was tested using the PAML 4.9 (Yang 1997). The branch-site model for codon evolution was used with model = 2, and NS sites (a parameter of PAML software) = 2. Significance (*P* < 0.05) of the compared likelihood ratio tests (LRTs) was evaluated by χ^2^ tests using the PAML 4.9 (Yang, 1997). We assumed that the null distribution was a 50:50 mixture of a χ^2^ distribution with the point mass at zero. The QVALUE in R was used to correct for multiple testing. Finally, the PSG was identified with a cutoff of *P* < 0.05.

### Transcriptome analyses

#### Insects

The tested insects were collected from Yuanjiang, Yunnan Province of China (Table S20), the larvae were reared on fresh corn leaves without exposure to any pesticides for multiple generations, and the adults were provided with a 10% honey/water solution in a laboratory under the following controlled conditions of (27 ± 0.5)°C, (70 ± 5)% RH and a photoperiod of 16 h:8 h (L:D).

#### Pesticide exposure

Twenty-three pesticides commonly used in agricultural production were used in the study (Table S16), including ten single chemical pesticides, nine mixture of chemical pesticides and four biological pesticides. The third instar healthy larvae in the same size were selected for the experiment, and the larvae (3rd instar) were treated by adopted leaf—dipping method (Insecticide Resistance Action Committee, IRAC, 2019). In brief, fresh corn leaves (which had not been exposed to any pesticide) were washed clean and then naturally dried at room temperature. Thereafter, the leaves were cut into pieces (2 × 2 cm) with scissors and dipped into different pesticide solutions (for treatments) or solvent (for control) for 10 s with gentle agitation and place to surface-dry on paper toweling, ensure the entire leaf surface is emerged equally and do not allow the leaves to wilt. After drying, the leaf discs treated with pesticide solutions at different concentrations or solvent for control were transferred into Petri dishes (ϕ = 10 cm) as insect food, and the Petri dishes were padded with moist filter paper at the bottom. Each treatment or control group had three replicates that contained 5 larvae each. Every 12 h, the leaf pieces were replaced by fresh ones treated in the same way. After 48 h (72 h for biological pesticides) post exposure, three surviving larvae were collected for each treatment and put into liquid nitrogen for 2 min, then frozen at − 80 °C in a sealed centrifuge tube for RNA extraction.

#### RNA isolation, transcriptome libraries preparation and sequencing

The RNA extraction kit (RNeasy Mini Kit, Qiagen) was used for total RNA isolation. The RNase-free agarose gel was performed to check for contamination. The RNA integrity and purity were measured using Agilent 2100 Bioanalyzer system (Agilent, United States) and NanoDrop Spectrophotometer (THERMO, United States), respectively. The extracted RNA was fragmented into 200–400 bp and reverse transcribed to cDNA for library preparation. The libraries were prepared following the manufacturer’s instructions using the BGISEQ-500 sequencing platform. Pair-end sequencing with 100 bp in length was performed using the BGISEQ-500 sequencer with the processed libraries.

#### Bioinformatics analysis for transcriptome data

Raw data were initially processed using the Trimmomatic with the parameters of “ILLUMINACLIP:adapter.fa:2:30:4:1:true LEADING:3 TRAILING:3 SLIDINGWINDOW:5:15 MINLEN:50”, and the SOAPfilter was used to filter reads with adaptors, reads with the proportion of Ns larger than 10%, and reads with low-quality bases larger than 10% (Luo et al., 2012). Bridger software (v20141201) was used in *de novo* assembly of the transcriptomes. The redundancy was thereafter removed by TGICL (Pertea et al., 2003). The contigs were concatenated into scaffolds and further assembled to unigenes by clustering and removing redundancy. FPKM was calculated to estimate the expression level of the unigenes. The reads were mapped against the unigene library using Bowtie with the default parameters (Langmead 2010). The unique mapped reads were then selected to estimate the expression level using combining eXpress. For the pesticide treatment samples, the DEG between every treatment with control and different combination of treatment with control was calculated. Also, the DEG of 78 samples were calculated using the edgeR and limma R packages. The *P* value < 0.05 were identified as DEGs. Unsupervised hierarchical clustering (Eisen et al., 1998) was carried out by cluster 3.0 software using uncentered Pearson correlation and complete linkage, and showed by Java Treeview software (Saldanha, 2004) into the “METHODS” section to describe the criterium of clustering.

### Identifying the strains and possible source of FAW in China

*Tpi* gene was used as the DNA marker to identify strains of FAW from the USA, Africa and China in our study. There are eight strain-specific sites (TpiE4-129, TpiE4-144, TpiE4-165, TpiE4-168, TpiE4-180, TpiE4-183, TpiE4-192, TpiE4-198) in the *Tpi* gene (Nagoshi et al., 2019), but the TpiE4-183 site is the most effective diagnostic marker to distinguish C or R strain. Therefore, we finally used this site to do the identification while other sites were also genotyped to assist the strain determination. We used our assembled genome as the reference, and mapped raw reads of all the 240 individuals (including 163 resequencing samples, 72 RNA-seq samples used for pesticide resistance analysis, one sample used for genome assembly survey, one sample used for stLFR genome assembly, one sample used for Hi-C sequencing and two RNA-seq samples used for gene annotation) to the reference genome by using the software BWA (Version: 0.7.10-r789) (Li et al., 2009). Duplicated reads, reads with mapping quality less than 30 and non-unique mapped reads were further filtered out to obtain the finally mapping result for the identification of genotypes of these eight sites. If the genotype is C/C, we identify it as the C strain. Similarly, the T/T and C/T is identified as R strain and the hybrid strain.

### Resequencing analyses

#### Study populations

We used 163 samples in six populations from America (39, A), South Africa (5, SA), Ethiopia (10, ET), Kenya (9, KE), Guangxi (56, GX), Yunnan (44, YN), respectively, for the resequencing analyses (Figs. 4A, S8B and Table S20).

#### \Variant calling and SNP filtering

Paired-end resequencing reads were mapped to our FAW assembly with BWA (Version: 0.7.10-r789) (Li and Durbin, 2009) using the default parameters. SAMtools (Version: 1.3.1) software (Li et al., 2009) was used to convert mapping results into the BAM format and filter the unmapped and non-unique mapping reads. Duplicated reads were filtered using the Picard package (picard.sourceforge.net, Version: 2.1.1). The reads around the InDels were realigned using the Genome Analysis Toolkit (GATK, version 3.3-0-g37228af) in two steps (Mckenna et al., 2010). The first step involved using the RealignerTargetCreator package to identify regions that required realignment. In the second step, the InDelRealigner was used to realign the regions highlighted in the first step. This, therefore, generated a realigned BAM file for each accession.

The variation detection followed the best practice workflow recommended by GATK (Mckenna et al., 2010). The variants were called for each accession by the GATK HaplotypeCaller (Mckenna et al., 2010). A joint genotyping step for comprehensive variations union was performed on the gVCF files. In the filtering step, the SNP filter expression was set as QD < 2.0 || MQ < 40.0 || FS > 100.0 || SOR > 5.0 || MQRankSum <  − 12.5 || ReadPosRankSum <  − 8.0 || QUAL < 30, and the InDel filter expression was set as QD < 2.0 || ReadPosRankSum < 10.0 || InbreedingCoeff < 0.8 || FS > 100.0 || SOR > 5.0 || QUAL < 30. Specific insertions and deletions shorter than or equal to 40 bp were considered. InDels and SNPs with none bi-allelic, > 40% missing calls, and MAF < 0.005 were removed to yield the basic set. SNPs with MAF < 0.05 (the core set) were further removed for phylogenetic tree structure, IBD calculation, PCA, and population structure analyses.

SNPs and InDels annotation was performed for FAW genome using the package ANNOVAR (Version: 2015-12-14) (Wang et al., 2010). The coverage of each accession against each chromosome of FAW genome was counted based on the aligned BAM file using SAMtools (Version: 1.3.1) software (Li et al., 2009). SNP density, InDel density, and total genetic diversity across each chromosome were counted with 100 kb sliding window using VCFtools software (v0.1.13) (Danecek et al., 2011).

#### Population genetics analysis

The whole-genome SNPs were used to construct the ML phylogenetic tree with 100 bootstraps using SNPhylo (Version: 20140701) (Lee et al., 2014). *Spodoptera litura* (Fabricius) was used to provide outgroup information at corresponding positions. The tool iTOL (https://itol.embl.de) was used to color the phylogenetic tree. For each group, the uncertain samples were discarded based on the phylogenetic tree for further analyses.

SNPs were pruned using PLINK (Version v1.90b3.38) (Purcell et al., 2007) with a window size of 50 SNPs (advancing 5 SNPs at a time) and an r2 threshold of 0.5. The PCA was performed through Genome-wide complex trait analysis (GCTA, version: 1.25.3) software (Yang et al., 2011). The first three eigenvectors were plotted. Population structure was analyzed using the ADMIXTURE (Version: 1.3) program (Alexander et al., 2009) with a block-relaxation algorithm. To explore the convergence of individuals, we predefined the number of genetic clusters *K* from 2 to 8 and ran the cross-validation error (CV) procedure. Default methods and settings were used in the analyses.

The admixture graphs for geographically distinct groups were estimated using the TreeMix (Pickrell and Pritchard, 2012), using a maximum likelihood (ML) method based on a Gaussian model for allele frequency change. The topology changes of the ML trees changes depending on the number of migration events (m) allowed in the model. The values, m = 1 to m = 5 were used. The bootstrap values on the tree were based on 1,000 replicates. Arrows on the graph represented admixture events between different FAW populations.

#### Demographic history reconstruction using PSMC

The demographic history for FAWS from America (*n* = 10, three from AA, three from AB, two from AC, and two from AD), Africa (*n* = 10, four from ET, four from KE and two from SA) and China (*n* = 10, five from YN and five from GX) was inferred using a hidden Markov model approach following the pairwise sequentially Markovian coalescence (PSMC) (Liu and Hansen, 2017) based on SNP distribution. The parameters were set as follows: “-N25 -t15 -r5 -p 4+25*2+4+6”, “-d 13 -D 80”, “-q20”. A 0.25 year generation time and a mutation rate of 2.9 × 10^−9^ mutations per nucleotide per year were used to convert the scaled times and population sizes into real times and sizes.

#### Selection signals analysis

SweeD (Version 3.3.1) (Pavlidis et al., 2013) was used to detect selective sweeps based on the CLR test. This detected signatures for artificial selection or a natural selection from each FAW group with grid number set as chromosome length/1000. CLR cut-off value of AA, AB, AC, AD, ET, KE, SA, GX and YN were: 115.72, 2444.49, 89.40, 265.15, 136.09, 162.07, 280.32, 77.53 and 66.44, respectively.

## Electronic supplementary material

Below is the link to the electronic supplementary material.

(PDF 2974 kb)

## Data Availability

The Raw sequencing data and the chromosome level genome assembly have been deposited to the CNSA (CNGB Nucleotide Sequence Archive) with accession CNP0000513 (https://db.cngb.org/cnsa/). The transcript and resequencing data have been deposited to the CNSA with accession CNP0001020. The genome data are also available through a BLAST webserver (https://159.226.67.243/viroblast/fawmine.php) and genome database (https://159.226.67.243:8080/fawmine).

## ABBREVIATIONS

4DTv, Fourfold degenerate synonymous site; A, America; AA, America_A; AB, America_B; AC, America_C; AD, America_D; *ABC*, ATP-binding cassette; *AOX*, aldehyde oxidase; *Bt*, *Bacillus thuringiensis*; *CES*, carboxylesterase; *CHIA*, chitinase acidic; *CYP*, cytochrome P450; DEG, differential expression gene; *EPHX*, epoxide hydrolase; EST, Expressed sequence tags; ET, Ethiopia; FAW, fall armyworm; *GST*, glutathione S-transferase; GX, Guangxi; Hi-C, high-throughput chromosome conformation capture; InDel, insertion and deletion; KE, Kenya; Kya, thousand years ago; LRT, likelihood ratio tests; MAF, minor allele frequency; ML, maximum likelihood; Mya, million years ago; PCA, principal component analysis; PIF, PIF1 5′-to-3′ DNA helicase; PSG, Positively selected gene; PSMC, pairwise sequentially Markovian coalescen; *PTCHD*, patched domain containing; *PTP*, protein tyrosine phosphatase; SA, South Africa; SNP, single nucleotide polymorphism; stLFR, single tube long fragment read; *UGT*, UDP-glucuronosyl transferase; YN, Yunnan
