# Supplementary material for: Genomic and transcriptomic analysis unveils population evolution and development of pesticide resistance in fall armyworm Spodoptera frugiperda
Source: Protein Cell. 2020 Oct 27;13(7):513–31. doi: 10.1007/s13238-020-00795-7 (PMC9226219; doi:10.1007/s13238-020-00795-7)
Supplement: Supplementary file 1 — (PDF 2974 kb) [file 13238_2020_795_MOESM1_ESM.pdf]

Supplementary Materials for

Genomic and transcriptomic analysis unveils population evolution and development of pesticide resistance in fall armyworm *Spodoptera frugiperda*

Furong Gui[1,12,*], Tianming Lan[2,9,*], Yue Zhao[1,*], Wei Guo[3,15,*], Yang Dong [1,12], Dongming Fang[2], Huan Liu[2,9], Haimeng Li[2], Hongli Wang[2], Ruoshi Hao[12], Xiaofang Cheng[5], Yahong Li[16], Pengcheng Yang[4], Sunil Kumar Sahu[2], Yaping Chen[1], Le Cheng[7], Shuqi He[1], Ping Liu[5], Guangyi Fan[6], Haorong Lu[8,10], Guohai Hu[8,10], Wei Dong[2], Bin Chen[1], Yuan Jiang[18], Yongwei Zhang[18], Hanhong Xu[17]Fei Lin[17], Bernard Slipper[19], Alisa Postma[19], Matthew Jackson[19], Birhan Addisie Abate[20], Kassahun Tesfaye[20,21], Aschalew Lemma Demie[20], Meseret Destaw Bayeleygne[20], Dawit Tesfaye Degefu[22], Feng Chen[5], Paul K Kuria[23], Zachary M Kinyua[23], Tong-Xian Liu[13], Huanming Yang[10,11], Fangneng Huang[14]†, Xin Liu[2,10,]†, Jun Sheng[1,12,]†, Le Kang[3, 4,15,]†

* Furong Gui, Tianming Lan, Yue Zhao and Wei Guo have contributed equally to this work.
†Corresponding authors. Email: Le Kang: ***lkang@ioz.ac.cn***, Jun Sheng: ***shengjunpuer@163.com*** , Xin Liu: ***liuxin@genomics.cn***, Fangneng Huang: *FHuang@agcenter.lsu.edu*

SUPPLEMENTARY FIGURES

Continent		Countries and regions involved	Countries and regions FAW occurred
South America	Northern South America	5	5
	Central South America	3	3
	Eastern South America	1	1
	Southern south America	4	4
North America	North America	4	3
	Central America	7	7
	Caribbean	25	13
Africa	North Africa	8	2
	East Africa	10	10
	Central Africa	8	7
	West Africa	18	15
	South Africa	15	15
Asia	West Asia	20	3
	South Asia	7	5
	Southeast Asia	11	11
	East Asia	5	3
	Central Asia	5	0
Oceania		24	2
Europe		44	0
Total		224	109

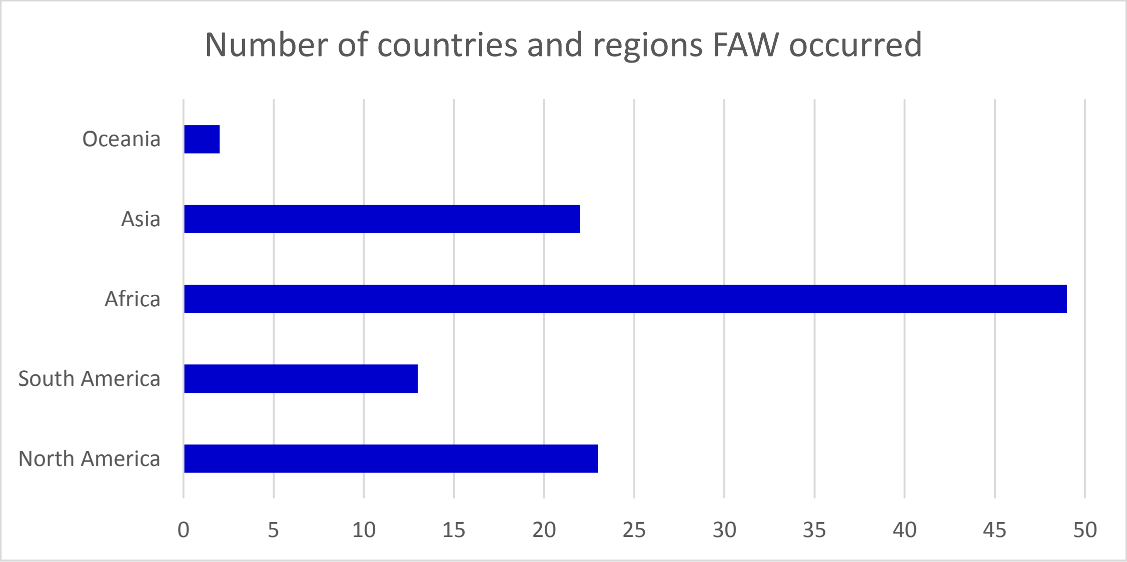

Figure S1 | The world distribution of the Fall armyworm (FAW).

Data source: Invasive Species Compendium. https://www.cabi.org/isc/fallarmyworm.

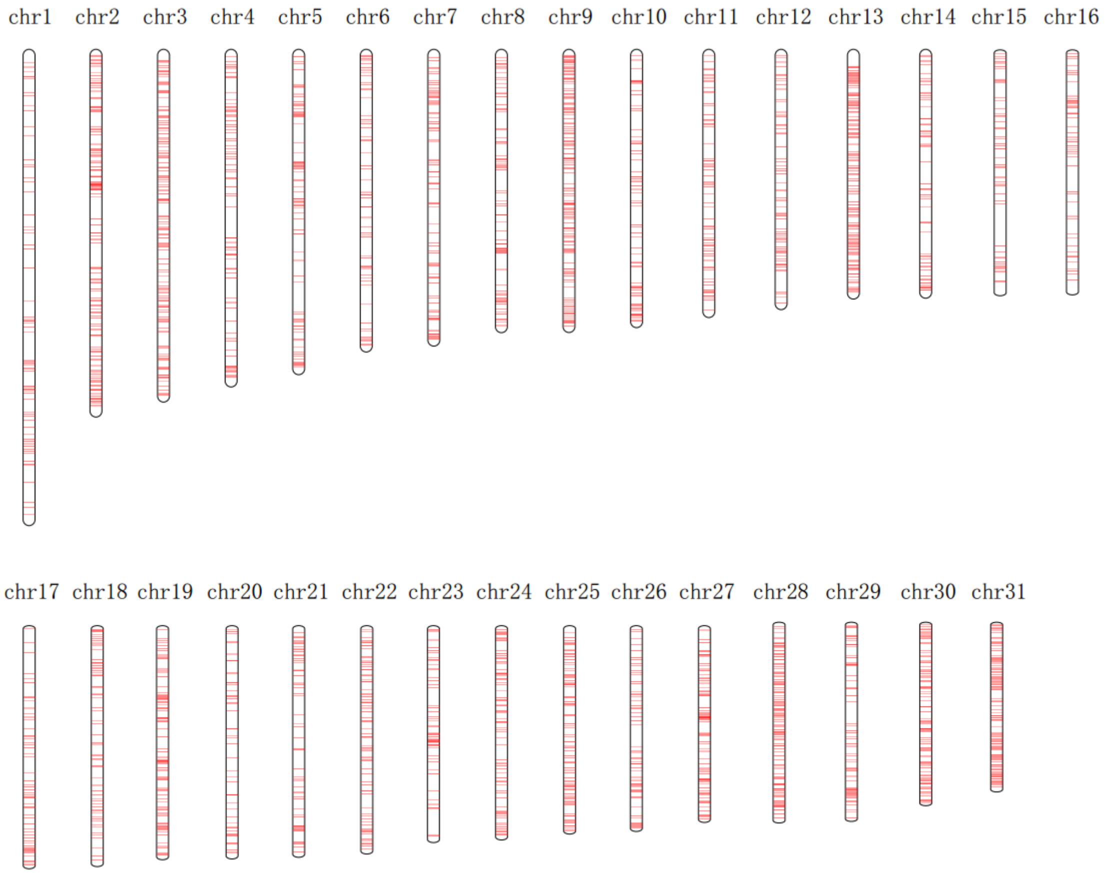

Figure S2 | The distribution of extra regions in 31 chromosomes of fall armyworm.

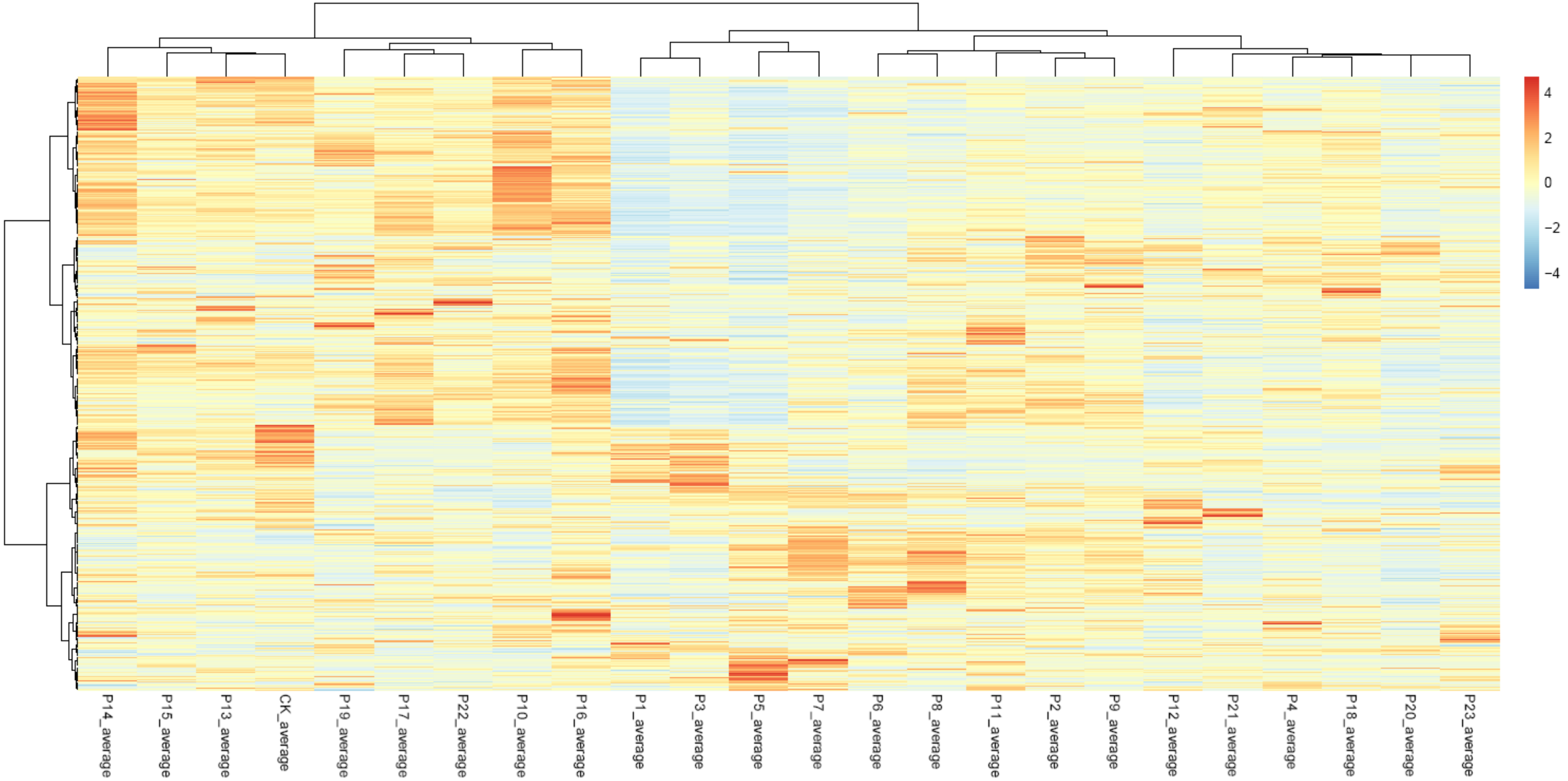

Figure S3 | The heatmap of 1421 expressed gene between 23 pesticide treatments and control.

Note: The heatmap shows the average value of FPKM; P1-23 represent the 23 pesticides.

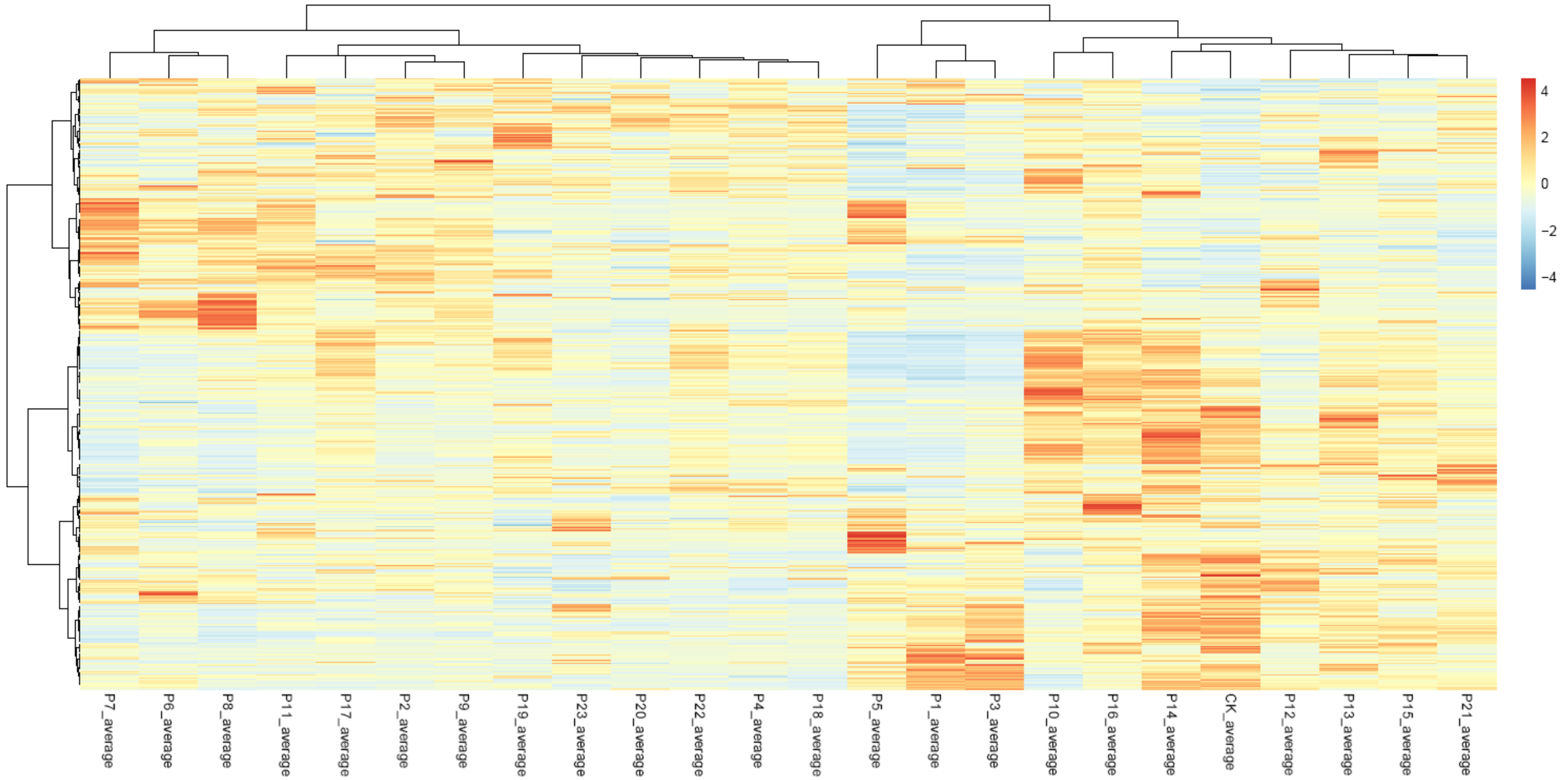

Figure S4 | The heatmap of 512 differential expression genes (DEGs) between 23 pesticide treatments and control.

The heatmap shows the average value of FPKM; P1-23 represent the 23 pesticides.

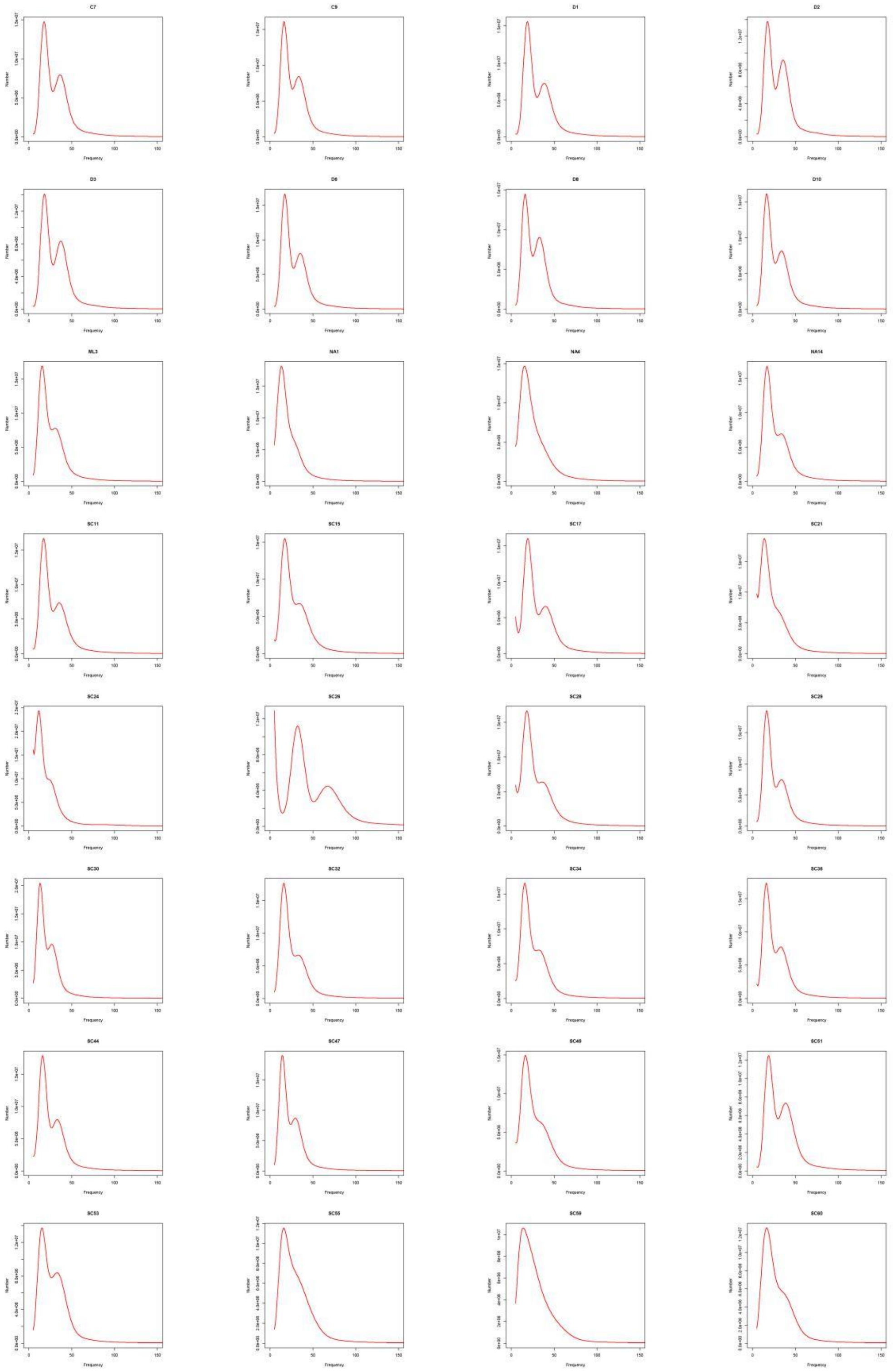

Figure S5 | K-mer based genome survey results calculated by the GCE software.

Note: 32 individuals with the sequencing depth larger than 60× were selected for this genome survey analysis.

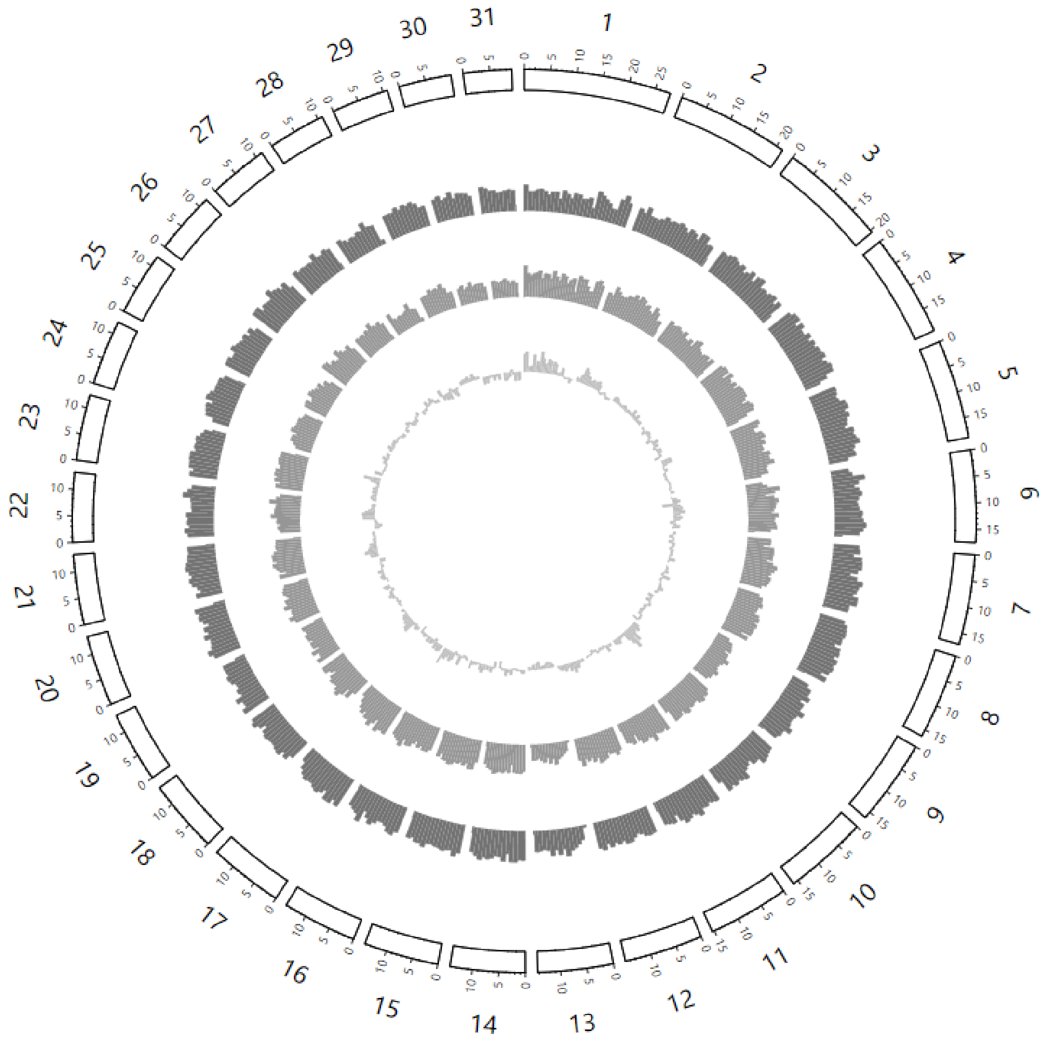

Figure S6 | The single nucleotide polymorphism (SNP) density, total genetic diversity (π) and Tajima's D across all chromosomes of the genome scaled to 1Mb size.

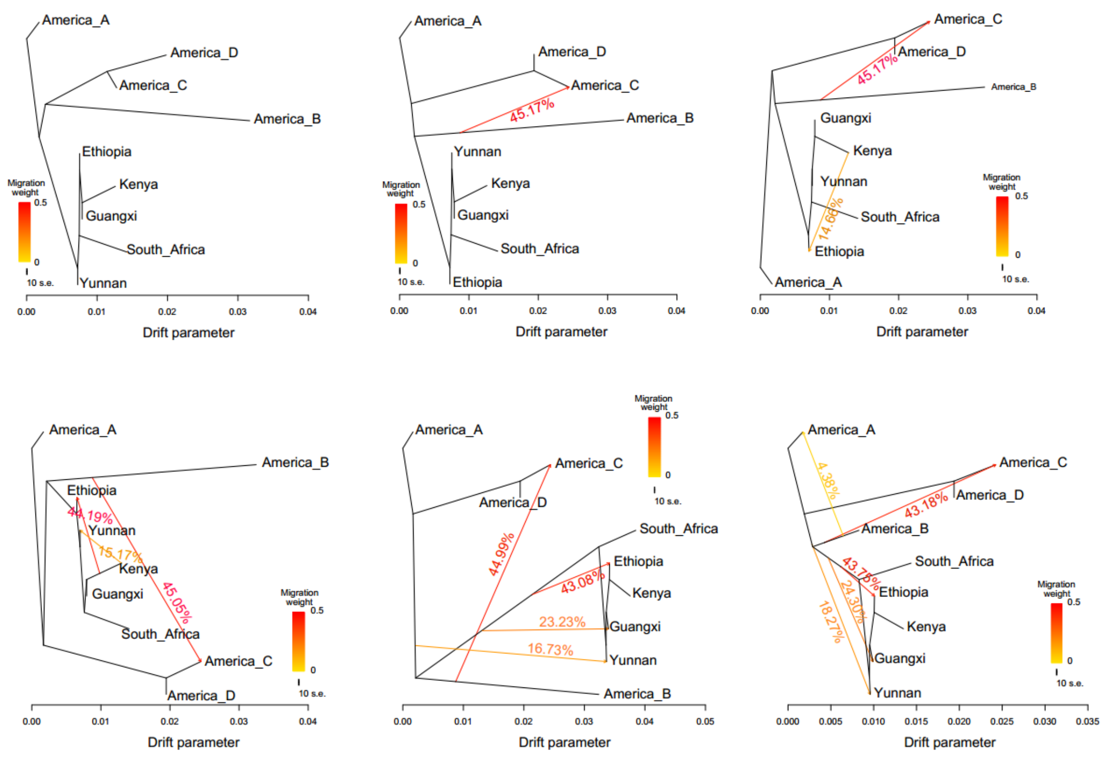

Figure S7 | The TreeMix results for models with m = 0 to m = 5.

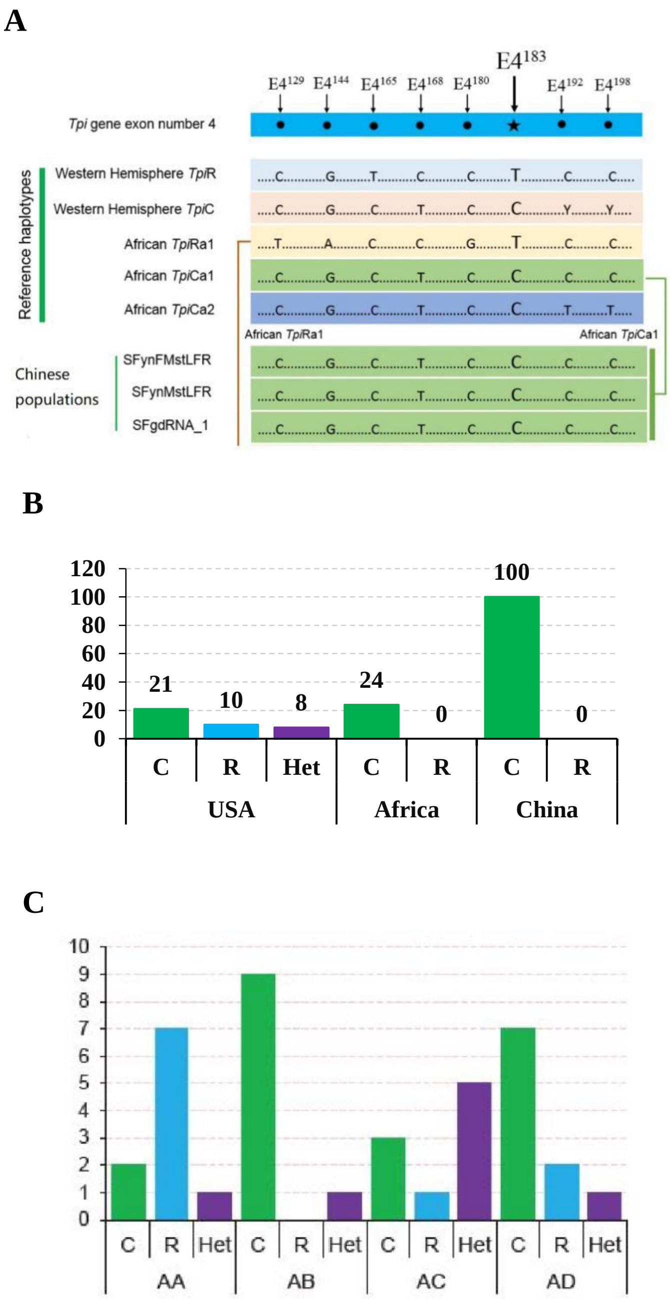

Figure S8 | Identification of C or R strain of FAW from USA, Africa and China.

A: To facilitate the understanding of identifying of C and R strain, we selected 5 reference samples to show the differences of strain-specific sites between different subtypes. We also showed these strain-specific site of Chinese populations. **B**: The statistics of the distribution of C and R strain in USA, Africa and China populations. If the TpiE4-183 is found to be heterozygous, we identified them as hybrid individuals. **C**: The statistics of the distribution of C and R strain in each population of USA. (C: C strain; R: R strain; Het: Hybrids of C and R strains; AA: American population A; AB: American population B; AC: American population C; AD: American population D).

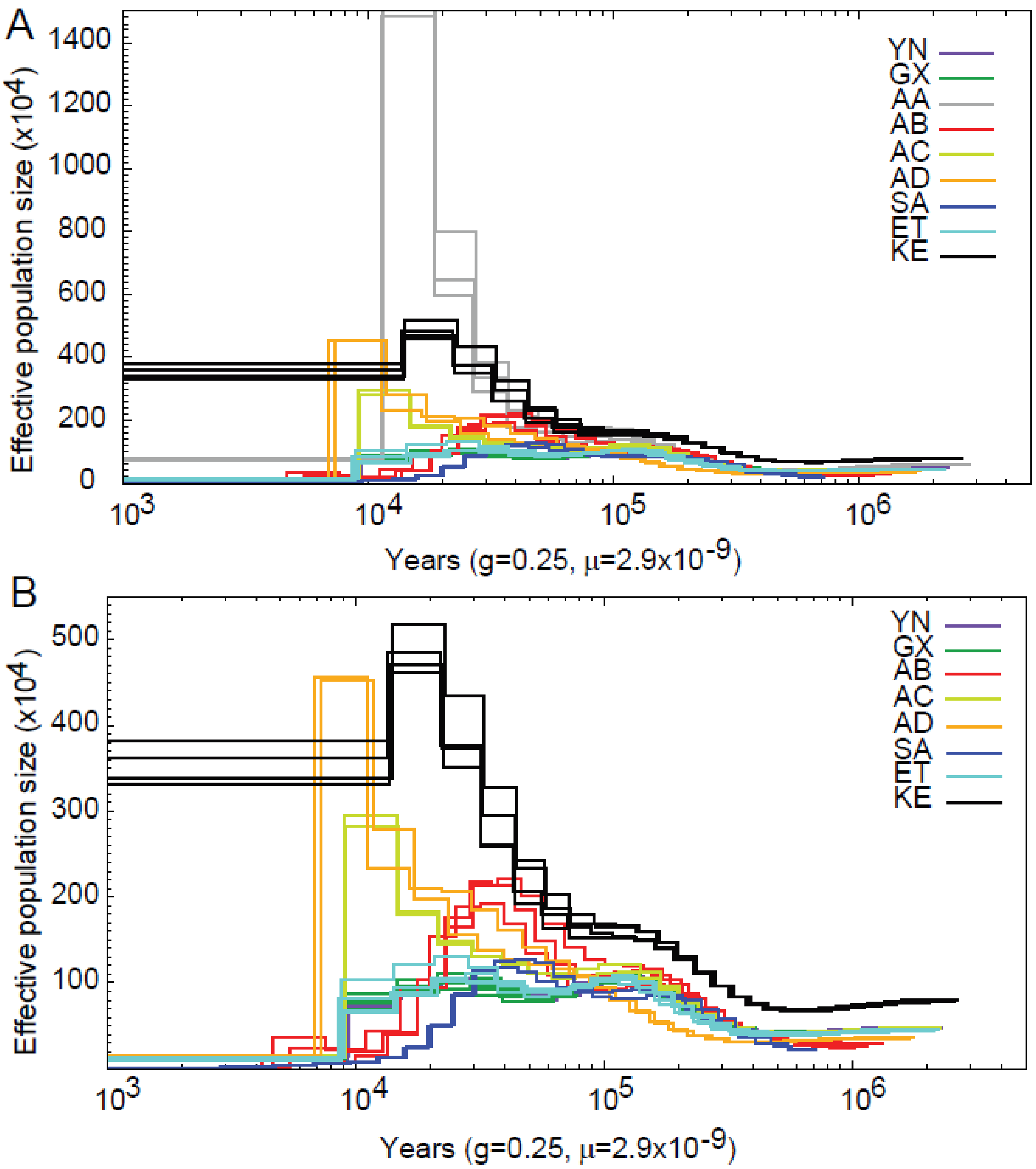

Figure S9 | The population demography analysis of FAW populations inferred by PSMC.

A: The PSMC results from all nine populations, including samples from America (n = 10, three from AA, three from AB, two from AC, and two from AD), Africa (n = 10, four from ET, four from KE and two from SA) and China (n = 10, five from YN and five from GX). B: To facilitate showing more detailed information, we removed the AA population from the picture A. (PSMC: pairwise sequentially Markovian coalescent; AA: American population A; AB: American population B; AC: American population C; AD: American population D; ET: Ethiopia; KE: Kenya; SA: South Africa; YN: Yunnan; GX: Guangxi.)

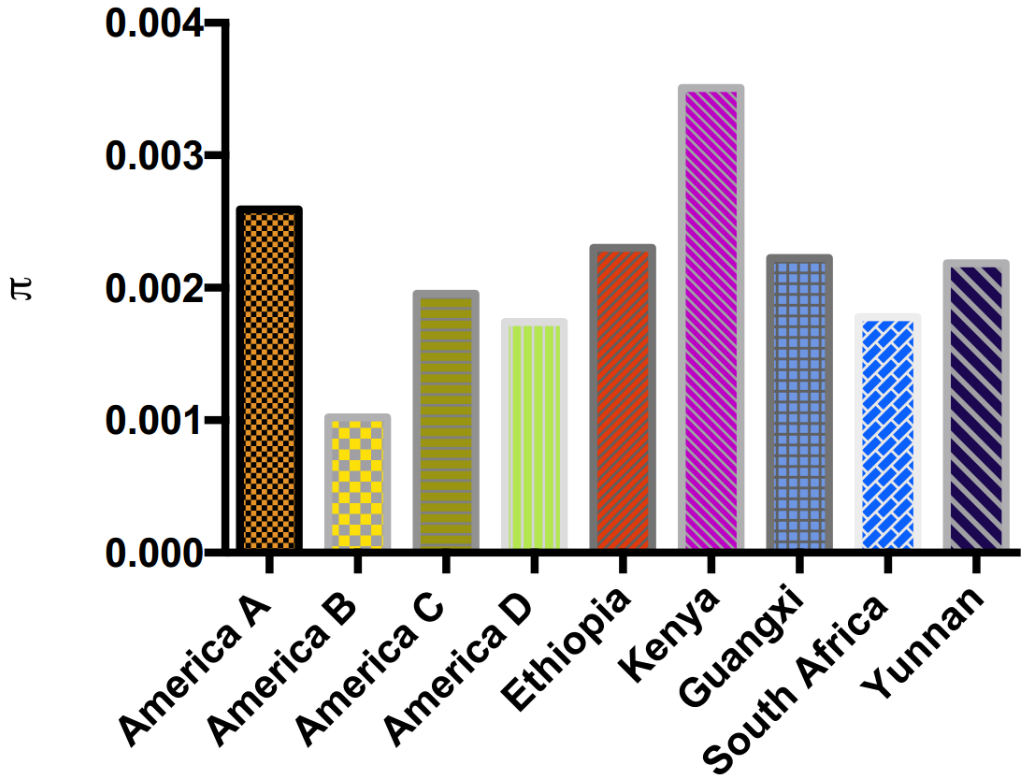

Figure S10 | The global nucleotide diversity (*π*) of nine accessions.

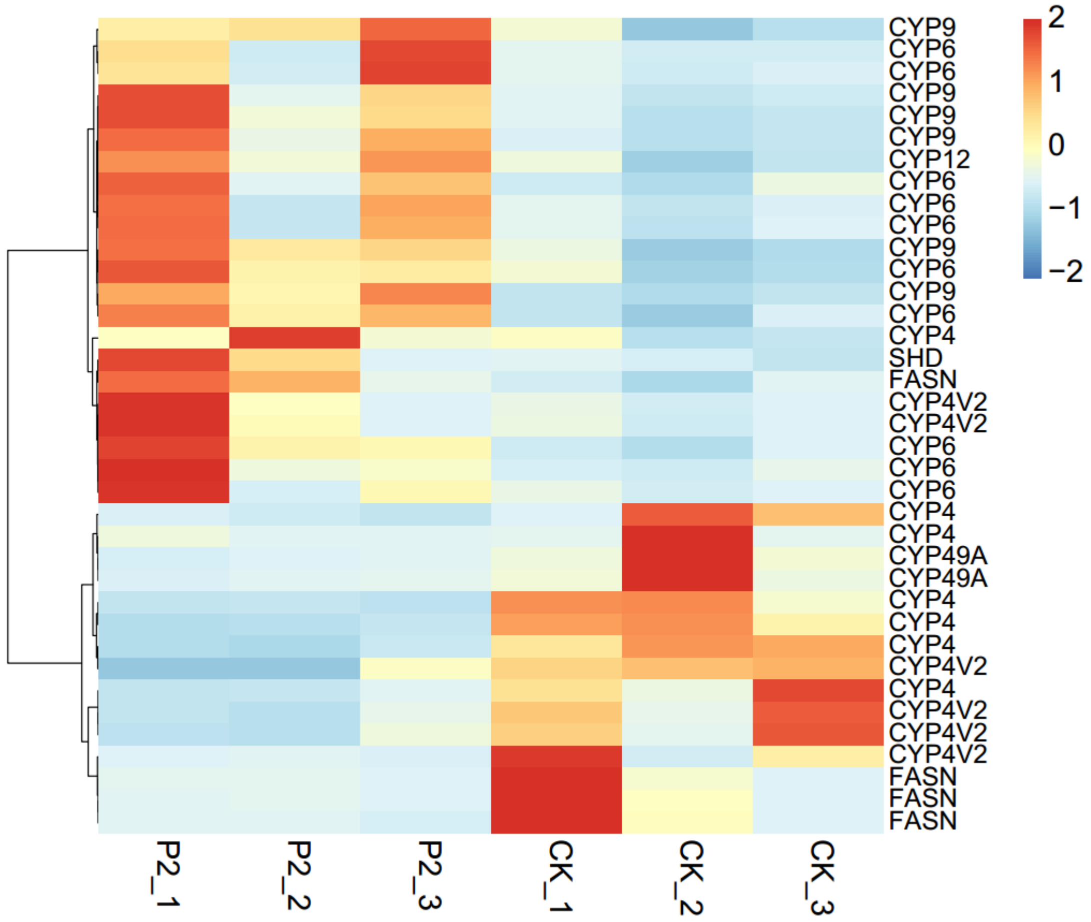

Figure S11 | Differentially expressed *CYP* genes after *Bacillus thuringiensis* (*Bt*) treatment.

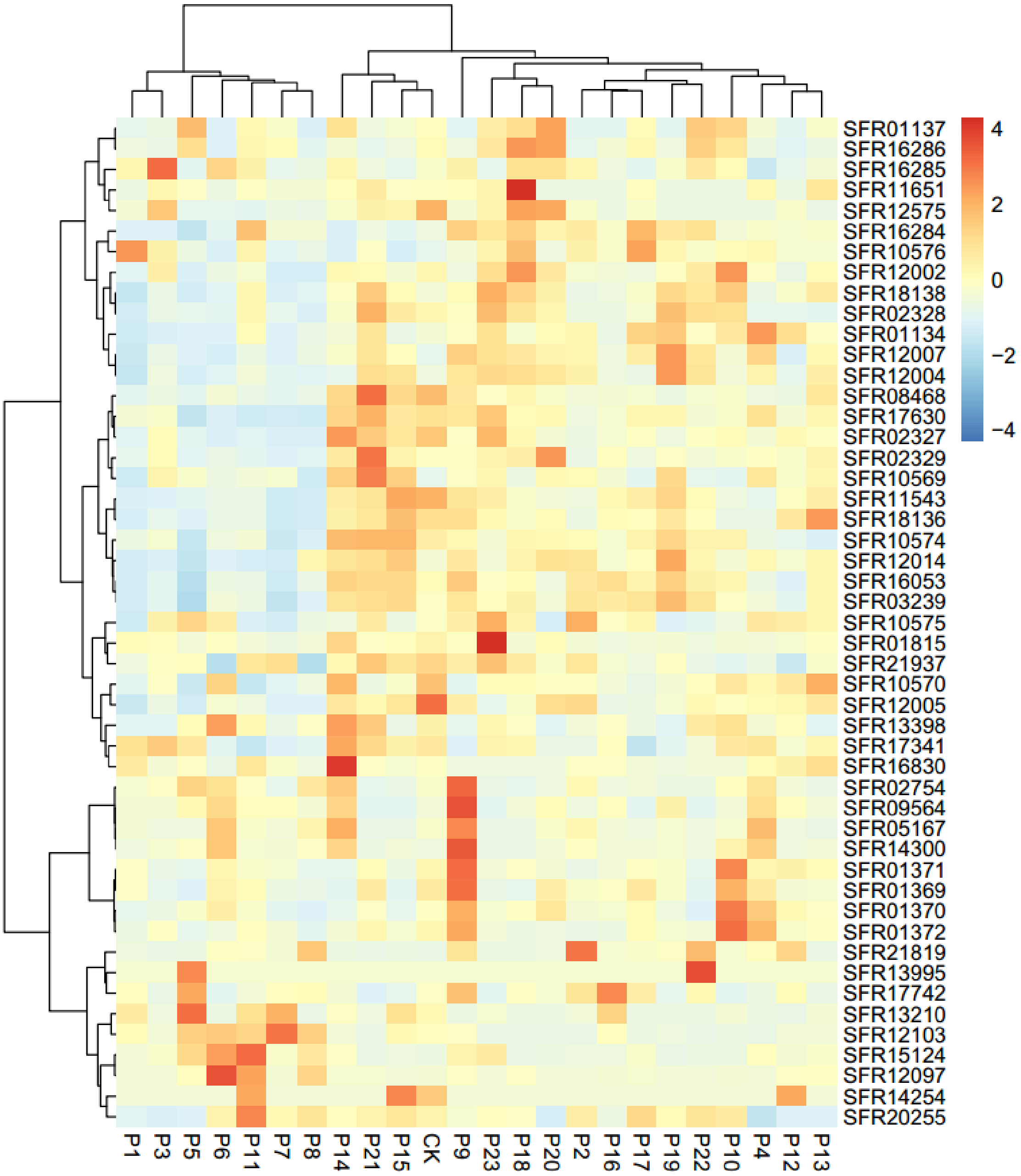

Figure S12 | The expression of *UGT* genes between all 23 treatments and control. The heatmap shows the average value of FPKM.

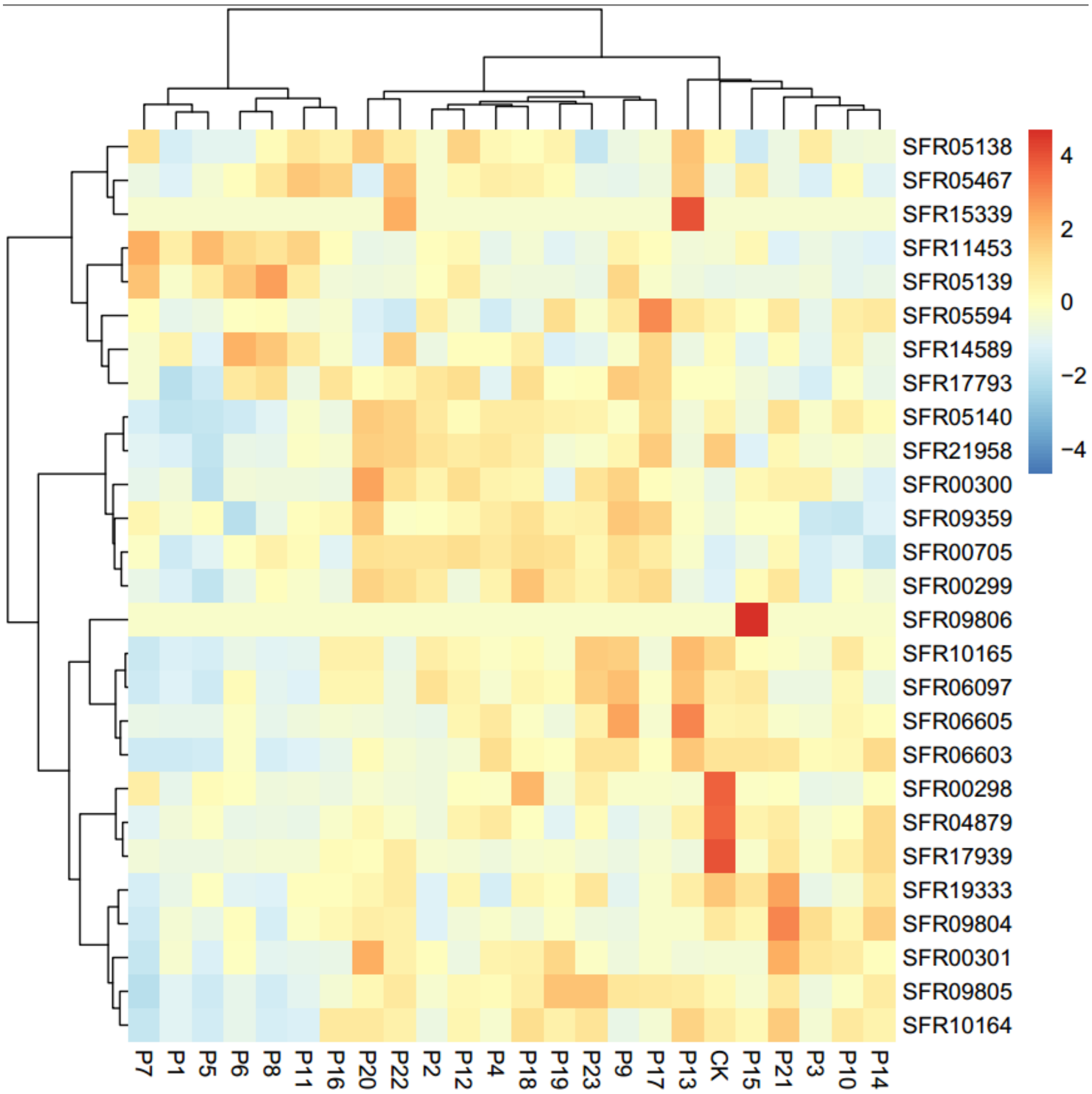

Figure S13 | The expression of *GST* genes between all 23 treatments and control. The heatmap shows the average value of FPKM.

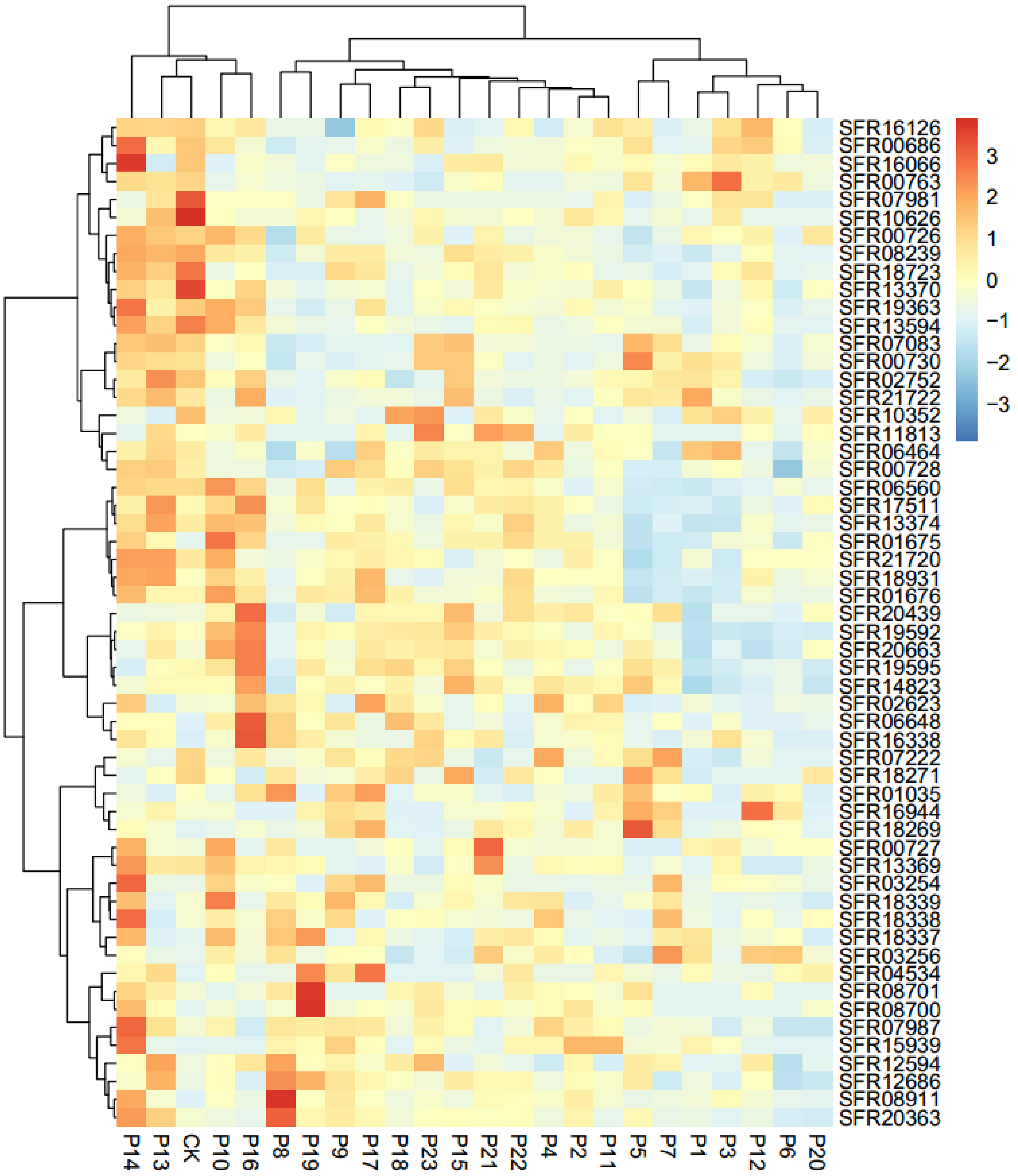

Figure S14 | The expression of *ABCC4* and *ABCA3* genes between all 23 treatments and control.

The heatmap shows the average value of FPKM.

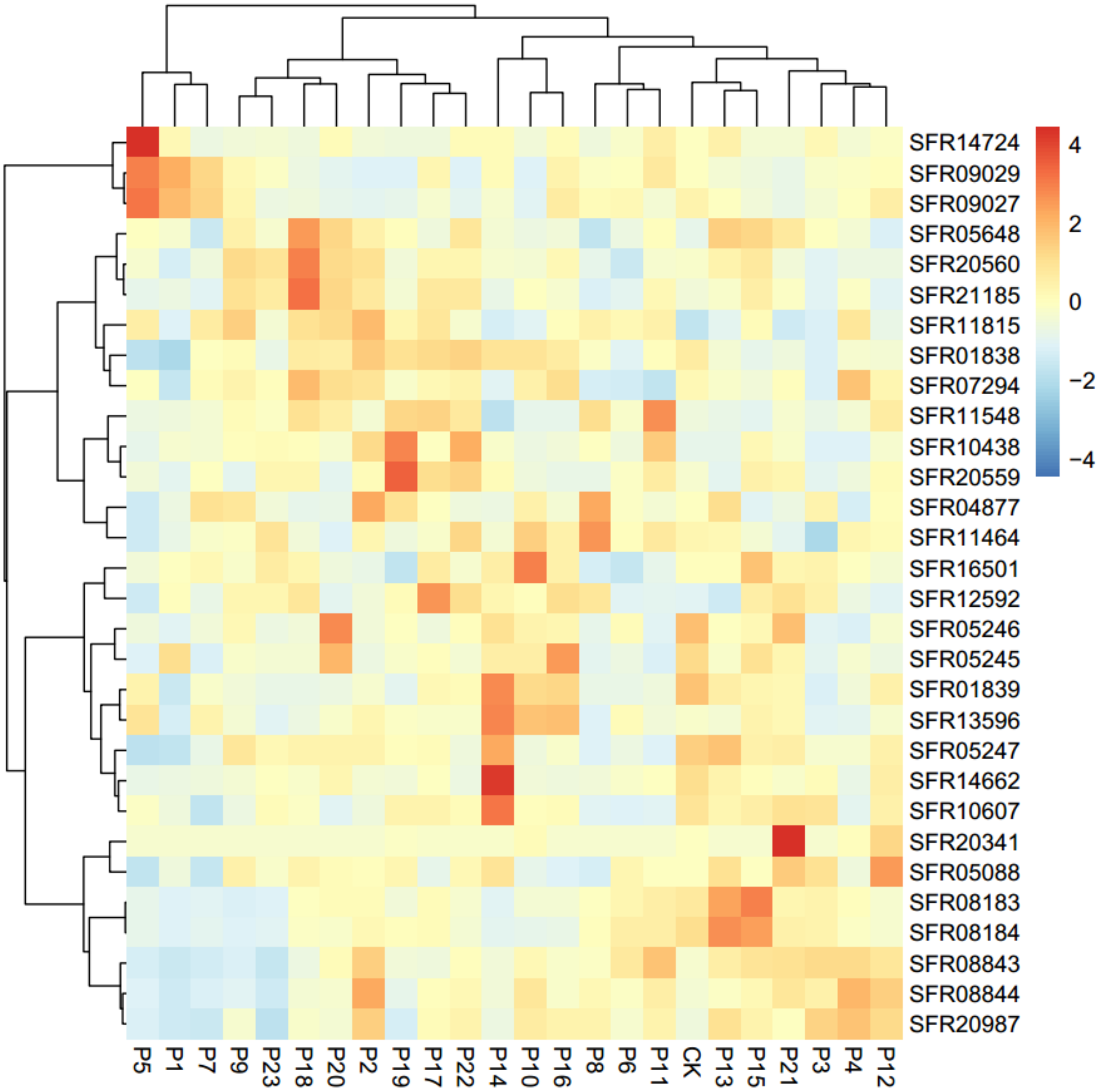

Figure S15 | The expression of *CES1* and *CES2* genes between all 23 treatments and control.

The heatmap shows the average value of FPKM.

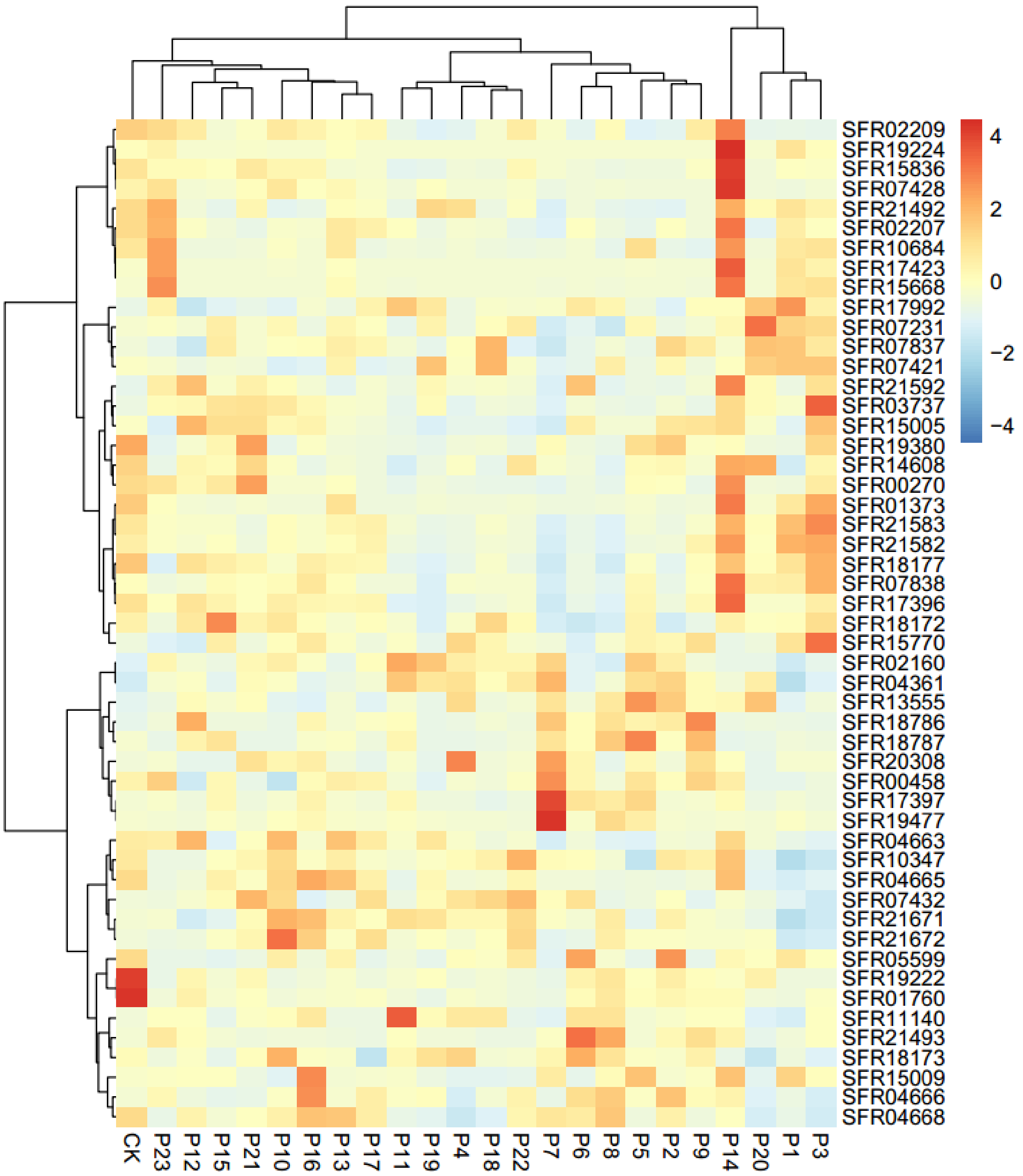

Figure S16 | The expression of *SLC22A3*, *SLC22A4* and *SLC22A5* genes between all 23 treatments and control.

The heatmap shows the average value of FPKM.

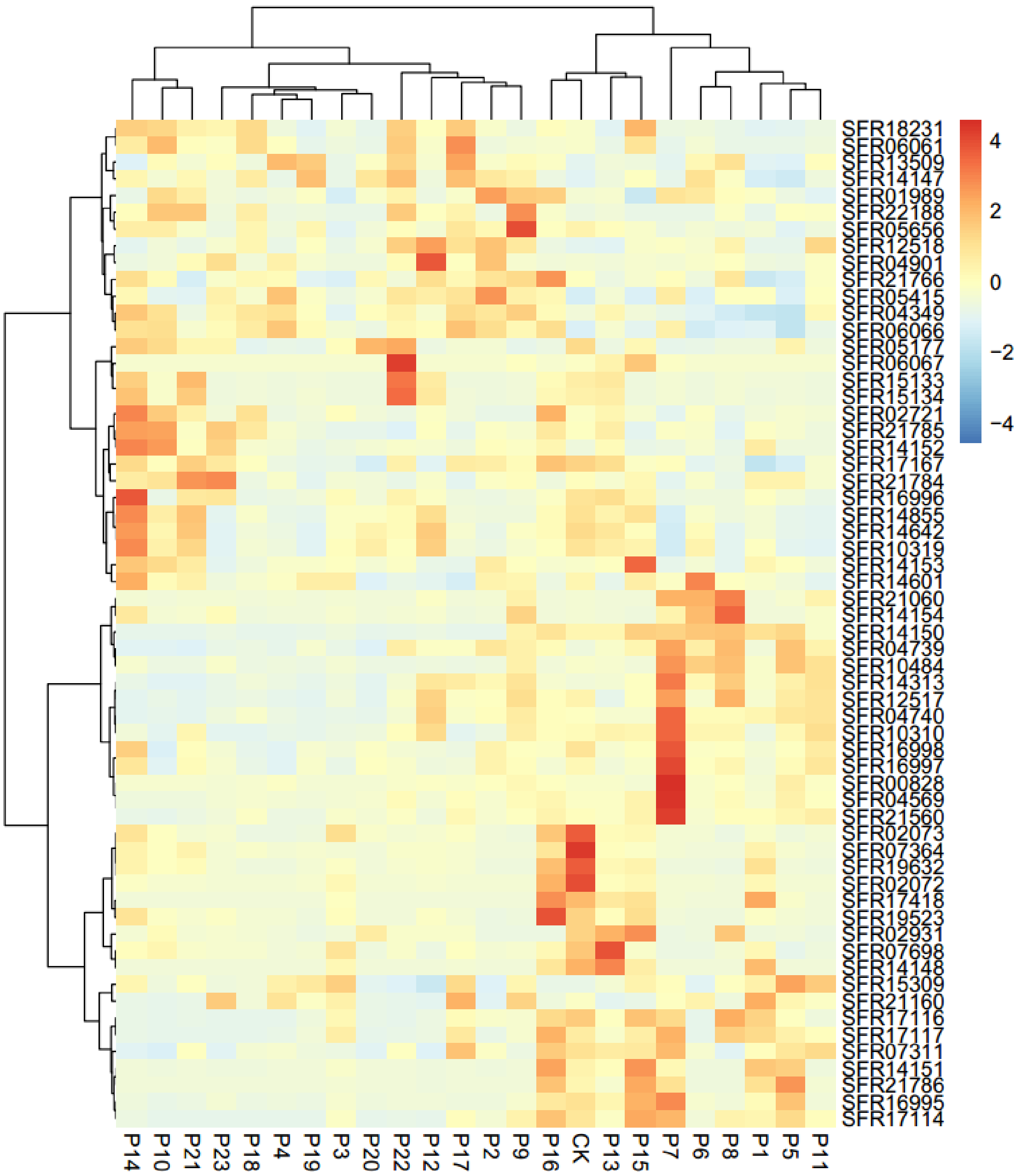

Figure S17 | The expression of *ChuaMOX* genes between all 23 treatments and control.

The heatmap shows the average value of FPKM.

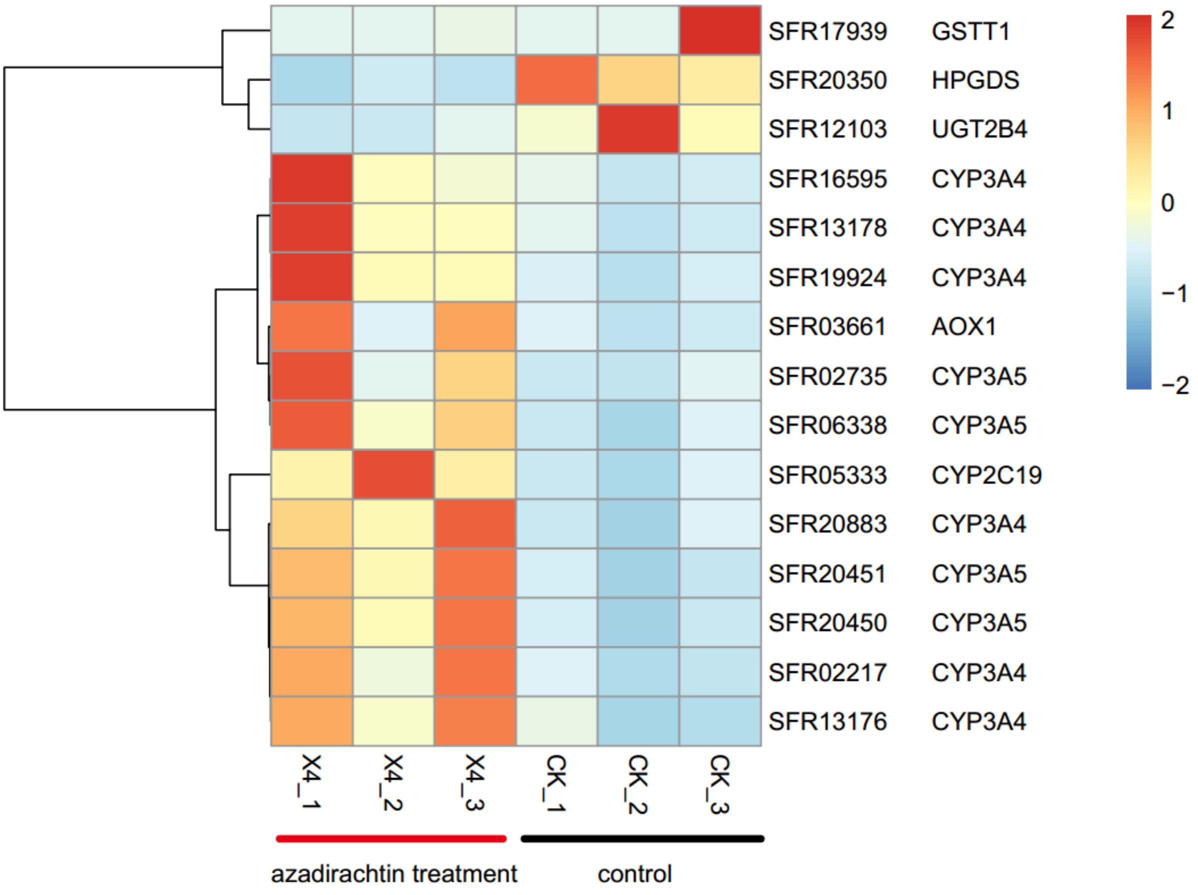

Figure S18 | Fifteen differential expression genes that involved in hsa00982 (Drug metabolism - cytochrome P450) between azadirachtin treatment and control.

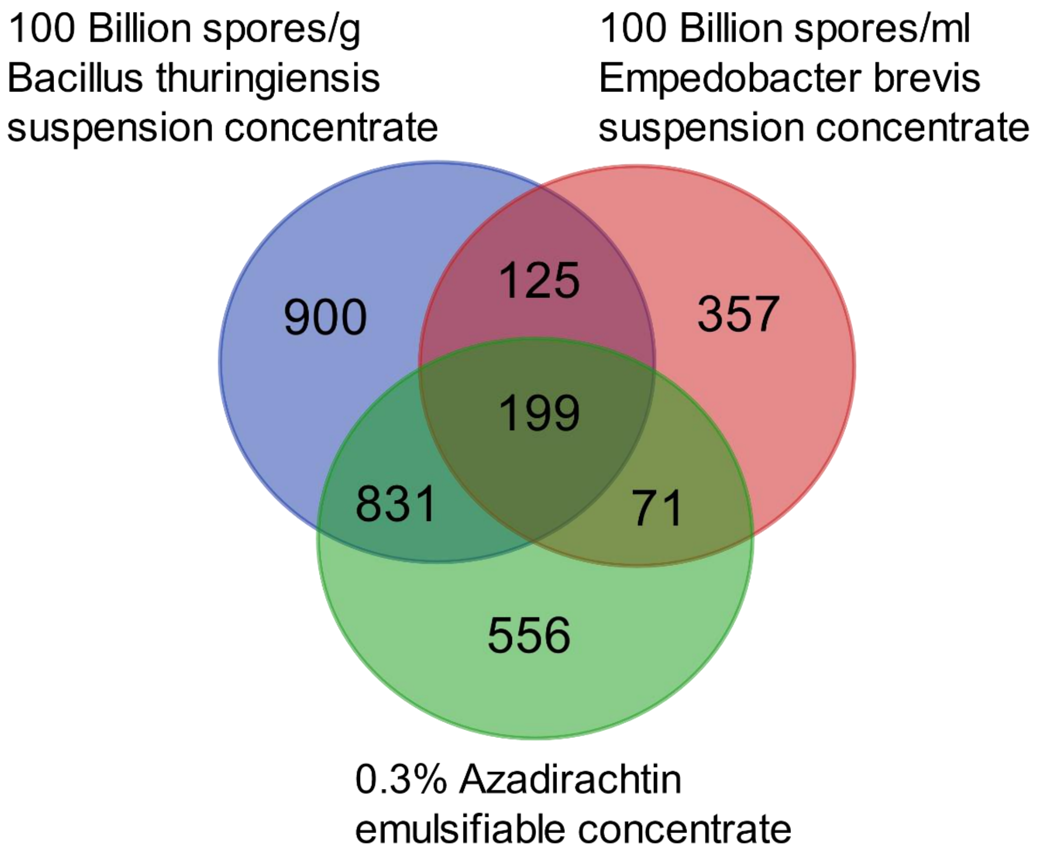

Figure S19 | The shared differential expression genes between *Bacillus thuringiensis* (Bt), *Empedobacter brevis* and azadirachtin aim at the digestive system of FAW.

SUPPLEMENTARY TABLES

Table S1 | Summary of the FAW genome sequencing data.

	Sample Identifier	LibraryType	LibraryInsertSize	ReadNum (M)	BaseNum(G)	GC%	Q10	Q20	Q30(total)
WGS	SFgdWGS	Pair-end	250bp	673.92	134.78	36.74	98.55	95.04	85.91
stLFR	SFynMstLFR	Pair-end	300bp	463.49	56.08	39.76	97.51	92.85	82.31
RNA-seq	SFgdRNA_1	Pair-end	300bp	747.45	149.49	48.92	99.08	96.51	88.60
RNA-seq	SFgdRNA_2	Pair-end	300bp	680.55	136.11	45.43	98.73	95.59	87.16
Hi-C	SFYN	Pair-end	350bp	874.92	87.49	39.76	97.51	92.14	80.78
WGS	A1	Pair-end	250bp	86.51	17.30	36.93	99.79	98.66	92.63
WGS	A10	Pair-end	250bp	125.92	25.18	37.60	99.85	98.92	93.46
WGS	A2	Pair-end	250bp	78.87	15.78	37.25	99.76	98.36	91.36
WGS	A3	Pair-end	250bp	97.29	19.46	37.14	99.84	98.97	94.03
WGS	A4	Pair-end	250bp	100.54	20.10	37.61	99.76	98.40	91.56
WGS	A5	Pair-end	250bp	111.20	22.24	37.44	99.76	98.44	91.84
WGS	A6	Pair-end	250bp	88.47	17.70	36.45	99.80	98.68	92.81
WGS	A7	Pair-end	250bp	95.51	19.10	37.69	99.76	98.40	91.53
WGS	A8	Pair-end	250bp	94.64	18.92	39.55	99.79	98.70	92.85
WGS	A9	Pair-end	250bp	95.58	19.12	37.29	99.81	98.78	93.20

WGS	B1	Pair-end	250bp	132.56	26.52	36.98	99.87	99.06	94.13
WGS	B10	Pair-end	250bp	144.66	28.94	37.82	99.85	98.89	93.35
WGS	B2	Pair-end	250bp	150.02	30.00	38.24	99.86	98.97	93.68
WGS	B3	Pair-end	250bp	125.21	25.04	37.73	99.86	99.01	93.91
WGS	B4	Pair-end	250bp	134.07	26.82	37.65	99.86	98.96	93.63
WGS	B5	Pair-end	250bp	156.40	31.28	37.73	99.86	98.96	93.58
WGS	B6	Pair-end	250bp	138.03	27.60	40.34	99.84	98.81	93.06
WGS	B7	Pair-end	250bp	139.86	27.98	37.38	99.85	98.88	93.28
WGS	B8	Pair-end	250bp	137.69	27.54	37.33	99.87	99.03	94.00
WGS	B9	Pair-end	250bp	135.25	27.06	37.12	99.84	98.88	93.28
WGS	BS01	Pair-end	250bp	83.82	16.76	37.38	99.74	98.21	90.81
WGS	BS02	Pair-end	250bp	84.97	17.00	36.73	99.76	98.39	91.59
WGS	C1	Pair-end	250bp	142.08	28.42	38.16	99.85	98.91	93.41
WGS	C10	Pair-end	250bp	147.76	29.56	36.70	99.86	98.96	93.59
WGS	C2	Pair-end	250bp	133.33	26.66	37.27	99.86	98.99	93.78
WGS	C3	Pair-end	250bp	147.10	29.42	37.01	99.88	99.14	94.47
WGS	C4	Pair-end	250bp	150.24	30.04	37.29	99.86	99.00	93.79
WGS	C6	Pair-end	250bp	146.20	29.24	36.65	99.85	98.94	93.50

WGS	C7	Pair-end	250bp	256.95	51.40	36.88	99.85	98.97	93.71
WGS	C8	Pair-end	250bp	137.41	27.48	36.70	99.85	98.90	93.36
WGS	C9	Pair-end	250bp	240.52	48.10	36.85	99.86	99.03	94.10
WGS	D1	Pair-end	250bp	272.39	54.48	36.92	99.85	98.95	93.60
WGS	D10	Pair-end	250bp	255.54	51.10	36.79	99.84	98.91	93.46
WGS	D2	Pair-end	250bp	243.67	48.74	36.21	99.84	98.91	93.44
WGS	D3	Pair-end	250bp	259.89	51.98	36.64	99.85	98.96	93.66
WGS	D4	Pair-end	250bp	252.74	50.54	36.68	99.84	98.84	93.16
WGS	D5	Pair-end	250bp	160.76	32.16	37.36	99.86	99.02	93.93
WGS	D6	Pair-end	250bp	253.03	50.60	36.65	99.86	99.08	94.33
WGS	D7	Pair-end	250bp	142.83	28.56	36.64	99.86	98.94	93.51
WGS	D8	Pair-end	250bp	228.92	45.78	36.80	99.84	98.91	93.48
WGS	D9	Pair-end	250bp	137.58	27.52	37.12	99.86	98.97	93.65
WGS	FAW1.1	Pair-end	250bp	105.83	21.16	37.43	99.74	98.28	91.09
WGS	FAW1.2	Pair-end	250bp	72.91	14.58	38.92	99.73	98.25	91.05
WGS	FAW2.1	Pair-end	250bp	107.93	21.58	40.32	99.78	98.67	92.95
WGS	FAW2.2	Pair-end	250bp	117.21	23.44	36.94	99.78	98.55	92.30
WGS	FAW5.2	Pair-end	250bp	86.36	17.28	36.81	99.78	98.56	92.16

WGS	FJ01_1	Pair-end	250bp	87.89	17.58	41.29	99.78	98.74	93.50
WGS	FJ01_2	Pair-end	250bp	77.03	15.40	36.34	99.83	98.92	93.91
WGS	FJ01_3	Pair-end	250bp	104.32	20.86	38.53	99.79	98.65	92.87
WGS	GX01	Pair-end	250bp	95.12	19.02	36.94	99.75	98.38	91.56
WGS	GX02	Pair-end	250bp	107.35	21.46	37.23	99.77	98.52	92.18
WGS	GX03	Pair-end	250bp	90.26	18.06	36.79	99.75	98.33	91.40
WGS	GX04	Pair-end	250bp	83.71	16.74	37.90	99.80	98.66	92.87
WGS	JC01	Pair-end	250bp	92.00	18.40	37.04	99.77	98.46	92.02
WGS	KE-1	Pair-end	250bp	153.64	30.72	38.23	99.84	98.98	93.88
WGS	KE-10	Pair-end	250bp	162.77	32.56	38.93	99.87	99.09	94.35
WGS	KE-3	Pair-end	250bp	144.90	28.98	37.53	99.87	99.11	94.41
WGS	KE-4	Pair-end	250bp	160.97	32.20	37.39	99.86	99.02	93.98
WGS	KE-5	Pair-end	250bp	146.09	29.22	37.42	99.87	99.13	94.45
WGS	KE-6	Pair-end	250bp	145.32	29.06	37.90	99.87	99.06	94.16
WGS	KE-7	Pair-end	250bp	138.70	27.74	37.37	99.87	99.05	94.13
WGS	KE-8	Pair-end	250bp	134.57	26.92	36.56	99.88	99.14	94.54
WGS	KE-9	Pair-end	250bp	150.52	30.10	37.18	99.88	99.11	94.33
WGS	LJ01	Pair-end	250bp	72.30	14.46	36.88	99.80	98.66	92.75

WGS	LL01_2	Pair-end	250bp	66.46	13.30	38.00	99.77	98.42	91.85
WGS	LL01_3	Pair-end	250bp	90.39	18.08	36.87	99.83	98.88	93.75
WGS	LL04	Pair-end	250bp	86.48	17.30	38.68	99.74	98.30	91.26
WGS	LL05	Pair-end	250bp	87.13	17.42	38.13	99.75	98.36	91.54
WGS	LL06	Pair-end	250bp	88.82	17.76	36.70	99.77	98.51	92.12
WGS	LL09	Pair-end	250bp	92.89	18.58	38.24	99.81	98.78	93.30
WGS	MH01	Pair-end	250bp	83.56	16.72	38.27	99.77	98.47	92.05
WGS	MH03	Pair-end	250bp	87.60	17.52	36.98	99.77	98.41	91.59
WGS	ML01	Pair-end	250bp	87.55	17.52	35.32	99.81	98.78	93.67
WGS	ML01_1	Pair-end	250bp	84.93	16.98	37.33	99.79	98.61	92.48
WGS	ML01_3	Pair-end	250bp	84.21	16.84	36.75	99.77	98.46	91.94
WGS	ML1	Pair-end	250bp	142.91	28.58	38.29	99.86	99.06	94.27
WGS	ML2	Pair-end	250bp	158.11	31.62	36.81	99.86	99.03	94.00
WGS	ML3	Pair-end	250bp	243.38	48.68	36.66	99.86	99.10	94.52
WGS	ML4	Pair-end	250bp	143.29	28.66	36.82	99.87	99.02	93.95
WGS	ML5	Pair-end	250bp	153.49	30.70	37.86	99.86	99.00	93.94
WGS	MS004	Pair-end	250bp	96.95	19.38	37.67	99.79	98.64	92.55
WGS	MS03	Pair-end	250bp	106.28	21.26	38.71	99.80	98.75	93.25

WGS	MZ01	Pair-end	250bp	97.51	19.50	36.96	99.78	98.61	92.61
WGS	NA1	Pair-end	250bp	205.92	41.18	35.02	99.89	99.33	95.72
WGS	NA12	Pair-end	250bp	166.29	33.26	36.63	99.87	99.09	94.33
WGS	NA14	Pair-end	250bp	266.52	53.30	36.48	99.88	99.16	94.86
WGS	NA15	Pair-end	250bp	141.58	28.32	36.48	99.90	99.35	95.84
WGS	NA4	Pair-end	250bp	265.75	53.16	35.95	99.89	99.27	95.35
WGS	NJ01	Pair-end	250bp	89.75	17.96	38.41	99.80	98.71	93.02
WGS	NJ02	Pair-end	250bp	74.82	14.96	36.31	99.80	98.69	92.97
WGS	NJ03	Pair-end	250bp	86.14	17.22	36.84	99.83	98.90	93.98
WGS	NJ04	Pair-end	250bp	78.22	15.64	36.74	99.77	98.48	91.96
WGS	NJ05	Pair-end	250bp	97.36	19.48	38.40	99.74	98.29	91.22
WGS	NJ06	Pair-end	250bp	92.60	18.52	37.18	99.80	98.74	93.07
WGS	NJ07	Pair-end	250bp	109.93	21.98	37.01	99.76	98.43	91.87
WGS	NJ08	Pair-end	250bp	92.15	18.42	38.06	99.74	98.30	91.36
WGS	SC03	Pair-end	250bp	135.32	27.06	37.12	99.82	98.85	93.61
WGS	SC08	Pair-end	250bp	98.49	19.70	37.52	99.79	98.61	92.64
WGS	SC10	Pair-end	250bp	177.69	35.54	37.09	99.82	98.81	93.43
WGS	SC11	Pair-end	250bp	206.00	41.20	37.21	99.77	98.44	91.88

WGS	SC12	Pair-end	250bp	122.24	24.44	37.93	99.77	98.51	92.12
WGS	SC13	Pair-end	250bp	127.80	25.56	37.84	99.78	98.59	92.47
WGS	SC14	Pair-end	250bp	129.51	25.90	37.12	99.78	98.62	92.58
WGS	SC15	Pair-end	250bp	226.83	45.36	37.70	99.82	98.87	93.76
WGS	SC16	Pair-end	250bp	145.50	29.10	39.75	99.76	98.39	91.62
WGS	SC17	Pair-end	250bp	240.67	48.14	37.58	99.78	98.53	92.21
WGS	SC18	Pair-end	250bp	337.81	67.56	37.06	99.77	98.45	91.95
WGS	SC19	Pair-end	250bp	150.28	30.06	38.19	99.86	99.02	93.94
WGS	SC20	Pair-end	250bp	89.41	17.88	37.04	99.83	98.88	93.61
WGS	SC21	Pair-end	250bp	253.68	50.74	39.07	99.85	99.00	94.10
WGS	SC23	Pair-end	250bp	134.61	26.92	39.10	99.86	99.00	93.91
WGS	SC24	Pair-end	250bp	237.15	47.44	39.98	99.84	98.93	93.70
WGS	SC25	Pair-end	250bp	93.63	18.72	36.94	99.81	98.75	93.02
WGS	SC26	Pair-end	250bp	424.67	84.94	38.23	99.78	98.56	92.37
WGS	SC27	Pair-end	250bp	153.56	30.72	39.12	99.79	98.71	93.16
WGS	SC28	Pair-end	250bp	244.21	48.84	38.70	99.78	98.59	92.50
WGS	SC29	Pair-end	250bp	194.38	38.88	36.95	99.77	98.47	91.92
WGS	SC30	Pair-end	250bp	196.85	39.38	36.81	99.83	98.84	93.31

WGS	SC31	Pair-end	250bp	145.33	29.06	37.93	99.88	99.12	94.48
WGS	SC32	Pair-end	250bp	247.68	49.54	37.41	99.85	98.96	93.78
WGS	SC33	Pair-end	250bp	146.57	29.32	37.36	99.85	98.95	93.59
WGS	SC34	Pair-end	250bp	234.21	46.84	37.66	99.83	98.78	92.94
WGS	SC35	Pair-end	250bp	137.33	27.46	38.70	99.86	98.96	93.67
WGS	SC36	Pair-end	250bp	240.06	48.02	37.23	99.83	98.80	92.97
WGS	SC37	Pair-end	250bp	97.13	19.42	37.53	99.75	98.38	91.51
WGS	SC38	Pair-end	250bp	145.12	29.02	37.25	99.84	98.85	93.10
WGS	SC39	Pair-end	250bp	91.83	18.36	37.67	99.75	98.35	91.34
WGS	SC40	Pair-end	250bp	99.95	20.00	37.68	99.75	98.32	91.27
WGS	SC41	Pair-end	250bp	92.02	18.40	39.56	99.78	98.57	92.25
WGS	SC42	Pair-end	250bp	93.68	18.74	38.10	99.77	98.51	92.10
WGS	SC43	Pair-end	250bp	80.89	16.18	37.62	99.75	98.33	91.28
WGS	SC44	Pair-end	250bp	250.58	50.12	37.06	99.84	98.95	93.68
WGS	SC45	Pair-end	250bp	104.01	20.80	37.09	99.81	98.73	92.88
WGS	SC46	Pair-end	250bp	140.42	28.08	37.81	99.85	98.94	93.59
WGS	SC47	Pair-end	250bp	221.08	44.22	36.91	99.85	99.02	94.07
WGS	SC48	Pair-end	250bp	141.86	28.38	38.03	99.88	99.16	94.69

WGS	SC49	Pair-end	250bp	269.68	53.94	38.12	99.86	99.02	94.13
WGS	SC50	Pair-end	250bp	146.79	29.36	39.20	99.85	98.94	93.60
WGS	SC51	Pair-end	250bp	286.84	57.36	37.20	99.84	98.92	93.57
WGS	SC52	Pair-end	250bp	137.45	27.50	37.51	99.86	98.98	93.81
WGS	SC53	Pair-end	250bp	266.34	53.26	39.21	99.85	99.02	94.22
WGS	SC54	Pair-end	250bp	140.09	28.02	38.46	99.86	99.06	94.19
WGS	SC55	Pair-end	250bp	259.09	51.82	38.44	99.85	98.97	93.85
WGS	SC56	Pair-end	250bp	142.28	28.46	38.46	99.88	99.15	94.62
WGS	SC57	Pair-end	250bp	130.97	26.20	35.45	99.90	99.28	95.25
WGS	SC58	Pair-end	250bp	143.13	28.62	40.10	99.87	99.08	94.28
WGS	SC59	Pair-end	250bp	243.84	48.76	39.51	99.84	98.98	93.94
WGS	SC60	Pair-end	250bp	253.39	50.68	38.48	99.84	98.94	93.76
WGS	XS01	Pair-end	250bp	79.53	15.90	38.08	99.76	98.44	91.82
WGS	YA01	Pair-end	250bp	89.97	18.00	39.73	99.80	98.75	93.25
WGS	YJ01	Pair-end	250bp	90.60	18.12	37.19	99.76	98.39	91.53
WGS	YJ02	Pair-end	250bp	97.11	19.42	37.14	99.81	98.73	93.02
WGS	YX01	Pair-end	250bp	87.25	17.44	37.85	99.81	98.81	93.40
WGS	YX02	Pair-end	250bp	107.84	21.56	36.23	99.82	98.88	93.78

WGS	YX03	Pair-end	250bp	101.40	20.28	37.61	99.74	98.30	91.27
WGS	YX04	Pair-end	250bp	85.73	17.14	37.35	99.80	98.71	92.88
WGS	YX05	Pair-end	250bp	96.78	19.36	36.97	99.79	98.60	92.39
WGS	YX06	Pair-end	250bp	70.32	14.06	37.90	99.74	98.25	91.09
WGS	YX07	Pair-end	250bp	89.72	17.94	36.88	99.77	98.53	92.17
WGS	YX09	Pair-end	250bp	97.56	19.52	37.57	99.76	98.44	91.81
WGS	YX10	Pair-end	250bp	93.97	18.80	36.69	99.77	98.45	91.85
WGS	YX11	Pair-end	250bp	85.88	17.18	38.52	99.73	98.26	91.11
WGS	ZT01	Pair-end	250bp	92.27	18.46	38.52	99.77	98.53	92.13
RNA-Seq	SF01	Pair-end	300bp	105.68	21.14	49.72	99.77	98.90	94.44
RNA-Seq	SF02	Pair-end	300bp	97.67	19.54	49.45	99.78	98.82	93.85
RNA-Seq	SF03	Pair-end	300bp	100.82	20.16	50.11	99.78	98.83	94.02
RNA-Seq	SF04	Pair-end	300bp	38.05	7.62	49.78	99.81	99.05	94.82
RNA-Seq	SF05	Pair-end	300bp	99.35	19.88	49.66	99.80	98.97	94.55
RNA-Seq	SF06	Pair-end	300bp	87.07	17.42	50.60	99.78	98.93	94.68
RNA-Seq	SF07	Pair-end	300bp	88.80	17.76	51.31	99.77	98.81	94.12
RNA-Seq	SF08	Pair-end	300bp	131.83	26.36	50.43	99.78	98.95	94.75
RNA-Seq	SF09	Pair-end	300bp	115.65	23.14	49.94	99.77	98.85	94.41

RNA-Seq	SF10	Pair-end	300bp	115.93	23.18	51.38	99.75	98.76	94.04
RNA-Seq	SF11	Pair-end	300bp	113.99	22.80	48.79	99.78	98.87	94.17
RNA-Seq	SF12	Pair-end	300bp	82.44	16.48	49.19	99.80	98.99	94.74
RNA-Seq	SF13	Pair-end	300bp	99.54	19.90	52.23	99.70	98.57	93.35
RNA-Seq	SF14	Pair-end	300bp	129.01	25.80	48.23	99.80	98.94	94.38
RNA-Seq	SF15	Pair-end	300bp	106.16	21.24	48.55	99.80	98.97	94.60
RNA-Seq	SF16	Pair-end	300bp	100.85	20.16	49.16	99.78	98.91	94.45
RNA-Seq	SF17	Pair-end	300bp	104.22	20.84	48.90	99.78	98.92	94.55
RNA-Seq	SF18	Pair-end	300bp	100.14	20.02	50.07	99.77	98.88	94.48
RNA-Seq	SF25	Pair-end	300bp	99.23	19.84	50.05	99.78	98.93	94.66
RNA-Seq	SF26	Pair-end	300bp	94.49	18.90	50.95	99.75	98.79	94.18
RNA-Seq	SF27	Pair-end	300bp	93.05	18.62	48.92	99.79	98.93	94.47
RNA-Seq	SF31	Pair-end	300bp	100.00	20.00	50.31	99.78	98.90	94.48
RNA-Seq	SF32	Pair-end	300bp	96.88	19.38	50.08	99.77	98.89	94.42
RNA-Seq	SF33	Pair-end	300bp	83.92	16.78	50.53	99.77	98.85	94.29
RNA-Seq	SF37	Pair-end	300bp	113.61	22.72	48.84	99.79	98.96	94.66
RNA-Seq	SF38	Pair-end	300bp	114.26	22.86	48.37	99.72	98.79	94.38
RNA-Seq	SF39	Pair-end	300bp	83.03	16.60	49.37	99.80	99.01	94.90

RNA-Seq	SF40	Pair-end	300bp	89.04	17.80	49.22	99.79	98.94	94.56
RNA-Seq	SF41	Pair-end	300bp	87.70	17.54	49.09	99.80	98.98	94.72
RNA-Seq	SF42	Pair-end	300bp	100.27	20.06	48.59	99.78	98.88	94.28
RNA-Seq	SF43	Pair-end	300bp	85.66	17.14	49.27	99.75	98.73	93.85
RNA-Seq	SF44	Pair-end	300bp	81.45	16.30	49.35	99.80	99.06	95.10
RNA-Seq	SF45	Pair-end	300bp	71.40	14.28	49.05	99.79	98.94	94.43
RNA-Seq	SF46	Pair-end	300bp	112.82	22.56	49.46	99.77	98.86	94.25
RNA-Seq	SF47	Pair-end	300bp	100.54	20.10	48.72	99.79	98.91	94.38
RNA-Seq	SF48	Pair-end	300bp	96.40	19.28	48.43	99.80	98.97	94.55
RNA-Seq	SF49	Pair-end	300bp	90.64	18.12	48.77	99.79	98.96	94.59
RNA-Seq	SF50	Pair-end	300bp	91.42	18.28	49.59	99.75	98.74	93.87
RNA-Seq	SF51	Pair-end	300bp	95.87	19.18	49.89	99.76	98.81	94.18
RNA-Seq	SF52	Pair-end	300bp	86.78	17.36	49.43	99.78	98.90	94.34
RNA-Seq	SF53	Pair-end	300bp	108.57	21.72	49.42	99.77	98.79	93.72
RNA-Seq	SF54	Pair-end	300bp	63.91	12.78	49.31	99.80	99.02	94.86
RNA-Seq	SF55	Pair-end	300bp	65.16	13.04	49.32	99.80	98.97	94.71
RNA-Seq	SF56	Pair-end	300bp	62.07	12.42	48.68	99.80	99.02	94.92
RNA-Seq	SF57	Pair-end	300bp	59.45	11.90	49.47	99.78	98.87	94.16

RNA-Seq	SF58	Pair-end	300bp	81.90	16.38	48.82	99.80	98.96	94.43
RNA-Seq	SF59	Pair-end	300bp	83.92	16.78	48.84	99.80	99.01	94.70
RNA-Seq	SF60	Pair-end	300bp	90.08	18.02	48.84	99.79	98.88	94.05
RNA-Seq	SF61	Pair-end	300bp	76.30	15.26	48.93	99.79	98.93	94.41
RNA-Seq	SF62	Pair-end	300bp	78.68	15.74	48.78	99.77	98.77	93.72
RNA-Seq	SF63	Pair-end	300bp	71.15	14.22	48.52	99.79	98.92	94.42
RNA-Seq	SF64	Pair-end	300bp	92.93	18.58	49.33	99.80	98.98	94.75
RNA-Seq	SF65	Pair-end	300bp	52.64	10.52	49.65	99.74	98.66	93.35
RNA-Seq	SF66	Pair-end	300bp	82.67	16.54	49.72	99.75	98.73	93.66
RNA-Seq	SF67	Pair-end	300bp	86.74	17.34	50.04	99.76	98.77	93.75
RNA-Seq	SF68	Pair-end	300bp	95.15	19.04	49.05	99.80	98.92	94.38
RNA-Seq	SF69	Pair-end	300bp	105.82	21.16	49.24	99.80	98.98	94.59
RNA-Seq	SF70	Pair-end	300bp	104.70	20.94	48.88	99.78	98.82	94.02
RNA-Seq	SF71	Pair-end	300bp	91.51	18.30	49.89	99.77	98.88	94.45
RNA-Seq	SF72	Pair-end	300bp	74.63	14.92	49.48	99.78	98.82	93.90
RNA-Seq	SF73	Pair-end	300bp	77.38	15.48	51.52	99.79	98.96	94.57
RNA-Seq	SF74	Pair-end	300bp	63.55	12.72	50.25	99.75	98.66	93.31
RNA-Seq	SF75	Pair-end	300bp	47.54	9.50	48.14	99.76	98.70	93.29

RNA-Seq	SF76	Pair-end	300bp	104.06	20.82	48.46	99.82	99.04	94.97
RNA-Seq	SF77	Pair-end	300bp	93.79	18.76	48.49	99.80	99.05	95.05
RNA-Seq	SF78	Pair-end	300bp	90.56	18.12	49.32	99.76	98.75	93.65
RNA-Seq	SF82	Pair-end	300bp	90.71	18.14	50.14	99.79	98.85	93.96
RNA-Seq	SF83	Pair-end	300bp	106.86	21.38	50.43	99.76	98.76	93.72
RNA-Seq	SF84	Pair-end	300bp	90.70	18.14	49.20	99.80	98.94	94.38
RNA-Seq	SF85	Pair-end	300bp	96.22	19.24	49.86	99.78	98.90	94.38
RNA-Seq	SF86	Pair-end	300bp	101.48	20.30	48.37	99.78	98.94	94.63
RNA-Seq	SF87	Pair-end	300bp	93.65	18.74	49.89	99.79	98.95	94.61

Table S2 | The complete BUSCO gene result of the assembly.

Species	Complete BUSCO genes	Complete and single-copy BUSCO genes	Complete and duplicated BUSCO genes	Fragmented BUSCO genes	Missing BUSCO genes
Drosophila melanogaster	99.70%	99.00%	0.70%	0.20%	0.10%
Plutella xylostella	87.90%	72.60%	15.30%	2.60%	9.50%
Manduca sexta	96.70%	94.00%	2.70%	1.70%	1.60%
Bombyx mori	95.60%	95.10%	0.50%	2.20%	2.20%
Danaus plexippus	98.00%	95.90%	2.10%	1.00%	1.00%
Helicoverpa armigera	96.70%	95.60%	1.10%	1.40%	1.90%
Spodoptera litura	97.20%	95.80%	1.40%	1.00%	1.80%
**SFynMstLFR**	95.00%	85.20%	9.80%	2.80%	2.20%
**PRJNA380964**	91.10%	74.00%	17.10%	2.70%	6.20%
**PRJNA257248**	84.50%	83.40%	1.10%	6.60%	8.90%
**PRJEB13110**	88.20%	76.70%	11.50%	7.70%	4.10%
**PRJNA344686**	86.00%	81.00%	5.00%	10.50%	3.50%

Note: The lineage dataset is: insecta_odb9 (Creation date: 2016-10-21, number of species: 42, number of BUSCOs: 1658). SFynMstLFR, PRJNA380964, PRJNA257248, PRJEB13110, PRJNA344686 were different versions of the genome of *Spodoptera frugiperda*, the detailed information may find in the NCBI according to the provided number.

Table S3 | Mapping reads against the assembled genome using raw reads generated by different libraries.

Type	HIC	stLFR	RNA-seq	WGS
**Total Mapped Reads**	93.58%	95.60%	98.98%	90.71%
**Genome Coverage**	97.70%	99.90%	--	90.80%

Table S4 The sequencing depth, mapping rate and coverage of the 163 resequencing samples.

Samples	Depth (×)	Mapping rate	Coverage
KE-1	41.12	97.49%	98.90%
KE-10	28.07	97.08%	98.90%
KE-3	25.58	97.02%	98.90%
KE-5	32.71	97.13%	98.80%
KE-4	31.69	97.44%	98.70%
KE-8	28.68	96.69%	98.70%
KE-9	36.25	97.35%	98.70%
KE-7	31.11	97.26%	98.00%
KE-6	31.11	97.82%	97.60%
NA4	31.1	96.96%	93.10%
SC26	43.91	97.25%	93.00%
NA14	48.18	97.84%	92.60%
SC18	41.4	97.34%	92.60%
SC10	44.41	97.54%	92.50%
NA15	49.91	97.75%	92.40%
SC24	52.04	97.67%	92.20%
SC32	46.2	98.05%	92.20%
SC17	45.81	97.85%	92.10%
SC21	46.46	97.86%	92.00%
SC15	28.01	96.61%	91.90%
SC49	44.91	97.79%	91.80%
GX03	44.24	97.60%	91.70%
SC44	28.53	97.06%	91.70%
SC28	48.9	97.60%	91.60%
SC34	49.9	97.45%	91.60%
ML3	47.25	97.91%	91.50%
SC30	48.1	97.45%	91.50%

SC36	84.93	97.31%	91.40%
SC46	85.34	97.16%	91.40%
SC47	45.31	97.56%	91.40%
NA1	48.68	97.46%	91.30%
SC11	80.18	97.26%	91.30%
SC29	79.87	97.55%	91.30%
ML5	89.49	97.06%	91.20%
NA12	84.14	97.25%	91.20%
SC03	83.32	97.28%	91.20%
SC23	46.88	97.40%	91.20%
SC35	75.12	97.07%	91.10%
ML2	53.3	97.47%	91.00%
ML4	83.32	97.20%	91.00%
SC14	24.81	97.88%	90.80%
SC31	36.8	97.05%	90.80%
SC50	39.62	96.89%	90.80%
D4	45.37	97.24%	90.70%
ML1	36	97.80%	90.70%
SC16	29.06	97.40%	90.70%
SC19	26.45	85.48%	90.70%
SC33	25.74	96.39%	90.70%
SC38	32.36	88.44%	90.70%
SC13	45.68	87.18%	90.60%
SC08	43.6	88.20%	90.50%
SC12	31.14	96.98%	90.50%
SC27	53.9	94.69%	90.50%
SC48	48.34	95.75%	90.50%
MS03	28.44	96.38%	90.40%
NJ07	47.16	93.03%	90.40%
SC45	46.92	97.19%	90.40%
SC60	44.75	81.42%	90.40%

YX05	31.73	97.92%	90.40%
C9	30.21	96.45%	90.30%
D6	36.31	96.58%	90.30%
GX01	29.53	98.06%	90.30%
GX02	27.14	89.84%	90.30%
SC55	50.65	96.63%	90.30%
YX02	24.31	97.23%	90.30%
D1	25.63	88.98%	90.20%
LL05	28.89	95.70%	90.20%
LL06	28.32	87.71%	90.20%
LL09	34.45	91.86%	90.20%
MS04	28.43	96.81%	90.20%
SC53	45.36	96.91%	90.20%
YJ02	28.12	95.79%	90.20%
YX03	45.22	90.95%	90.20%
YX10	53.24	96.65%	90.20%
BS02	82.05	96.67%	90.10%
FJ01_3	30.46	97.23%	90.10%
JC01	48.34	97.46%	90.10%
LL01_3	50.03	94.10%	90.10%
ML01_3	32.31	96.24%	90.10%
MZ01	32.71	95.92%	90.10%
NJ05	68.18	94.91%	90.10%
SC39	88.52	94.63%	90.10%
SC51	30.14	97.31%	90.10%
ZT01	26.52	97.77%	90.10%
C7	22.02	95.12%	90.00%
GX04	89.9	96.37%	90.00%
NJ06	36.73	95.86%	90.00%
NJ08	43.41	86.75%	90.00%
YA01	30.31	96.57%	90.00%

YX01	25.22	96.78%	90.00%
YX09	29.14	96.12%	90.00%
D10	32.03	96.71%	89.90%
MH01	33.18	97.88%	89.90%
MH03	43.26	97.29%	89.90%
NJ01	31.46	97.82%	89.90%
SC20	30.91	96.10%	89.90%
SC37	45.29	96.37%	89.90%
SC40	79.46	95.10%	89.90%
SC41	32.79	95.77%	89.90%
YX06	58.91	95.42%	89.90%
BS01	69.78	97.39%	89.80%
FAW5.2	40.95	96.50%	89.80%
ML01_1	43.79	97.21%	89.80%
SC25	43.42	85.76%	89.80%
SC42	111.9	95.04%	89.80%
YX04	29.78	96.04%	89.80%
YX07	77.87	88.03%	89.80%
FJ01_1	43.14	91.95%	89.70%
FJ01_2	64.85	78.93%	89.70%
LL04	30.98	95.48%	89.70%
NJ02	51.9	96.93%	89.70%
NJ03	77.15	97.22%	89.70%
XS01	78.76	93.02%	89.70%
YJ01	46.77	89.62%	89.70%
YX11	65.65	97.35%	89.70%
D3	55.29	95.19%	89.60%
A10	81.72	88.21%	89.50%
ML01	135.13	91.56%	89.50%
NJ04	79.35	97.41%	89.50%
C3	49.17	97.18%	89.40%

LJ01	32.47	96.25%	89.40%
LL01_2	48.21	95.53%	89.40%
SC43	29.23	90.18%	89.40%
SC52	30.45	95.39%	89.40%
A5	80.85	97.27%	89.30%
C4	49.3	96.88%	89.30%
D2	83.61	97.35%	89.30%
SC59	45.86	95.91%	89.30%
C1	32.73	97.13%	89.10%
C2	33.95	97.34%	89.10%
D9	30.93	96.76%	89.00%
SC54	43.16	88.59%	89.00%
SC56	75.11	97.76%	89.00%
A3	46.77	94.42%	88.90%
A4	34.55	95.79%	88.90%
A9	90.89	96.23%	88.90%
D8	84.1	96.51%	88.90%
SC57	46.57	97.43%	88.90%
A1	66.51	97.31%	88.80%
A7	47.63	97.69%	88.80%
A8	48.68	97.55%	88.80%
C8	47.04	91.89%	88.80%
D7	97.24	97.66%	88.80%
D5	48.64	97.92%	88.70%
A6	44.28	97.28%	88.60%
C6	88.72	98.08%	88.60%
FAW1.2	86.42	97.84%	88.50%
SC58	83.69	98.23%	88.50%
A2	26.42	95.84%	88.30%
FAW1.1	26.84	86.15%	88.20%
B8	29.93	95.65%	87.90%

B5	29.72	98.01%	87.50%
C10	25.92	92.30%	87.50%
B1	30.38	97.54%	87.10%
B3	34.2	96.89%	87.00%
B4	28.14	95.04%	87.00%
B6	32.44	96.77%	86.90%
B2	23.63	96.39%	86.80%
B7	33	97.41%	86.80%
B9	31.46	95.83%	86.80%
B10	36.34	96.03%	86.70%
FAW2.2	28.45	95.24%	86.10%
FAW2.1	31.21	97.54%	85.30%

Table S5 | K-mer based genome survey results calculated by the GCE software.

Samples	genome size(Mb)	heterozygous ratio	repeat ratio
SC24	977.561341	0.003828871	0.243287
SC21	874.340065	0.004748888	0.246687
SC28	846.217305	0.002750735	0.659175
SC26	836.619120	0.002073576	0.521523
SC17	797.069980	0.002009882	0.533027
SC49	796.109551	0.002898735	0.659732
SC15	793.086128	0.001838276	0.561400
SC44	784.693709	0.001839076	0.543139
D1	779.720054	0.000952971	0.562769
SC11	779.226146	0.001281806	0.552523
SC36	775.109434	0.001384018	0.542359
SC34	774.324944	0.003349682	0.552095
D6	774.068006	0.000961647	0.558824
NA14	772.211736	0.001705200	0.564824
SC47	771.849088	0.000575254	0.545093
SC29	768.187461	0.001068071	0.560577
ML3	766.376712	0.001452841	0.550400
SC30	765.870748	0.000987882	0.536488
SC32	764.921516	0.001857224	0.645969
NA4	753.837788	0.003337235	0.329505
SC60	745.146173	0.004698047	0.574464
NA1	702.703204	0.004992947	0.287906
SC59	644.193421	0.002737171	0.683969
SC53	545.005925	0.004319629	0.532412
SC55	536.940269	0.003117041	0.569925
D10	515.866395	0.001622976	0.315265
D8	515.189284	0.017778529	0.315084
C9	513.616427	0.001496035	0.304131
SC51	512.814357	0.001674947	0.334524
C7	512.661931	0.001642076	0.322977
D3	511.411040	0.018642588	0.548606
D2	510.283645	0.000390887	0.540588

Note: 32 individuals with the sequencing depth larger than 60× were selected for this genome survey analysis.

Table S6 | Genome coverage evaluated by expressed sequence tags (EST) and transcriptome datasets.

	Dataset	Number	Total length (bp)	Base covered by assembly(%)	Sequences covered by assembly(%)	with >90% sequence in one scaffold		with >50% sequence in one scaffold	
						Number	Percent(%)	Number	Percent(%)
**EST**	Sf_cDNA								
	>0bp	48,901	29,207,093	94.82	98.82	37,980	77.67	47,874	97.90
	>200bp	48,215	29,097,650	94.85	98.85	37,695	78.18	47,212	97.92
	>500bp	35,561	24,232,582	95.35	98.89	30,557	85.93	34,761	97.75
	sf21_raw_trinity_cdhit90								
	>0bp	20,691	22,620,514	94.15	96.86	17,656	85.33	19,250	93.04
	>200bp	20,691	22,620,514	94.15	96.86	17,656	85.33	19,250	93.04
	>500bp	11,695	19,681,612	94.57	97.93	10,211	87.31	10,975	93.84
	>1000bp	7,208	16,487,451	95.13	98.68	6,420	89.07	6,845	94.96
**RNA-seq**	SFgdRNA_1								
	>0bp	51,298	46,277,886	87.30	93.34	36,718	71.58	43,498	84.79

>200bp	51,298	46,277,886	87.30	93.34	36,718	71.58	43,498	84.79
>500bp	26,701	38,482,330	88.28	96.97	19,300	72.28	23,269	87.15
>1000bp	14,006	29,521,540	89.29	98.80	10,239	73.10	12,580	89.82
SFgdRNA_2								
>0bp	53,603	61,239,312	85.14	92.45	34,867	65.05	43,135	80.47
>200bp	53,603	61,239,312	85.14	92.45	34,867	65.05	43,135	80.47
>500bp	31,233	54,166,519	86.11	96.62	20,646	66.10	25,986	83.20
>1000bp	19,067	45,562,462	87.33	98.33	13,041	68.40	16,468	86.37

Table S7 | The result of gene prediction for the *de novo* prediction with Augustus, glimmerHMM and SNAP, and homology based on *Bombyx mori*, *Danaus plexippus*, *Drosophila melanogaster* and *Spodoptera litura* genomes.

	GeneSet	Total number of gene	Average of mRNA_length	Average of cds_length	Average of exon_number	Average of exon_length	Average of intron_numebr	Average of intron_length
De novo	augustus	18,689	7399.29	1357.20	6.27	216.59	5.27	1147.32
	glimmerHMM	55,551	8016.99	815.92	4.46	182.77	3.36	2078.67
	snap	35,165	23222.51	1216.98	7.75	156.97	6.75	3258.74
**Homolog**	*Bombyx mori*	27,441	2993.05	865.16	3.50	247.44	2.50	852.37
	Danaus plexippus	22,037	4550.76	1067.59	4.21	253.80	3.21	1086.33
	Drosophila melanogaster	11,700	4487.43	952.76	4.13	230.42	3.13	1127.53
	Spodoptera litura	23,580	5806.58	1337.13	4.90	272.83	3.9	1145.74
**RNA-seq**	stringtie	30,470	3746.13	509.73	2.84	179.57	1.84	1758.23
**Glean**		21,377	5029.31	1363.54	5.49	248.17	4.49	815.64
**Check**		22,201	5309.11	1346.57	5.46	246.61	4.46	888.39

Table S8 | Statistics on functional annotation of the fall armyworm predicted genes.

	Number	Percentage
**Total**	22,201	100.00%
**Swissprot**	14,724	66.32%
**KEGG**	16,072	72.39%
**COG**	6,365	28.67%
**TrEMBL**	20,678	93.14%
**Interpro**	14,799	66.66%
**Overall**	20,754	93.48%

Table S9 | Assessment of gene set in BUSCO evaluation.

Species	Complete BUSCOs	Complete and single-copy BUSCOs	Complete and duplicated BUSCOs	Fragmented BUSCOs	Missing BUSCOs
Drosophila melanogaster	97.90%	97.10%	0.80%	0.20%	1.90%
Plutella xylostella	74.90%	62.80%	12.10%	5.60%	19.50%
Manduca sexta	93.40%	90.30%	3.10%	3.30%	3.30%
Bombyx mori	89.90%	89.40%	0.50%	6.70%	3.40%
Danaus plexippus	97.00%	95.40%	1.60%	3.00%	0.00%
Helicoverpa armigera	95.30%	94.50%	0.80%	2.60%	2.10%
Spodoptera litura	98.70%	97.30%	1.40%	0.70%	0.60%
Spodoptera frugiperda	94.20%	84.10%	10.10%	3.90%	1.90%

Note: The lineage dataset is: insecta_odb9 (Creation date: 2016-10-21, number of species: 42, number of BUSCOs: 1658).

Table S10 | Enriched IPR items in PSGs.

IPR_ID	IPR_Title	Q-value	Number of DEGs	Number of all genes
**IPR000477**	Reverse transcriptase domain	7.68E-78	57	664
**IPR005135**	Endonuclease/exonuclease/phosphatase	6.57E-23	32	261
**IPR001878**	Zinc finger, CCHC-type	2.90E-07	19	339
**IPR003094**	Fructose-2,6-bisphosphatase	0.013608245	2	3
**IPR006900**	Sec23/Sec24, helical domain	0.018527094	2	4
**IPR000330**	SNF2-related	0.018527094	3	17

Table S11 | Gene numbers of 11 gene families that scanned by HMMER3.

	ABC	*CES*	*CYP*	*GST*	*UGT*	*AOX*	*CHIA*	*EPIA*	*PIF*	*PTCHD*	*PTP*
Bombyx mori	60	135	126	25	50	15	18	8	24	5	22
Danaus plexippus	92	72	125	52	57	6	35	4	2	15	40
Drosophila melanogaster	50	49	81	20	39	11	15	9	0	8	16
Helicoverpa armigera	54	97	114	29	46	15	13	9	10	6	14
Manduca sexta	78	120	143	29	53	30	21	17	0	14	34
Plutella xylostella	127	79	103	24	34	10	21	11	0	5	18
Spodoptera frugiperda	76	78	425	48	36	18	13	9	79	5	14
Spodoptera litura	132	141	143	44	65	22	21	8	27	11	19

Table S12 | 283 *CYP* genes that specific to *Spodoptera frugiperda*.

Gene ID	Gene ID	Gene ID	Gene ID	Gene ID	Gene ID	Gene ID	Gene ID
SFR00072	SFR03105	SFR01325	SFR16205	SFR04688	SFR18386	SFR18073	SFR22042
SFR00134	SFR08744	SFR10442	SFR16224	SFR04726	SFR20588	SFR02695	SFR16252
SFR00173	SFR08805	SFR10481	SFR18719	SFR20048	SFR04407	SFR19331	SFR16387
SFR00174	SFR16009	SFR14393	SFR08256	SFR20050	SFR06725	SFR03333	SFR04898
SFR00186	SFR16974	SFR17174	SFR08867	SFR03269	SFR21631	SFR14560	SFR03758
SFR00266	SFR17241	SFR17175	SFR08871	SFR03276	SFR09600	SFR21029	SFR04567
SFR00281	SFR06006	SFR00810	SFR12773	SFR10100	SFR17977	SFR04284	SFR20982
SFR00288	SFR06025	SFR00813	SFR15530	SFR11981	SFR02738	SFR20767	SFR02099
SFR01118	SFR07672	SFR01087	SFR18871	SFR08049	SFR02921	SFR22060	SFR21522
SFR07389	SFR09009	SFR01210	SFR19006	SFR13801	SFR08653	SFR17827	SFR03240
SFR07457	SFR09020	SFR04044	SFR19028	SFR13871	SFR09637	SFR03601	SFR03612
SFR07489	SFR09024	SFR04675	SFR00463	SFR03565	SFR10785	SFR14572	SFR00829
SFR07532	SFR18465	SFR04680	SFR00467	SFR04272	SFR10797	SFR16437	SFR04421
SFR07540	SFR00792	SFR10856	SFR00644	SFR04275	SFR01135	SFR20032	SFR06165
SFR07553	SFR05788	SFR10866	SFR00649	SFR05833	SFR02766	SFR02961	SFR19913
SFR13308	SFR09757	SFR11683	SFR17306	SFR10876	SFR16281	SFR21423	SFR04422
SFR20161	SFR09766	SFR17967	SFR02181	SFR12751	SFR16562	SFR08308	SFR06448
SFR01253	SFR18593	SFR10651	SFR03389	SFR12914	SFR16563	SFR19891	SFR06550
SFR03301	SFR18594	SFR15059	SFR08097	SFR14512	SFR20095	SFR04494	SFR16689
SFR05156	SFR08451	SFR15076	SFR08143	SFR01418	SFR05480	SFR05610	SFR17524
SFR07814	SFR10425	SFR16054	SFR09240	SFR01420	SFR20025	SFR19417	SFR18102
SFR07820	SFR13922	SFR18278	SFR09413	SFR01421	SFR03495	SFR14585	SFR17393
SFR12059	SFR16254	SFR08600	SFR09535	SFR05113	SFR11492	SFR16881	SFR18037
SFR12515	SFR20311	SFR09485	SFR10619	SFR05115	SFR18929	SFR18260	SFR19768
SFR15604	SFR03783	SFR09487	SFR13116	SFR06329	SFR02956	SFR06504	SFR04086
SFR02344	SFR06619	SFR09489	SFR05368	SFR06386	SFR03061	SFR18943	SFR02717
SFR03438	SFR13490	SFR09498	SFR14715	SFR08165	SFR03232	SFR13442	SFR08668
SFR03458	SFR17574	SFR09552	SFR16832	SFR09045	SFR21388	SFR13443	SFR07780
SFR07273	SFR17683	SFR09554	SFR01469	SFR10215	SFR22093	SFR08400	SFR01357
SFR08202	SFR17690	SFR09567	SFR11696	SFR10226	SFR03425	SFR12652	SFR04341
SFR08203	SFR02678	SFR18294	SFR17118	SFR12077	SFR21123	SFR14625	SFR04411
SFR10245	SFR06359	SFR04141	SFR11307	SFR12081	SFR15143	SFR19895	
SFR13063	SFR12604	SFR05273	SFR11359	SFR21441	SFR01835	SFR11913	
SFR15489	SFR12646	SFR07206	SFR11417	SFR03472	SFR02389	SFR08357	
SFR19490	SFR18255	SFR16200	SFR02847	SFR12134	SFR16850	SFR02912	
SFR02516	SFR19143	SFR16204	SFR04687	SFR15455	SFR11867	SFR04214	

Table S13 | The statistic of the global nucleotide diversity (π) in nine population.

Group	π
**America_A**	0.0025923
**America_B**	0.0010229
**America_C**	0.0019534
**America_D**	0.0017428
**Ethiopia**	0.0023010
**Kenya**	0.0035087
**Guangxi**	0.0022234
**South_Africa**	0.0017768
**Yunnan**	0.0021849

Table S14 | The F_{ST} estimate within nine population.

F_{ST}	America B	America C	America D	Ethiopia	Kenya	Guangxi	South Africa	Yunnan
**America A**	0.33795	0.17256	0.23152	0.1071	0.1483	0.1313	0.1286	0.1321
**America B**		0.4064	0.50295	0.3737	0.3931	0.3189	0.4779	0.3264
**America C**			0.1409	0.2036	0.2368	0.2004	0.256	0.2056
**America D**				0.2671	0.2925	0.2506	0.3358	0.2561
**Ethiopia**					0.0383	0.0111	0.0544	0.0106
**Kenya**						0.0274	0.1122	0.0234
**Guangxi**							0.0525	0.0079
**South Africa**								0.0524

Note: 0-0.05 0.05-0.15 0.15-0.25 >0.25

Table S15 | The statistics of selective sweep signal and harbor gene number.

	Selective sweep signal	harbor gene
**Ethiopia**	4450	233
**Kenya**	4526	293
**South Africa**	4221	186
**Guangxi**	4391	246
**Yunnan**	4327	339
**America A**	4501	288
**America B**	3921	110
**America C**	4383	322
**America D**	4288	196

Table S16 | The 23 pesticide treatments and one control (Biological sample duplication: 3).

Pesticide code	Pesticide Types	Pesticides treatment	Treated concentration	Differentially expressed genes	Down regulated	Up regulated	Targets
**P1**	biological	80 Billion spores/ml Metarhizium anisopliae suspension concentrate	60ul/L	1184	447	737	Nervous System
**P2**	biological	100 Billion spores/g Bacillus thuringiensis suspension concentrate	27ul/L	2055	824	1231	Digestive System*Nervous System
**P3**	biological	100 Billion spores/ml Empedobacter brevis suspension concentrate	50ul/L	752	345	407	Digestive System
**P4**	biological	0.3% Azadirachtin emulsifiable concentrate	20ul/L	1657	579	1078	Digestive System
**P5**	Single Chemical	3% Emamectin Benzoate microemulsion	8ul/L	1905	969	936	Nervous System
**P6**	Single Chemical	15% Indoxacarb suspension concentrate	28ul/L	1450	757	693	Nervous System
**P7**	Single Chemical	19% Spinetoram water dispersible granule	2.2ul/L	2341	968	1373	Nervous System
**P8**	Single Chemical	10% Chlorfenapyr suspension concentrate	40ul/L	2458	1023	1435	Energy Metabolism
**P9**	Single Chemical	10% Ethofenprox suspension concentrate	80ul/L	1805	800	1005	Nervous System
**P10**	Single Chemical	20% Dinotefuran soluble granules	3.2mg/L	2133	853	1280	Nervous System

**P11**	Mixture of Chemical	25% Cyhalodiamide*Clothianidin suspension concentrate	50ul/L	1807	738	1069	Nervous System
**P12**	Mixture of Chemical	8% Avermectin*Indoxacarb water dispersible granule	3.6mg/L	875	476	399	Nervous System
**P13**	Mixture of Chemical	30% Hexaflumuron*Indoxacarb suspension concentrate	25ul/L	278	168	110	Inhibit insect peeling*Nervous System
**P14**	Mixture of Chemical	33g/L Avermectin*Bifenthrin emulsifiable concentrate	40ul/L	747	357	390	Nervous System
**P15**	Mixture of Chemical	34% Spinetoram*Methoxyfenozide suspension concentrate	30ul/L	547	271	276	Nervous System*Inhibit insect peeling
**P16**	Mixture of Chemical	40% Bifenthrin*Thiacloprid suspension concentrate	30ul/L	886	444	442	Nervous System
**P17**	Mixture of Chemical	10% Emamectin*Indoxacarb suspension concentrate	30ul/L	1880	662	1218	Nervous System
**P18**	Mixture of Chemical	12% Emamectin*Flutolanil microemulsion	25ul/L	1842	558	1284	Nervous System*Ryanodine Receptor
**P19**	Mixture of Chemical	40% Chlorantraniliprole*Thiamethoxam water dispersible granule	2mg/L	2159	960	1199	Ryanodine Receptor*Nervous System

**P20**	Single Chemical	50g/L Lufenuron emulsifiable concentrate	64ul/L	1778	738	1040	Inhibit insect peeling
**P21**	Single Chemical	10% Cyantraniliprole suspension concentrate	64ul/L	716	333	383	Ryanodine Receptor
**P22**	Single Chemical	240g/L Metaflumizone suspension concentrate	160ul/L	1758	683	1075	Nervous System
**P23**	Single Chemical	35% Chlorantraniliprole water dispersible granule	2mg/L	1290	541	749	Ryanodine Receptor
**CK**			control				

Table S17 | The KEGG enrichment of up regulated and down regulated DEGs of 23 pesticide treatments.

	KEGG_ID	No. of Pesticides
up	map00073 Cutin, suberine and wax biosynthesis	19
	map04146 Peroxisome	16
	map03008 Ribosome biogenesis in eukaryotes	16
	map04212 Longevity regulating pathway - worm	14
	map00900 Terpenoid backbone biosynthesis	8
down	map00511 Other glycan degradation	12
	map00051 Fructose and mannose metabolism	11
	map00520 Amino sugar and nucleotide sugar metabolism	11
	map00604 Glycosphingolipid biosynthesis - ganglio series	10
	map01051 Biosynthesis of ansamycins	9
	map00531 Glycosaminoglycan degradation	9
	map00052 Galactose metabolism	8
	map00030 Pentose phosphate pathway	7
	map00040 Pentose and glucuronate interconversions	7
	map00680 Methane metabolism	7
	map00232 Caffeine metabolism	6
	map00603 Glycosphingolipid biosynthesis - globo and isoglobo series	6
	map04142 Lysosome	6

Table S18 | Fold changes of 107 *CYP* genes after 23 pesticide treatments.

gene	k	gene name	1	2	3	4	5	6	7	8	9	10	11	12	13	14	15	16	17	18	19	20	21	22	23
**SFR03129**	K17855	*CYP*2L	0.0	0.0	0.0	0.0	-3.2	-4.1	-5.1	-3.2	-2.8	0.0	-3.8	0.0	0.0	0.0	0.0	0.0	1.2	0.0	0.0	0.0	0.0	1.3	0.0
**SFR13174**	K14999	*CYP*6	0.0	0.0	0.0	-2.2	0.0	0.0	0.0	-1.8	0.0	-1.7	0.0	0.0	0.0	0.0	0.0	0.0	0.0	-1.6	-1.8	-2.1	0.0	-2.0	-2.0
**SFR13175**	K15003	*CYP*9	0.0	0.0	0.0	-1.7	0.0	0.0	0.0	0.0	0.0	0.0	0.0	0.0	0.0	0.0	0.0	0.0	0.0	0.0	0.0	0.0	0.0	0.0	0.0
**SFR13176**	K15003	*CYP* 9	0.0	0.0	0.0	-2.6	0.0	-2.0	0.0	-2.7	-2.4	-1.9	-2.3	0.0	0.0	0.0	0.0	0.0	-1.9	-2.4	-2.2	-2.8	-1.9	-2.5	-2.6
**SFR13178**	K15003	*CYP* 9	0.0	0.0	0.0	-2.7	0.0	0.0	-2.3	-2.2	-2.6	0.0	-2.4	-2.5	0.0	0.0	0.0	0.0	-1.6	-2.7	-2.4	-2.8	-3.0	-2.3	-2.9
**SFR16591**	K14999	*CYP* 6	0.0	0.0	0.0	-1.9	0.0	0.0	0.0	0.0	0.0	0.0	0.0	0.0	0.0	0.0	0.0	0.0	0.0	-2.1	-1.9	0.0	-2.2	0.0	-2.5
**SFR16592**	K14999	*CYP* 6	0.0	0.0	0.0	0.0	0.0	0.0	0.0	0.0	0.0	0.0	0.0	0.0	0.0	0.0	0.0	0.0	0.0	-2.0	0.0	0.0	0.0	0.0	0.0
**SFR16593**	K15003	*CYP* 9	0.0	0.0	0.0	-1.7	0.0	0.0	0.0	0.0	0.0	0.0	0.0	0.0	0.0	0.0	0.0	0.0	0.0	0.0	0.0	-2.1	0.0	-2.0	0.0
**SFR16594**	K15003	*CYP* 9	0.0	0.0	0.0	0.0	0.0	0.0	0.0	0.0	0.0	0.0	0.0	0.0	0.0	0.0	0.0	0.0	0.0	0.0	0.0	-2.2	0.0	-1.9	0.0
**SFR16595**	K15003	*CYP* 9	0.0	0.0	0.0	-2.9	0.0	0.0	-2.7	-2.4	-2.7	0.0	-2.7	-2.8	0.0	0.0	0.0	0.0	-1.7	-2.7	-3.1	-3.2	-3.4	-2.5	-3.3
**SFR15489**	K00665	FASN	0.0	0.0	0.0	0.0	0.0	0.0	0.0	0.0	-2.3	0.0	0.0	0.0	0.0	0.0	0.0	0.0	0.0	0.0	0.0	0.0	0.0	0.0	0.0
**SFR20447**	K14999	*CYP* 6	4.7	0.0	2.5	0.0	0.0	0.0	0.0	0.0	0.0	0.0	0.0	0.0	0.0	0.0	0.0	0.0	0.0	0.0	0.0	0.0	0.0	0.0	0.0
**SFR08808**	K17960	*CYP* 49A	0.0	0.0	0.0	-1.3	0.0	0.0	0.0	0.0	0.0	-1.6	0.0	0.0	0.0	-1.5	0.0	0.0	-1.2	-1.3	-1.5	-1.9	-1.5	-1.6	0.0
**SFR15985**	K08145	SLC2A8	0.0	0.0	0.0	0.0	0.0	1.5	1.7	1.8	0.0	0.0	0.0	0.0	0.0	0.0	0.0	0.0	0.0	0.0	0.0	0.0	0.0	0.0	0.0
**SFR04210**	K15004	*CYP* 12	0.0	0.0	0.0	0.0	0.0	0.0	0.0	0.0	0.0	0.0	0.0	0.0	0.0	0.0	0.0	0.0	0.0	0.0	0.0	-1.9	0.0	-1.9	0.0
**SFR04211**	K15004	*CYP* 12	0.0	0.0	0.0	0.0	0.0	0.0	0.0	0.0	0.0	0.0	0.0	0.0	0.0	0.0	0.0	0.0	0.0	0.0	0.0	0.0	0.0	0.0	0.0
**SFR02217**	K15003	*CYP* 9	0.0	0.0	0.0	-3.1	0.0	0.0	0.0	-2.5	-2.4	-2.1	-2.3	-2.9	0.0	0.0	0.0	0.0	-2.2	-3.1	-3.3	-3.1	-3.2	-2.8	-3.2
**SFR07700**	K14999	*CYP* 6	0.0	0.0	0.0	0.0	0.0	0.0	0.0	0.0	0.0	0.0	0.0	0.0	0.0	0.0	0.0	0.0	0.0	-1.7	0.0	-1.6	0.0	0.0	0.0
**SFR09758**	K14999	*CYP* 6	4.0	0.0	0.0	0.0	4.5	0.0	0.0	0.0	0.0	0.0	0.0	0.0	0.0	0.0	0.0	4.0	0.0	0.0	0.0	0.0	0.0	0.0	0.0
**SFR09759**	K14999	*CYP* 6	0.0	0.0	0.0	0.0	3.5	0.0	0.0	0.0	0.0	0.0	0.0	0.0	0.0	0.0	0.0	0.0	0.0	0.0	0.0	0.0	0.0	0.0	0.0
**SFR20883**	K14999	*CYP* 6	0.0	0.0	0.0	-2.2	0.0	-2.2	0.0	-2.2	-2.4	-1.7	-1.7	0.0	0.0	0.0	-1.9	0.0	-2.1	-2.5	-2.5	-2.5	-2.4	-2.5	-2.6
**SFR13922**	K00665	FASN	-1.7	0.0	-1.6	-2.5	0.0	0.0	-2.2	0.0	-1.9	-2.0	0.0	0.0	0.0	-2.3	-2.1	-2.2	-1.8	-2.2	0.0	-1.6	0.0	0.0	-1.9
**SFR19529**	K07418	*CYP* 2J	0.0	0.0	0.0	0.0	0.0	0.0	0.0	0.0	0.0	0.0	0.0	0.0	0.0	0.0	0.0	0.0	0.0	0.0	0.0	0.0	0.0	0.0	0.0
**SFR21138**	K14999	*CYP* 6	3.7	0.0	0.0	0.0	0.0	0.0	0.0	0.0	0.0	0.0	0.0	0.0	0.0	0.0	0.0	0.0	0.0	0.0	0.0	0.0	0.0	0.0	0.0

**SFR21139**	K14999	*CYP* 6	3.9	0.0	0.0	0.0	0.0	3.3	0.0	0.0	0.0	0.0	0.0	0.0	0.0	0.0	0.0	0.0	0.0	0.0	0.0	0.0	0.0	0.0	0.0
**SFR09405**	K14999	*CYP* 6	4.4	0.0	0.0	0.0	3.1	3.2	0.0	3.0	0.0	3.9	3.5	2.8	0.0	0.0	0.0	0.0	2.4	0.0	0.0	2.9	0.0	0.0	0.0
**SFR17683**	K00665	FASN	0.0	0.0	0.0	0.0	0.0	0.0	0.0	0.0	0.0	0.0	0.0	0.0	0.0	0.0	0.0	0.0	0.0	0.0	0.0	-3.2	0.0	0.0	0.0
**SFR06359**	K00665	FASN	0.0	0.0	0.0	0.0	0.0	0.0	0.0	0.0	0.0	-2.3	0.0	0.0	0.0	0.0	0.0	0.0	0.0	0.0	0.0	0.0	0.0	0.0	0.0
**SFR13626**	K15001	*CYP* 4	0.0	0.0	0.0	0.0	2.6	0.0	0.0	0.0	0.0	0.0	0.0	0.0	0.0	0.0	0.0	0.0	0.0	0.0	0.0	0.0	0.0	-2.0	0.0
**SFR14393**	K00665	FASN	0.0	0.0	0.0	0.0	0.0	0.0	0.0	0.0	0.0	0.0	0.0	0.0	0.0	0.0	0.0	0.0	0.0	-1.8	0.0	0.0	0.0	-1.5	0.0
**SFR00818**	K14999	*CYP* 6	0.0	0.0	0.0	0.0	0.0	0.0	0.0	0.0	0.0	-1.9	0.0	0.0	0.0	-2.1	0.0	0.0	0.0	0.0	0.0	0.0	0.0	0.0	0.0
**SFR01074**	K14999	*CYP* 6	0.0	0.0	0.0	0.0	3.7	0.0	3.0	2.1	0.0	0.0	0.0	0.0	0.0	-2.0	0.0	2.4	0.0	0.0	0.0	0.0	0.0	0.0	2.4
**SFR02735**	K14999	*CYP* 6	-3.3	0.0	0.0	-2.6	-2.0	-2.1	0.0	-3.0	0.0	-3.0	-3.1	0.0	-3.4	0.0	0.0	0.0	-2.2	-2.0	0.0	-1.7	-1.8	-2.2	-2.1
**SFR10855**	K14999	*CYP* 6	0.0	0.0	0.0	0.0	0.0	0.0	0.0	0.0	-2.3	0.0	0.0	0.0	0.0	0.0	0.0	0.0	0.0	0.0	0.0	0.0	0.0	0.0	0.0
**SFR11683**	K00665	FASN	0.0	0.0	0.0	0.0	0.0	0.0	0.0	1.1	0.0	0.0	0.0	0.0	0.0	0.0	0.0	0.0	0.0	0.0	0.0	0.0	0.0	0.0	0.0
**SFR12252**	K15001	*CYP* 4	0.0	0.0	0.0	0.0	2.4	0.0	1.9	0.0	0.0	0.0	0.0	0.0	0.0	0.0	0.0	0.0	0.0	0.0	0.0	0.0	0.0	0.0	0.0
**SFR13122**	K14999	*CYP* 6	0.0	0.0	0.0	0.0	3.2	0.0	0.0	2.9	0.0	0.0	0.0	0.0	0.0	2.5	0.0	0.0	0.0	0.0	0.0	0.0	-2.6	0.0	0.0
**SFR20024**	K15001	*CYP* 4	3.0	0.0	0.0	0.0	3.0	0.0	2.3	0.0	0.0	0.0	0.0	0.0	0.0	0.0	0.0	0.0	0.0	0.0	0.0	0.0	0.0	0.0	0.0
**SFR06338**	K14999	*CYP* 6	0.0	-2.5	0.0	-2.4	0.0	0.0	0.0	0.0	0.0	0.0	0.0	-2.3	-2.5	0.0	0.0	0.0	0.0	0.0	0.0	-2.2	0.0	0.0	-1.8
**SFR09487**	K00665	FASN	0.0	0.0	0.0	3.4	3.4	0.0	0.0	3.2	3.3	2.7	3.4	0.0	0.0	2.7	0.0	3.2	3.0	0.0	0.0	3.6	0.0	3.4	3.0
**SFR22090**	K14999	*CYP* 6	0.0	0.0	0.0	0.0	0.0	2.5	0.0	0.0	0.0	0.0	0.0	0.0	0.0	0.0	0.0	0.0	0.0	0.0	0.0	0.0	0.0	0.0	0.0
**SFR05237**	K14999	*CYP* 6	2.6	0.0	0.0	0.0	0.0	0.0	2.6	0.0	0.0	0.0	2.3	0.0	0.0	0.0	0.0	0.0	0.0	0.0	0.0	0.0	0.0	0.0	2.1
**SFR05333**	K14937	*CYP* 15A1_C1	0.0	0.0	0.0	-2.3	0.0	0.0	0.0	0.0	0.0	-1.9	0.0	0.0	0.0	0.0	0.0	0.0	0.0	0.0	0.0	-1.4	0.0	0.0	-1.9
**SFR19026**	K15003	*CYP* 9	0.0	0.0	0.0	0.0	0.0	0.0	0.0	0.0	0.0	0.0	0.0	0.0	-1.2	0.0	0.0	0.0	0.0	-1.4	-1.0	-1.4	0.0	0.0	0.0
**SFR19030**	K15003	*CYP* 9	0.0	0.0	0.0	0.0	-3.8	0.0	0.0	0.0	0.0	0.0	0.0	0.0	0.0	0.0	0.0	0.0	0.0	0.0	0.0	0.0	0.0	0.0	0.0
**SFR19033**	K15003	*CYP* 9	0.0	0.0	0.0	3.0	-4.0	0.0	0.0	0.0	0.0	4.1	0.0	0.0	0.0	3.9	0.0	0.0	3.7	0.0	0.0	0.0	3.7	3.9	0.0
**SFR00474**	K16484	RTTN	0.0	0.0	0.0	0.0	0.0	0.0	0.0	0.0	0.0	-1.6	0.0	0.0	0.0	0.0	0.0	0.0	-1.2	0.0	0.0	0.0	0.0	0.0	0.0
**SFR00608**	K14985	*CYP* 18A1	0.0	0.0	0.0	0.0	0.0	0.0	0.0	0.0	0.0	0.0	0.0	0.0	0.0	0.0	1.7	0.0	0.0	0.0	0.0	0.0	0.0	0.0	0.0
**SFR00610**	K14985	*CYP* 18A1	0.0	0.0	0.0	0.0	-2.1	-2.3	0.0	-3.5	0.0	0.0	0.0	0.0	0.0	0.0	0.0	0.0	0.0	0.0	0.0	0.0	0.0	0.0	0.0
**SFR08097**	K00665	FASN	3.3	0.0	3.5	3.3	3.0	0.0	0.0	3.6	3.2	3.0	3.0	0.0	0.0	2.8	0.0	3.1	3.5	3.2	0.0	3.6	0.0	3.0	3.0
**SFR09521**	K14999	*CYP* 6	0.0	0.0	2.1	1.7	0.0	0.0	0.0	0.0	0.0	1.9	0.0	0.0	0.0	0.0	0.0	0.0	0.0	0.0	0.0	0.0	0.0	0.0	0.0
**SFR09538**	K14999	*CYP* 6	0.0	0.0	2.2	0.0	0.0	0.0	0.0	0.0	0.0	1.8	0.0	0.0	0.0	0.0	0.0	0.0	0.0	0.0	0.0	0.0	0.0	0.0	0.0
**SFR09816**	K15001	*CYP* 4	1.7	3.1	0.0	0.0	0.0	2.0	3.1	4.2	3.1	0.0	3.2	0.0	0.0	1.4	0.0	0.0	2.5	2.3	2.5	2.4	0.0	1.7	0.0
**SFR13091**	K07427	*CYP* 4V2	3.5	0.0	0.0	0.0	0.0	0.0	2.5	0.0	0.0	0.0	0.0	0.0	0.0	0.0	0.0	0.0	0.0	0.0	0.0	0.0	0.0	0.0	0.0

**SFR13092**	K15001	*CYP* 4	0.0	0.0	0.0	-2.2	0.0	0.0	0.0	0.0	0.0	0.0	0.0	0.0	0.0	0.0	0.0	0.0	0.0	0.0	0.0	0.0	-1.5	0.0	0.0
**SFR13093**	K07427	*CYP* 4V2	4.8	0.0	0.0	0.0	0.0	0.0	0.0	0.0	0.0	0.0	0.0	0.0	0.0	0.0	0.0	0.0	0.0	0.0	0.0	0.0	0.0	0.0	0.0
**SFR13115**	K07427	*CYP* 4V2	0.0	0.0	0.0	0.0	0.0	0.0	0.0	3.1	0.0	0.0	0.0	0.0	0.0	0.0	0.0	0.0	0.0	0.0	0.0	0.0	0.0	1.8	0.0
**SFR14715**	K00665	FASN	0.0	0.0	0.0	0.0	0.0	0.0	0.0	0.0	0.0	0.0	0.0	0.0	0.0	0.0	0.0	0.0	0.0	0.0	0.0	0.0	0.0	0.0	0.0
**SFR09671**	K17960	*CYP* 49A	0.0	0.0	0.0	0.0	0.0	0.0	0.0	0.0	0.0	2.7	0.0	0.0	0.0	0.0	0.0	0.0	0.0	0.0	0.0	0.0	0.0	2.4	0.0
**SFR09673**	K17960	*CYP* 49A	0.0	0.0	0.0	2.7	2.4	0.0	2.9	0.0	0.0	3.4	2.9	2.9	0.0	2.3	2.7	2.7	3.1	3.1	2.4	2.3	0.0	3.6	2.5
**SFR10410**	K17960	*CYP* 49A	0.0	0.0	0.0	2.6	2.2	0.0	2.5	0.0	0.0	2.1	2.4	0.0	0.0	0.0	2.3	2.5	2.1	0.0	0.0	2.1	1.8	1.8	0.0
**SFR02818**	K15001	*CYP* 4	0.0	0.0	0.0	0.0	0.0	0.0	0.0	2.6	0.0	0.0	0.0	0.0	0.0	0.0	0.0	0.0	0.0	0.0	0.0	0.0	0.0	0.0	0.0
**SFR21906**	K10723	SHD	0.0	0.0	0.0	0.0	0.0	0.0	0.0	-1.9	-1.8	0.0	0.0	-2.8	0.0	0.0	0.0	0.0	-1.4	0.0	-1.1	-1.2	0.0	0.0	0.0
**SFR11880**	K15001	*CYP* 4	2.9	0.0	0.0	0.0	0.0	-2.7	-3.0	-2.2	0.0	0.0	0.0	0.0	0.0	0.0	0.0	0.0	0.0	0.0	0.0	0.0	0.0	0.0	0.0
**SFR11881**	K07427	*CYP* 4V2	4.3	0.0	0.0	0.0	2.6	0.0	0.0	0.0	0.0	0.0	0.0	0.0	0.0	0.0	0.0	0.0	0.0	0.0	0.0	0.0	0.0	0.0	0.0
**SFR11884**	K07427	*CYP* 4V2	6.2	0.0	0.0	3.0	3.6	0.0	0.0	0.0	0.0	0.0	0.0	0.0	3.3	0.0	0.0	2.8	3.2	0.0	3.9	0.0	0.0	3.0	3.0
**SFR11886**	K07427	*CYP* 4V2	4.5	0.0	0.0	2.3	0.0	0.0	0.0	4.6	0.0	0.0	0.0	0.0	0.0	0.0	0.0	0.0	0.0	0.0	0.0	0.0	0.0	0.0	0.0
**SFR11887**	K07427	*CYP* 4V2	0.0	0.0	0.0	0.0	0.0	0.0	0.0	0.0	0.0	0.0	2.7	0.0	0.0	0.0	0.0	0.0	0.0	0.0	0.0	0.0	0.0	0.0	0.0
**SFR15284**	K07427	*CYP* 4V2	0.0	0.0	0.0	0.0	0.0	0.0	0.0	-2.1	0.0	-2.1	0.0	0.0	0.0	0.0	0.0	0.0	0.0	0.0	0.0	0.0	0.0	0.0	0.0
**SFR21631**	K00665	FASN	0.0	0.0	0.0	0.0	0.0	0.0	0.0	0.0	0.0	0.0	0.0	0.0	0.0	0.0	0.0	0.0	0.0	0.0	0.0	-3.1	0.0	0.0	0.0
**SFR09608**	K15001	*CYP* 4	0.0	0.0	0.0	0.0	0.0	1.8	0.0	0.0	2.3	0.0	0.0	2.4	0.0	0.0	0.0	0.0	0.0	1.9	0.0	0.0	0.0	0.0	0.0
**SFR14812**	K14939	SPO	0.0	0.0	0.0	0.0	0.0	0.0	0.0	-2.6	0.0	0.0	0.0	0.0	0.0	0.0	0.0	0.0	-2.1	0.0	0.0	0.0	0.0	0.0	0.0
**SFR19924**	K14999	*CYP* 6	0.0	0.0	0.0	-2.8	0.0	0.0	0.0	-2.4	0.0	0.0	-2.6	-2.8	0.0	0.0	-2.1	0.0	-1.9	-2.5	-2.3	-2.6	0.0	-2.3	-3.3
**SFR02268**	K07427	*CYP* 4V2	3.0	0.0	0.0	0.0	0.0	0.0	0.0	0.0	0.0	0.0	0.0	0.0	0.0	0.0	0.0	0.0	0.0	0.0	0.0	0.0	0.0	0.0	0.0
**SFR05805**	K14999	*CYP* 6	0.0	0.0	0.0	0.0	0.0	0.0	0.0	0.0	0.0	0.0	0.0	0.0	0.0	0.0	0.0	0.0	0.0	0.0	0.0	-2.3	0.0	0.0	0.0
**SFR01390**	K07427	*CYP* 4V2	4.2	0.0	0.0	0.0	0.0	0.0	0.0	0.0	0.0	0.0	0.0	0.0	0.0	0.0	0.0	0.0	0.0	0.0	0.0	0.0	0.0	0.0	0.0
**SFR17475**	K07427	*CYP* 4V2	0.0	0.0	0.0	0.0	0.0	2.3	0.0	2.4	0.0	1.6	0.0	0.0	0.0	0.0	0.0	0.0	0.0	0.0	0.0	0.0	0.0	0.0	0.0
**SFR17294**	K07427	*CYP* 4V2	0.0	0.0	0.0	2.2	0.0	0.0	0.0	2.5	1.9	0.0	2.2	0.0	0.0	0.0	0.0	0.0	0.0	0.0	0.0	1.6	0.0	0.0	0.0
**SFR20970**	K15001	*CYP* 4	0.0	0.0	0.0	2.2	2.1	0.0	2.4	3.3	2.3	1.7	2.2	0.0	0.0	0.0	0.0	1.8	2.0	2.2	2.3	0.0	0.0	2.3	0.0
**SFR00950**	K15004	*CYP* 12	0.0	0.0	0.0	0.0	0.0	0.0	2.2	0.0	0.0	0.0	0.0	0.0	0.0	0.0	0.0	0.0	0.0	0.0	0.0	0.0	0.0	0.0	0.0
**SFR00951**	K15004	*CYP* 12	0.0	0.0	0.0	0.0	0.0	0.0	2.2	0.0	0.0	0.0	0.0	0.0	0.0	0.0	0.0	0.0	0.0	0.0	0.0	0.0	0.0	0.0	0.0
**SFR17237**	K15001	*CYP* 4	1.9	2.7	0.0	0.0	0.0	2.6	3.5	4.1	3.1	1.3	3.3	2.0	0.0	0.0	0.0	1.7	2.3	1.6	1.9	1.7	0.0	0.0	0.0
**SFR20076**	K07427	*CYP* 4V2	3.9	0.0	0.0	0.0	0.0	0.0	0.0	0.0	0.0	0.0	0.0	0.0	0.0	0.0	0.0	3.0	0.0	0.0	2.8	0.0	0.0	0.0	2.7
**SFR20561**	K07427	*CYP* 4V2	0.0	0.0	0.0	2.7	0.0	3.8	1.6	3.9	2.4	2.2	2.9	0.0	0.0	0.0	0.0	0.0	2.3	0.0	0.0	1.5	0.0	0.0	0.0

**SFR20450**	K14999	*CYP* 6	0.0	0.0	0.0	-2.4	0.0	-2.2	0.0	0.0	-1.8	-1.3	-2.2	0.0	0.0	0.0	-2.6	0.0	-1.5	-3.3	0.0	-2.2	-2.8	-2.1	-2.5
**SFR20451**	K14999	*CYP* 6	0.0	0.0	0.0	-2.5	0.0	-2.2	0.0	-1.4	-1.9	-1.4	-2.3	0.0	0.0	0.0	-2.7	0.0	-1.6	-3.4	-1.7	-2.3	-2.8	-2.3	-2.6
**SFR14275**	K15001	*CYP* 4	2.5	3.7	0.0	0.0	0.0	2.0	3.1	4.5	3.5	0.0	3.4	0.0	0.0	1.8	0.0	0.0	2.2	2.6	2.7	2.4	0.0	1.6	0.0
**SFR00954**	K14999	*CYP* 6	0.0	0.0	0.0	0.0	0.0	0.0	0.0	0.0	0.0	0.0	0.0	0.0	0.0	0.0	0.0	0.0	0.0	0.0	0.0	0.0	0.0	0.0	0.0
**SFR21423**	K00665	FASN	0.0	0.0	0.0	0.0	0.0	0.0	0.0	0.0	0.0	0.0	0.0	0.0	0.0	0.0	0.0	0.0	0.0	0.0	-3.3	0.0	0.0	0.0	0.0
**SFR12623**	K07427	*CYP* 4V2	0.0	0.0	0.0	0.0	0.0	0.0	0.0	3.0	0.0	0.0	0.0	0.0	0.0	0.0	0.0	0.0	0.0	0.0	0.0	0.0	0.0	1.8	0.0
**SFR14585**	K00665	FASN	0.0	0.0	0.0	0.0	0.0	0.0	0.0	0.0	0.0	-3.5	0.0	0.0	0.0	0.0	0.0	-3.9	0.0	0.0	0.0	0.0	0.0	0.0	0.0
**SFR14295**	K15001	*CYP* 4	0.0	0.0	0.0	0.0	0.0	0.0	0.0	0.0	0.0	0.0	0.0	0.0	0.0	0.0	0.0	0.0	0.0	0.0	2.3	0.0	0.0	0.0	0.0
**SFR17550**	K07427	*CYP* 4V2	0.0	0.0	4.3	3.1	0.0	4.4	6.2	0.0	5.3	0.0	0.0	0.0	0.0	0.0	4.3	0.0	0.0	0.0	0.0	0.0	0.0	0.0	0.0
**SFR14625**	K00665	FASN	0.0	0.0	0.0	0.0	0.0	0.0	0.0	0.0	0.0	0.0	0.0	0.0	0.0	0.0	0.0	0.0	0.0	-2.1	-2.2	0.0	0.0	0.0	0.0
**SFR20036**	K07427	*CYP* 4V2	0.0	0.0	0.0	0.0	0.0	0.0	0.0	0.0	0.0	-3.1	0.0	0.0	0.0	0.0	0.0	0.0	0.0	0.0	0.0	0.0	0.0	0.0	0.0
**SFR06492**	K14999	*CYP* 6	0.0	0.0	0.0	0.0	0.0	0.0	0.0	0.0	0.0	0.0	0.0	0.0	0.0	0.0	0.0	0.0	0.0	0.0	0.0	0.0	-2.3	0.0	0.0
**SFR12089**	K07427	*CYP* 4V2	0.0	0.0	0.0	0.0	0.0	0.0	2.6	0.0	0.0	0.0	0.0	0.0	0.0	0.0	0.0	0.0	0.0	0.0	0.0	0.0	0.0	0.0	0.0
**SFR20528**	K07427	*CYP* 4V2	0.0	0.0	0.0	0.0	0.0	0.0	2.8	2.3	0.0	0.0	0.0	0.0	0.0	0.0	0.0	0.0	0.0	0.0	0.0	0.0	0.0	0.0	0.0
**SFR19543**	K14999	*CYP* 6	3.6	0.0	4.9	0.0	0.0	0.0	0.0	0.0	0.0	0.0	0.0	0.0	0.0	2.7	0.0	0.0	0.0	0.0	0.0	0.0	0.0	0.0	0.0
**SFR20982**	K00665	FASN	0.0	0.0	2.8	2.9	3.2	0.0	0.0	2.5	2.7	2.7	3.3	0.0	0.0	2.7	0.0	2.9	2.8	3.3	0.0	3.4	0.0	2.9	3.8
**SFR05026**	K07427	*CYP* 4V2	0.0	0.0	0.0	0.0	0.0	0.0	0.0	0.0	0.0	0.0	0.0	0.0	0.0	0.0	0.0	0.0	0.0	0.0	0.0	0.0	0.0	0.0	0.0
**SFR11808**	K07427	*CYP* 4V2	0.0	0.0	0.0	0.0	0.0	0.0	0.0	0.0	0.0	-2.6	-2.5	0.0	0.0	0.0	0.0	0.0	-2.6	0.0	0.0	0.0	0.0	-2.5	-2.4
**SFR20959**	K14999	*CYP* 6	0.0	0.0	0.0	0.0	0.0	0.0	0.0	0.0	0.0	-2.1	-1.9	0.0	-2.5	0.0	-2.4	0.0	0.0	-3.1	0.0	-2.7	-2.6	-2.3	-3.1
**SFR20960**	K14999	*CYP* 6	0.0	0.0	0.0	0.0	0.0	-1.9	0.0	0.0	0.0	-2.3	-2.4	0.0	-2.7	0.0	-2.7	0.0	0.0	-3.7	-2.0	-2.6	-2.9	-2.7	-3.2
**SFR18467**	K15003	*CYP* 9	0.0	-3.2	0.0	-2.1	0.0	-1.7	0.0	-3.0	-1.7	-1.6	-2.3	-2.4	0.0	0.0	0.0	-1.4	-2.2	-1.9	-2.0	-2.8	-1.6	-2.8	-1.7
**SFR05875**	K15001	*CYP* 4	0.0	0.0	0.0	0.0	0.0	0.0	0.0	0.0	0.0	0.0	0.0	0.0	0.0	0.0	0.0	0.0	0.0	0.0	0.0	0.0	0.0	1.2	0.0
**SFR19922**	K14999	*CYP* 6	0.0	0.0	0.0	-2.5	0.0	-3.0	0.0	-1.8	0.0	0.0	0.0	-3.2	-2.5	0.0	0.0	0.0	0.0	-2.2	-2.6	-2.5	-2.4	-2.2	-2.6

Table S19 | The DEGs of Azadirachtin significantly enriched in 34 KEGG pathway.

#Term	ID	Input number	Background number	P-Value	Corrected P-Value	Input
**Metabolic pathways**	hsa01100	63	1243	6.57E-29	7.87E-27	SFR13509|SFR15666|SFR17845|SFR13761|SFR12967|SFR22106|SFR03885|SFR20883|SFR05689|SFR06338|SFR05602|SFR16595|SFR13178|SFR19435|SFR13913|SFR18232|SFR15870|SFR01157|SFR13030|SFR13174|SFR13176|SFR12103|SFR03661|SFR06067|SFR10410|SFR15191|SFR08977|SFR12470|SFR18509|SFR18647|SFR19632|SFR13323|SFR05333|SFR19924|SFR05415|SFR21290|SFR20660|SFR00991|SFR15133|SFR03171|SFR17913|SFR20450|SFR20451|SFR08155|SFR02735|SFR02133|SFR20350|SFR17310|SFR18501|SFR03637|SFR03023|SFR02520|SFR02217|SFR19620|SFR05789|SFR04436|SFR02148|SFR02723|SFR17953|SFR05548|SFR13563|SFR20619|SFR07202
**Peroxisome**	hsa04146	20	83	1.53E-21	1.16E-19	SFR21337|SFR14877|SFR02520|SFR04997|SFR03768|SFR03885|SFR14884|SFR14879|SFR16238|SFR18509|SFR13601|SFR10391|SFR10392|SFR10393|SFR17913|SFR05602|SFR04770|SFR15128|SFR05605|SFR18740
**Chemical carcinogenesis**	hsa05204	18	82	6.91E-19	3.72E-17	SFR21040|SFR13178|SFR20350|SFR19924|SFR17310|SFR02735|SFR13174|SFR20451|SFR13176|SFR12103|SFR02217|SFR20883|SFR17939|SFR06338|SFR18467|SFR20450|SFR16595|SFR05333
**Retinol metabolism**	hsa00830	15	65	3.09E-16	1.33E-14	SFR13178|SFR19924|SFR02859|SFR02735|SFR13174|SFR20451|SFR13176|SFR12103|SFR03661|SFR02217|SFR20883|SFR02723|SFR06338|SFR20450|SFR16595

**Drug metabolism - cytochrome P450**	hsa00982	15	69	6.68E-16	2.77E-14	SFR13178|SFR20350|SFR19924|SFR02735|SFR02217|SFR20451|SFR13176|SFR12103|SFR03661|SFR20883|SFR17939|SFR06338|SFR20450|SFR16595|SFR05333
**Metabolism of xenobiotics by cytochrome P450**	hsa00980	15	73	1.39E-15	5.49E-14	SFR21040|SFR13178|SFR20350|SFR19924|SFR17310|SFR02735|SFR02217|SFR20451|SFR13176|SFR12103|SFR20883|SFR17939|SFR06338|SFR20450|SFR16595
**Steroid hormone biosynthesis**	hsa00140	13	58	4.18E-14	1.45E-12	SFR13178|SFR19924|SFR02217|SFR02735|SFR13174|SFR20451|SFR13176|SFR12103|SFR22106|SFR20883|SFR06338|SFR20450|SFR16595
**Ribosome biogenesis in eukaryotes**	hsa03008	14	89	3.22E-13	1.01E-11	SFR03498|SFR10638|SFR10636|SFR18709|SFR17185|SFR03868|SFR12386|SFR11573|SFR17946|SFR03892|SFR02707|SFR20236|SFR18907|SFR05621
**Glycine, serine and threonine metabolism**	hsa00260	8	40	7.73E-09	1.45E-07	SFR13509|SFR05415|SFR02520|SFR06067|SFR15133|SFR17913|SFR05602|SFR19632
**Drug metabolism - other enzymes**	hsa00983	8	46	2.04E-08	3.52E-07	SFR13178|SFR02217|SFR13176|SFR12103|SFR03885|SFR20883|SFR18509|SFR16595
**Linoleic acid metabolism**	hsa00591	7	29	2.21E-08	3.80E-07	SFR13178|SFR02217|SFR13176|SFR20883|SFR16595|SFR08155|SFR05333
**Bile secretion**	hsa04976	9	71	3.15E-08	5.35E-07	SFR21040|SFR13178|SFR16952|SFR02217|SFR13176|SFR12103|SFR20883|SFR21413|SFR16595
**Purine metabolism**	hsa00230	11	176	7.70E-07	1.03E-05	SFR02133|SFR18501|SFR13913|SFR12895|SFR01157|SFR00991|SFR17247|SFR03885|SFR05773|SFR18509|SFR02148
**Alanine, aspartate and glutamate**	hsa00250	6	35	1.35E-06	1.74E-05	SFR13761|SFR02520|SFR05789|SFR17913|SFR05602|SFR

**metabolism**						15128
**Amino sugar and nucleotide sugar metabolism**	hsa00520	6	48	7.08E-06	8.03E-05	SFR03023|SFR13030|SFR05789|SFR13563|SFR20619|SFR18647
**Carbon metabolism**	hsa01200	8	113	1.07E-05	0.000116	SFR03637|SFR18232|SFR15870|SFR02520|SFR17953|SFR17913|SFR03171|SFR05602
**ECM-receptor interaction**	hsa04512	6	82	0.000116	0.001017	SFR19174|SFR16368|SFR11223|SFR11222|SFR04584|SFR07272
**Arachidonic acid metabolism**	hsa00590	5	62	0.000286	0.00228	SFR20350|SFR13323|SFR17310|SFR08155|SFR05333
**Amoebiasis**	hsa05146	6	100	0.000321	0.002539	SFR16774|SFR06354|SFR11223|SFR11222|SFR04584|SFR21938
**Aminoacyl-tRNA biosynthesis**	hsa00970	5	66	0.000374	0.002903	SFR15821|SFR11257|SFR05153|SFR05401|SFR04884
**Glycolysis / Gluconeogenesis**	hsa00010	5	67	0.000399	0.00307	SFR03637|SFR05689|SFR03171|SFR15666|SFR18232
**Other glycan degradation**	hsa00511	3	18	0.000748	0.005395	SFR09227|SFR15191|SFR00638
**Complement and coagulation cascades**	hsa04610	5	79	0.00081	0.005804	SFR12411|SFR12407|SFR09068|SFR00831|SFR09065
**Terpenoid backbone biosynthesis**	hsa00900	3	22	0.001261	0.008311	SFR16674|SFR19435|SFR08902

**Caffeine metabolism**	hsa00232	2	5	0.001506	0.009425	SFR18509|SFR03885
**Glycerophospholipid metabolism**	hsa00564	5	95	0.001768	0.010889	SFR21290|SFR12967|SFR05246|SFR08155|SFR18201
**Glyoxylate and dicarboxylate metabolism**	hsa00630	3	28	0.002373	0.013807	SFR17913|SFR05602|SFR02520
**RNA polymerase**	hsa03020	3	32	0.003368	0.017953	SFR18501|SFR01157|SFR00991
**Fructose and mannose metabolism**	hsa00051	3	33	0.003651	0.019201	SFR17845|SFR03709|SFR13738
**Pentose and glucuronate interconversions**	hsa00040	3	36	0.004586	0.023146	SFR17845|SFR12103|SFR15666
**Influenza A**	hsa05164	6	176	0.005093	0.025388	SFR03790|SFR19435|SFR14423|SFR08994|SFR20610|SFR 21469
**Fat digestion and absorption**	hsa04975	3	41	0.006439	0.029952	SFR16952|SFR21323|SFR08155
**Spliceosome**	hsa03040	5	134	0.007205	0.032668	SFR14272|SFR18565|SFR08066|SFR03932|SFR10636
**Protein digestion and absorption**	hsa04974	4	90	0.008948	0.039011	SFR04584|SFR21469|SFR11223|SFR11222

Table S20 | Information of 240 samples used in this study

Sample Identifer	Species	Sampling locations	geographic location - latitude	geographic location - longitude	collection date	Tissue	Sex	Developmental stages
SFgdWGS	*Spodoptera frugiperda*	China:Guangdong:Guangzhou	N 23° 25'41"	E 113° 22'07"	2019/5/24	whole body	Unknown	Sixth-instar
SFynMstLFR	*S. frugiperda*	China:YunNan:Kunming	N 25° 01'00"	E 102° 50'34"	2019/5/22	whole body	Male	Adult
SFgdRNA_1	*S. frugiperda*	China:Guangdong:Guangzhou	N 23° 25'41"	E 113° 22'07"	2019/5/24	whole body	Unknown	Fifth-instar
SFgdRNA_2	*S. frugiperda*	China:Guangdong:Guangzhou	N 23° 25'41"	E 113° 22'07"	2019/5/24	whole body	Unknown	Sixth-instar
SFYN	*S. frugiperda*	China:YunNan:Kunming	N 25° 01'00"	E 102° 50'34"	2019/5/22	whole body	Unknown	Adult
A1	*S. frugiperda*	USA:LA:Franklin Parish	N 32°41'34.31"	W 91°41'34.31"	2019/8/1	thorax of adult	Unknown	Adult
A10	*S. frugiperda*	USA:LA:Franklin Parish	N 32°41'34.31"	W 91°41'34.31"	2019/8/1	thorax of adult	Unknown	Adult
A2	*S. frugiperda*	USA:LA:Franklin Parish	N 32°41'34.31"	W 91°41'34.31"	2019/8/1	thorax of adult	Unknown	Adult
A3	*S. frugiperda*	USA:LA:Franklin Parish	N 32°41'34.31"	W 91°41'34.31"	2019/8/1	thorax of adult	Unknown	Adult
A4	*S. frugiperda*	USA:LA:Franklin Parish	N 32°41'34.31"	W 91°41'34.31"	2019/8/1	thorax of adult	Unknown	Adult
A5	*S. frugiperda*	USA:LA:Franklin Parish	N 32°41'34.31"	W 91°41'34.31"	2019/8/1	thorax of adult	Unknown	Adult
A6	*S. frugiperda*	USA:LA:Franklin Parish	N 32°41'34.31"	W 91°41'34.31"	2019/8/1	thorax of adult	Unknown	Adult
A7	*S. frugiperda*	USA:LA:Franklin Parish	N 32°41'34.31"	W 91°41'34.31"	2019/8/1	thorax of adult	Unknown	Adult
A8	*S. frugiperda*	USA:LA:Franklin Parish	N 32°41'34.31"	W 91°41'34.31"	2019/8/1	thorax of adult	Unknown	Adult
A9	*S. frugiperda*	USA:LA:Franklin Parish	N 32°41'34.31"	W 91°41'34.31"	2019/8/1	thorax of adult	Unknown	Adult
B1	*S. frugiperda*	USA:LA:East Baton Rouge Parish	N 30°24'15.05"	W 91°10'39.66"	2017	thorax of adult	Unknown	Adult

B10	*S. frugiperda*	USA:LA:East Baton Rouge Parish	N 30°24'15.05"	W 91°10'39.66"	2017	thorax of adult	Unknown	Adult
B2	*S. frugiperda*	USA:LA:East Baton Rouge Parish	N 30°24'15.05"	W 91°10'39.66"	2017	thorax of adult	Unknown	Adult
B3	*S. frugiperda*	USA:LA:East Baton Rouge Parish	N 30°24'15.05"	W 91°10'39.66"	2017	thorax of adult	Unknown	Adult
B4	*S. frugiperda*	USA:LA:East Baton Rouge Parish	N 30°24'15.05"	W 91°10'39.66"	2017	thorax of adult	Unknown	Adult
B5	*S. frugiperda*	USA:LA:East Baton Rouge Parish	N 30°24'15.05"	W 91°10'39.66"	2017	thorax of adult	Unknown	Adult
B6	*S. frugiperda*	USA:LA:East Baton Rouge Parish	N 30°24'15.05"	W 91°10'39.66"	2017	thorax of adult	Unknown	Adult
B7	*S. frugiperda*	USA:LA:East Baton Rouge Parish	N 30°24'15.05"	W 91°10'39.66"	2017	thorax of adult	Unknown	Adult
B8	*S. frugiperda*	USA:LA:East Baton Rouge Parish	N 30°24'15.05"	W 91°10'39.66"	2017	thorax of adult	Unknown	Adult
B9	*S. frugiperda*	USA:LA:East Baton Rouge Parish	N 30°24'15.05"	W 91°10'39.66"	2017	thorax of adult	Unknown	Adult
BS01	*S. frugiperda*	China:YunNan: Baoshan	N 24° 98'04"	E 99° 12'45"	2019/6/20	whole body	Unknown	Larvae
BS02	*S. frugiperda*	China:YunNan: Baoshan	N 24° 98'04"	E 99° 12'45"	2019/6/20	whole body	Unknown	Larvae
C1	*S. frugiperda*	USA:LA:Franklin Parish	N 32°08'21.08"	W 91°41'12.37"	2016/10/1	thorax of adult	Unknown	Adult
C10	*S. frugiperda*	USA:LA:Franklin Parish	N 32°08'21.08"	W 91°41'12.37"	2016/10/1	thorax of adult	Unknown	Adult
C2	*S. frugiperda*	USA:LA:Franklin Parish	N 32°08'21.08"	W 91°41'12.37"	2016/10/1	thorax of adult	Unknown	Adult
C3	*S. frugiperda*	USA:LA:Franklin Parish	N 32°08'21.08"	W 91°41'12.37"	2016/10/1	thorax of adult	Unknown	Adult
C4	*S. frugiperda*	USA:LA:Franklin Parish	N 32°08'21.08"	W 91°41'12.37"	2016/10/1	thorax of adult	Unknown	Adult
C6	*S. frugiperda*	USA:LA:Franklin Parish	N 32°08'21.08"	W 91°41'12.37"	2016/10/1	thorax of adult	Unknown	Adult
C7	*S. frugiperda*	USA:LA:Franklin Parish	N 32°08'21.08"	W 91°41'12.37"	2016/10/1	thorax of adult	Unknown	Adult

C8	*S. frugiperda*	USA:LA:Franklin Parish	N 32°08'21.08"	W 91°41'12.37"	2016/10/1	thorax of adult	Unknown	Adult
C9	*S. frugiperda*	USA:LA:Franklin Parish	N 32°08'21.08"	W 91°41'12.37"	2016/10/1	thorax of adult	Unknown	Adult
D1	*S. frugiperda*	USA:LA:Franklin Parish	Unknown	Unknown	Unknown	thorax of adult	Unknown	Adult
D10	*S. frugiperda*	USA:LA:Franklin Parish	Unknown	Unknown	Unknown	thorax of adult	Unknown	Adult
D2	*S. frugiperda*	USA:LA:Franklin Parish	Unknown	Unknown	Unknown	thorax of adult	Unknown	Adult
D3	*S. frugiperda*	USA:LA:Franklin Parish	Unknown	Unknown	Unknown	thorax of adult	Unknown	Adult
D4	*S. frugiperda*	USA:LA:Franklin Parish	Unknown	Unknown	Unknown	thorax of adult	Unknown	Adult
D5	*S. frugiperda*	USA:LA:Franklin Parish	Unknown	Unknown	Unknown	thorax of adult	Unknown	Adult
D6	*S. frugiperda*	USA:LA:Franklin Parish	Unknown	Unknown	Unknown	thorax of adult	Unknown	Adult
D7	*S. frugiperda*	USA:LA:Franklin Parish	Unknown	Unknown	Unknown	thorax of adult	Unknown	Adult
D8	*S. frugiperda*	USA:LA:Franklin Parish	Unknown	Unknown	Unknown	thorax of adult	Unknown	Adult
D9	*S. frugiperda*	USA:LA:Franklin Parish	Unknown	Unknown	Unknown	thorax of adult	Unknown	Adult
FAW1.1	*S. frugiperda*	South Africa:Gauteng	Unknown (sample from culture)	Unknown (sample from culture)	2019/6/1	Head and epithelial tissue	Unknown	Sixth-instar
FAW1.2	*S. frugiperda*	South Africa:Gauteng	Unknown (sample from culture)	Unknown (sample from culture)	2019/6/1	Head and epithelial tissue	Unknown	Sixth-instar
FAW2.1	*S. frugiperda*	South Africa:Gauteng	Unknown (sample from culture)	Unknown (sample from culture)	2019/6/1	Head and epithelial tissue	Unknown	Sixth-instar
FAW2.2	*S. frugiperda*	South Africa:Gauteng	Unknown (sample from culture)	Unknown (sample from culture)	2019/6/1	Head and epithelial tissue	Unknown	Sixth-instar

FAW5.2	*S. frugiperda*	South Africa:Limpopo:Tshiombo	S22.8046033	E30.4972486	2019/10/1	Head and epithelial tissue	Unknown	Sixth-instar
FJ01_1	*S. frugiperda*	China: YunNan: Funing	N 23° 34'76"	E 105° 37'34"	2019/5/30	whole body	Unknown	Adult
FJ01_2	*S. frugiperda*	China: YunNan: Funing	N 23° 34'76"	E 105° 37'34"	2019/5/30	whole body	Unknown	Adult
FJ01_3	*S. frugiperda*	China: YunNan: Funing	N 23° 34'76"	E 105° 37'34"	2019/5/30	whole body	Unknown	Adult
GX01	*S. frugiperda*	China:GuangXi:Nanning	N 22°48'02"	E 108°11'30"	2019/7/6	whole body	Unknown	Larvae
GX02	*S. frugiperda*	China:GuangXi:Nanning	N 22°48'02"	E 108°11'30"	2019/7/6	whole body	Unknown	Larvae
GX03	*S. frugiperda*	China:GuangXi:Nanning	N 22°48'02"	E 108°11'30"	2019/7/6	whole body	Unknown	Larvae
GX04	*S. frugiperda*	China:GuangXi:Nanning	N 22°48'02"	E 108°11'30"	2019/7/6	whole body	Unknown	Larvae
JC01	*S. frugiperda*	China:YunNan:Jiangcheng	N 22°58'51"	E 101°86'23"	2019/5/31	whole body	Unknown	Larvae
KE-1	*S. frugiperda*	Kenya:Naivasha-Nakuru	S0°53'44.3"	E36°11'48.4"	2019/1/3	muscle tissue	Unknown	Larvae
KE-10	*S. frugiperda*	Kenya:Migori-Trans Nzoia	N 01°09.411'	E 034° 58.441'	2018/12/28	muscle tissue	Unknown	Larvae
KE-3	*S. frugiperda*	Kenya:Meru	S0°06'05.9"	E37°46'57.1"	2018/12/22	muscle tissue	Unknown	Larvae
KE-4	*S. frugiperda*	Kenya:Kilifi	S03.16561	E040.00819	2018/12/22	muscle tissue	Unknown	Larvae
KE-5	*S. frugiperda*	Kenya:Makueni	S1°48'17.1"	E37°34'07.6"	2018/12/23	muscle tissue	Unknown	Larvae
KE-6	*S. frugiperda*	Kenya:Nyeri	S0°32'25.5"	E36°58'44.0"	2018/12/24	muscle tissue	Unknown	Larvae
KE-7	*S. frugiperda*	Kenya:Bungoma	N 00° 35.763'	E 034° 26.060'	2018/12/25	muscle tissue	Unknown	Larvae
KE-8	*S. frugiperda*	Kenya:Bomet	S0°51'59.4"	E35°23'17.2"	2018/12/26	muscle tissue	Unknown	Larvae
KE-9	*S. frugiperda*	Kenya:Taita Taveta	S03.39148	E038.57782	2018/12/27	muscle tissue	Unknown	Larvae
LJ01	*S. frugiperda*	China:YunNan:Lijiang	N 26°87'72"	E 100°22'54"	2019/7/15	whole body	Unknown	Larvae
LL01_2	*S. frugiperda*	China:YunNan:Luliang	N 25°02'94"	E 103°66'68"	2019/7/18	whole body	Unknown	Larvae
LL01_3	*S. frugiperda*	China:YunNan:Luliang	N 25°02'94"	E 103°66'68"	2019/7/18	whole body	Unknown	Larvae
LL04	*S. frugiperda*	China:YunNan:Luliang	N 25°02'94"	E 103°66'68"	2019/7/18	whole body	Unknown	Larvae
LL05	*S. frugiperda*	China:YunNan:Luliang	N 25°02'94"	E 103°66'68"	2019/7/18	whole body	Unknown	Larvae
LL06	*S. frugiperda*	China:YunNan:Luliang	N 25°02'94"	E 103°66'68"	2019/7/18	whole body	Unknown	Larvae

LL09	*S. frugiperda*	China:YunNan:Luliang	N 25°02'94"	E 103°66'68"	2019/7/18	whole body	Unknown	Larvae
MH01	*S. frugiperda*	China:YunNan:Menghai	N 21°95'75"	E 100°45'22"	2019/7/4	whole body	Unknown	Larvae
MH03	*S. frugiperda*	China:YunNan:Menghai	N 21°95'75"	E 100°45'22"	2019/7/4	whole body	Unknown	Larvae
ML01	*S. frugiperda*	China:YunNan:Malong	N 25°42'80"	E 103°57'83"	2019/6/3	whole body	Unknown	Larvae
ML01_1	*S. frugiperda*	China:YunNan:Malong	N 25°42'80"	E 103°57'83"	2019/6/3	whole body	Unknown	Larvae
ML01_3	*S. frugiperda*	China:YunNan:Malong	N 25°42'80"	E 103°57'83"	2019/6/3	whole body	Unknown	Larvae
ML1	*S. frugiperda*	Ethiopia:Oromia region:Melkassa	N8°24'55.2"	E39°19'39.2"	2019/3/10	muscle tissue	Unknown	Larvea
ML2	*S. frugiperda*	Ethiopia:Oromia region:Melkassa	N8°24'55.2"	E39°19'39.2"	2019/3/10	muscle tissue	Unknown	Larvea
ML3	*S. frugiperda*	Ethiopia:Oromia region:Melkassa	N8°24'55.2"	E39°19'39.2"	2019/3/10	muscle tissue	Unknown	Larvea
ML4	*S. frugiperda*	Ethiopia:Oromia region:Melkassa	N8°24'55.2"	E39°19'39.2"	2019/3/10	muscle tissue	Unknown	Larvea
ML5	*S. frugiperda*	Ethiopia:Oromia region:Melkassa	N8°24'55.2"	E39°19'39.2"	2019/3/10	muscle tissue	Unknown	Larvea
MS004	*S. frugiperda*	China:YunNan:Mangshi	N 24°43'37"	E 98°58'81"	2019/7/5	whole body	Unknown	Adult
MS03	*S. frugiperda*	China:YunNan:Mangshi	N 24°43'37"	E 98°58'81"	2019/7/5	whole body	Unknown	Adult
MZ01	*S.* frugiperda	China:YunNan:Mengzi	N 23°39'62"	E 103°36'48"	2019/7/16	whole body	Unknown	Adult
NA1	*S. frugiperda*	Ethiopia:Oromia region:Arsi Negele	N7°.09'07.41"	E38.25'54"	2019/8/19	muscle tissue	Unknown	Larvea
NA12	*S. frugiperda*	Ethiopia:Oromia region:Arsi Negele	N7°.09'07.41"	E38.25'54"	2019/8/19	muscle tissue	Unknown	Larvea
NA14	*S. frugiperda*	Ethiopia:Oromia region:Arsi Negele	N7°.09'07.41"	E38.25'54"	2019/8/19	muscle tissue	Unknown	Larvea
NA15	*S. frugiperda*	Ethiopia:Oromia region:Arsi Negele	N7°.09'07.41"	E38.25'54"	2019/8/19	muscle tissue	Unknown	Larvea
NA4	*S. frugiperda*	Ethiopia:Oromia region:Arsi Negele	N7°.09'07.41"	E38.25'54"	2019/8/19	muscle tissue	Unknown	Larvea
NJ01	*S. frugiperda*	China:YunNan:Nujiang	N 25°81'76"	E 98°85'67"	2019/7/25	whole body	Unknown	Larvae
NJ02	*S. frugiperda*	China:YunNan:Nujiang	N 25°81'76"	E 98°85'67"	2019/7/25	whole body	Unknown	Larvae
NJ03	*S. frugiperda*	China:YunNan:Nujiang	N 25°81'76"	E 98°85'67"	2019/7/25	whole body	Unknown	Larvae
NJ04	*S. frugiperda*	China:YunNan:Nujiang	N 25°81'76"	E 98°85'67"	2019/7/25	whole body	Unknown	Larvae
NJ05	*S. frugiperda*	China:YunNan:Nujiang	N 25°81'76"	E 98°85'67"	2019/7/25	whole body	Unknown	Larvae

NJ06	*S. frugiperda*	China:YunNan:Nujiang	N 25°81'76''	E 98°85'67''	2019/7/25	whole body	Unknown	Larvae
NJ07	*S. frugiperda*	China:YunNan:Nujiang	N 25°81'76''	E 98°85'67''	2019/7/25	whole body	Unknown	Larvae
NJ08	*S. frugiperda*	China:YunNan:Nujiang	N 25°81'76''	E 98°85'67''	2019/7/25	whole body	Unknown	Larvae
SC03	*S. frugiperda*	China:GuangXi:Nanning	N 22°48'02''	E 108°11'30''	2019/5/25	muscle tissue	Unknown	Larvae
SC08	*S. frugiperda*	China:GuangXi:Nanning	N 22°48'02''	E 108°11'30''	2019/5/26	muscle tissue	Unknown	Larvae
SC10	*S. frugiperda*	China:GuangXi:Nanning	N 22°48'02''	E 108°11'30''	2019/5/27	muscle tissue	Unknown	Larvae
SC11	*S. frugiperda*	China:GuangXi:Nanning	N 22°48'02''	E 108°11'30''	2019/5/28	muscle tissue	Unknown	Larvae
SC12	*S. frugiperda*	China:GuangXi:Nanning	N 22°48'02''	E 108°11'30''	2019/5/29	muscle tissue	Unknown	Larvae
SC13	*S. frugiperda*	China:GuangXi:Nanning	N 22°48'02''	E 108°11'30''	2019/5/30	muscle tissue	Unknown	Larvae
SC14	*S. frugiperda*	China:GuangXi:Nanning	N 22°48'02''	E 108°11'30''	2019/5/31	muscle tissue	Unknown	Larvae
SC15	*S. frugiperda*	China:GuangXi:Nanning	N 22°48'02''	E 108°11'30''	2019/6/1	muscle tissue	Unknown	Larvae
SC16	*S. frugiperda*	China:GuangXi:Nanning	N 22°48'02''	E 108°11'30''	2019/6/2	muscle tissue	Unknown	Larvae
SC17	*S. frugiperda*	China:GuangXi:Nanning	N 22°48'02''	E 108°11'30''	2019/6/3	muscle tissue	Unknown	Larvae
SC18	*S. frugiperda*	China:GuangXi:Nanning	N 22°48'02''	E 108°11'30''	2019/6/4	muscle tissue	Unknown	Larvae
SC19	*S. frugiperda*	China:GuangXi:Nanning	N 22°48'02''	E 108°11'30''	2019/6/5	muscle tissue	Unknown	Larvae
SC20	*S. frugiperda*	China:GuangXi:Nanning	N 22°48'02''	E 108°11'30''	2019/6/6	muscle tissue	Unknown	Larvae
SC21	*S. frugiperda*	China:GuangXi:Nanning	N 22°48'02''	E 108°11'30''	2019/6/7	muscle tissue	Unknown	Larvae
SC23	*S. frugiperda*	China:GuangXi:Nanning	N 22°48'02''	E 108°11'30''	2019/6/8	muscle tissue	Unknown	Larvae
SC24	*S. frugiperda*	China:GuangXi:Nanning	N 22°48'02''	E 108°11'30''	2019/6/9	muscle tissue	Unknown	Larvae
SC25	*S. frugiperda*	China:GuangXi:Nanning	N 22°48'02''	E 108°11'30''	2019/6/10	muscle tissue	Unknown	Larvae
SC26	*S. frugiperda*	China:GuangXi:Nanning	N 22°48'02''	E 108°11'30''	2019/6/11	muscle tissue	Unknown	Larvae

SC27	*S. frugiperda*	China:GuangXi:Nanning	N 22°48'02''	E 108°11'30''	2019/6/12	muscle tissue	Unknown	Larvae
SC28	*S. frugiperda*	China:GuangXi:Nanning	N 22°48'02''	E 108°11'30''	2019/6/13	muscle tissue	Unknown	Larvae
SC29	*S. frugiperda*	China:GuangXi:Nanning	N 22°48'02''	E 108°11'30''	2019/6/14	muscle tissue	Unknown	Larvae
SC30	*S. frugiperda*	China:GuangXi:Nanning	N 22°48'02''	E 108°11'30''	2019/6/15	muscle tissue	Unknown	Larvae
SC31	*S. frugiperda*	China:GuangXi:Nanning	N 22°48'02''	E 108°11'30''	2019/6/16	muscle tissue	Unknown	Larvae
SC32	*S. frugiperda*	China:GuangXi:Nanning	N 22°48'02''	E 108°11'30''	2019/6/17	muscle tissue	Unknown	Larvae
SC33	*S. frugiperda*	China:GuangXi:Nanning	N 22°48'02''	E 108°11'30''	2019/6/18	muscle tissue	Unknown	Larvae
SC34	*S. frugiperda*	China:GuangXi:Nanning	N 22°48'02''	E 108°11'30''	2019/6/19	muscle tissue	Unknown	Larvae
SC35	*S. frugiperda*	China:GuangXi:Nanning	N 22°48'02''	E 108°11'30''	2019/6/20	muscle tissue	Unknown	Larvae
SC36	*S. frugiperda*	China:GuangXi:Nanning	N 22°48'02''	E 108°11'30''	2019/6/21	muscle tissue	Unknown	Larvae
SC37	*S. frugiperda*	China:GuangXi:Nanning	N 22°48'02''	E 108°11'30''	2019/6/22	muscle tissue	Unknown	Larvae
SC38	*S. frugiperda*	China:GuangXi:Nanning	N 22°48'02''	E 108°11'30''	2019/6/23	muscle tissue	Unknown	Larvae
SC39	*S. frugiperda*	China:GuangXi:Nanning	N 22°48'02''	E 108°11'30''	2019/6/24	muscle tissue	Unknown	Larvae
SC40	*S. frugiperda*	China:GuangXi:Nanning	N 22°48'02''	E 108°11'30''	2019/6/25	muscle tissue	Unknown	Larvae
SC41	*S. frugiperda*	China:GuangXi:Nanning	N 22°48'02''	E 108°11'30''	2019/6/26	muscle tissue	Unknown	Larvae
SC42	*S. frugiperda*	China:GuangXi:Nanning	N 22°48'02''	E 108°11'30''	2019/6/27	muscle tissue	Unknown	Larvae
SC43	*S. frugiperda*	China:GuangXi:Nanning	N 22°48'02''	E 108°11'30''	2019/6/28	muscle tissue	Unknown	Larvae
SC44	*S. frugiperda*	China:GuangXi:Nanning	N 22°48'02''	E 108°11'30''	2019/6/29	muscle tissue	Unknown	Larvae
SC45	*S. frugiperda*	China:GuangXi:Nanning	N 22°48'02''	E 108°11'30''	2019/6/30	muscle tissue	Unknown	Larvae
SC46	*S. frugiperda*	China:GuangXi:Nanning	N 22°48'02''	E 108°11'30''	2019/7/1	muscle tissue	Unknown	Larvae

SC47	*S. frugiperda*	China:GuangXi:Nanning	N 22°48'02"	E 108°11'30"	2019/7/2	muscle tissue	Unknown	Larvae
SC48	*S. frugiperda*	China:GuangXi:Nanning	N 22°48'02"	E 108°11'30"	2019/7/3	muscle tissue	Unknown	Larvae
SC49	*S. frugiperda*	China:GuangXi:Nanning	N 22°48'02"	E 108°11'30"	2019/7/4	muscle tissue	Unknown	Larvae
SC50	*S. frugiperda*	China:GuangXi:Nanning	N 22°48'02"	E 108°11'30"	2019/7/5	muscle tissue	Unknown	Larvae
SC51	*S. frugiperda*	China:GuangXi:Nanning	N 22°48'02"	E 108°11'30"	2019/7/6	muscle tissue	Unknown	Larvae
SC52	*S. frugiperda*	China:GuangXi:Nanning	N 22°48'02"	E 108°11'30"	2019/7/7	muscle tissue	Unknown	Larvae
SC53	*S. frugiperda*	China:GuangXi:Nanning	N 22°48'02"	E 108°11'30"	2019/7/8	muscle tissue	Unknown	Larvae
SC54	*S. frugiperda*	China:GuangXi:Nanning	N 22°48'02"	E 108°11'30"	2019/7/9	muscle tissue	Unknown	Larvae
SC55	*S. frugiperda*	China:GuangXi:Nanning	N 22°48'02"	E 108°11'30"	2019/7/10	muscle tissue	Unknown	Larvae
SC56	*S. frugiperda*	China:GuangXi:Nanning	N 22°48'02"	E 108°11'30"	2019/7/11	muscle tissue	Unknown	Larvae
SC57	*S. frugiperda*	China:GuangXi:Nanning	N 22°48'02"	E 108°11'30"	2019/7/12	muscle tissue	Unknown	Larvae
SC58	*S. frugiperda*	China:GuangXi:Nanning	N 22°48'02"	E 108°11'30"	2019/7/13	muscle tissue	Unknown	Larvae
SC59	*S. frugiperda*	China:GuangXi:Nanning	N 22°48'02"	E 108°11'30"	2019/7/14	muscle tissue	Unknown	Larvae
SC60	*S. frugiperda*	China:GuangXi:Nanning	N 22°48'02"	E 108°11'30"	2019/7/15	muscle tissue	Unknown	Larvae
XS01	*S. frugiperda*	China:YunNan:Xishuangbanna	N 21°45'94"	E 101°56'46"	2019/7/7	whole body	Unknown	Adult
YA01	*S. frugiperda*	China:YunNan:Yaoan	N 25°50'42"	E 101°24'15"	2019/7/1	whole body	Unknown	Adult
YJ01	*S. frugiperda*	China:YunNan:Yuanjiang	N 23°59'66"	E 101°99'80"	2019/3/17	whole body	Unknown	Larvae
YJ02	*S. frugiperda*	China:YunNan:Yuanjiang	N 23°59'66"	E 101°99'80"	2019/3/17	whole body	Unknown	Larvae
YX01	*S. frugiperda*	China:YunNan:Yuxi	N 24°34'12"	E 102°54'25"	2019/5/16	whole body	Unknown	Larvae
YX02	*S. frugiperda*	China:YunNan:Yuxi	N 24°34'12"	E 102°54'25"	2019/5/16	whole body	Unknown	Larvae
YX03	*S. frugiperda*	China:YunNan:Yuxi	N 24°34'12"	E 102°54'25"	2019/5/16	whole body	Unknown	Larvae
YX04	*S. frugiperda*	China:YunNan:Yuxi	N 24°34'12"	E 102°54'25"	2019/5/16	whole body	Unknown	Larvae
YX05	*S. frugiperda*	China:YunNan:Yuxi	N 24°34'12"	E 102°54'25"	2019/5/16	whole body	Unknown	Larvae

YX06	*S. frugiperda*	China:YunNan:Yuxi	N 24°34'12"	E 102°54'25"	2019/5/16	whole body	Unknown	Larvae
YX07	*S. frugiperda*	China:YunNan:Yuxi	N 24°34'12"	E 102°54'25"	2019/5/16	whole body	Unknown	Larvae
YX09	*S. frugiperda*	China:YunNan:Yuxi	N 24°34'12"	E 102°54'25"	2019/5/16	whole body	Unknown	Larvae
YX10	*S. frugiperda*	China:YunNan:Yuxi	N 24°34'12"	E 102°54'25"	2019/5/16	whole body	Unknown	Larvae
YX11	*S. frugiperda*	China:YunNan:Yuxi	N 24°34'12"	E 102°54'25"	2019/5/16	whole body	Unknown	Larvae
ZT01	*S. frugiperda*	China:YunNan:Zhaotong	N 27°32'02"	E 103°70'59"	2019/8/5	whole body	Unknown	Larvae
SF01	*S. frugiperda*	China:YunNan:Yuanjiang	N 23°59'66"	E 101°99'80"	2019/3/17	whole body	Unknown	Larvae
SF02	*S. frugiperda*	China:YunNan:Yuanjiang	N 23°59'66"	E 101°99'80"	2019/3/17	whole body	Unknown	Larvae
SF03	*S. frugiperda*	China:YunNan:Yuanjiang	N 23°59'66"	E 101°99'80"	2019/3/17	whole body	Unknown	Larvae
SF04	*S. frugiperda*	China:YunNan:Yuanjiang	N 23°59'66"	E 101°99'80"	2019/3/17	whole body	Unknown	Larvae
SF05	*S. frugiperda*	China:YunNan:Yuanjiang	N 23°59'66"	E 101°99'80"	2019/3/17	whole body	Unknown	Larvae
SF06	*S. frugiperda*	China:YunNan:Yuanjiang	N 23°59'66"	E 101°99'80"	2019/3/17	whole body	Unknown	Larvae
SF07	*S. frugiperda*	China:YunNan:Yuanjiang	N 23°59'66"	E 101°99'80"	2019/3/17	whole body	Unknown	Larvae
SF08	*S. frugiperda*	China:YunNan:Yuanjiang	N 23°59'66"	E 101°99'80"	2019/3/17	whole body	Unknown	Larvae
SF09	*S. frugiperda*	China:YunNan:Yuanjiang	N 23°59'66"	E 101°99'80"	2019/3/17	whole body	Unknown	Larvae
SF10	*S. frugiperda*	China:YunNan:Yuanjiang	N 23°59'66"	E 101°99'80"	2019/3/17	whole body	Unknown	Larvae
SF11	*S. frugiperda*	China:YunNan:Yuanjiang	N 23°59'66"	E 101°99'80"	2019/3/17	whole body	Unknown	Larvae
SF12	*S. frugiperda*	China:YunNan:Yuanjiang	N 23°59'66"	E 101°99'80"	2019/3/17	whole body	Unknown	Larvae
SF13	*S. frugiperda*	China:YunNan:Yuanjiang	N 23°59'66"	E 101°99'80"	2019/3/17	whole body	Unknown	Larvae
SF14	*S. frugiperda*	China:YunNan:Yuanjiang	N 23°59'66"	E 101°99'80"	2019/3/17	whole body	Unknown	Larvae
SF15	*S. frugiperda*	China:YunNan:Yuanjiang	N 23°59'66"	E 101°99'80"	2019/3/17	whole body	Unknown	Larvae
SF16	*S. frugiperda*	China:YunNan:Yuanjiang	N 23°59'66"	E 101°99'80"	2019/3/17	whole body	Unknown	Larvae
SF17	*S. frugiperda*	China:YunNan:Yuanjiang	N 23°59'66"	E 101°99'80"	2019/3/17	whole body	Unknown	Larvae
SF18	*S. frugiperda*	China:YunNan:Yuanjiang	N 23°59'66"	E 101°99'80"	2019/3/17	whole body	Unknown	Larvae
SF25	*S. frugiperda*	China:YunNan:Yuanjiang	N 23°59'66"	E 101°99'80"	2019/3/17	whole body	Unknown	Larvae
SF26	*S. frugiperda*	China:YunNan:Yuanjiang	N 23°59'66"	E 101°99'80"	2019/3/17	whole body	Unknown	Larvae
SF27	*S. frugiperda*	China:YunNan:Yuanjiang	N 23°59'66"	E 101°99'80"	2019/3/17	whole body	Unknown	Larvae
SF31	*S. frugiperda*	China:YunNan:Yuanjiang	N 23°59'66"	E 101°99'80"	2019/3/17	whole body	Unknown	Larvae
SF32	*S. frugiperda*	China:YunNan:Yuanjiang	N 23°59'66"	E 101°99'80"	2019/3/17	whole body	Unknown	Larvae
SF33	*S. frugiperda*	China:YunNan:Yuanjiang	N 23°59'66"	E 101°99'80"	2019/3/17	whole body	Unknown	Larvae

SF37	*S. frugiperda*	China:YunNan:Yuanjiang	N 23°59'66''	E 101°99'80''	2019/3/17	whole body	Unknown	Larvae
SF38	*S. frugiperda*	China:YunNan:Yuanjiang	N 23°59'66''	E 101°99'80''	2019/3/17	whole body	Unknown	Larvae
SF39	*S. frugiperda*	China:YunNan:Yuanjiang	N 23°59'66''	E 101°99'80''	2019/3/17	whole body	Unknown	Larvae
SF40	*S. frugiperda*	China:YunNan:Yuanjiang	N 23°59'66''	E 101°99'80''	2019/3/17	whole body	Unknown	Larvae
SF41	*S. frugiperda*	China:YunNan:Yuanjiang	N 23°59'66''	E 101°99'80''	2019/3/17	whole body	Unknown	Larvae
SF42	*S. frugiperda*	China:YunNan:Yuanjiang	N 23°59'66''	E 101°99'80''	2019/3/17	whole body	Unknown	Larvae
SF43	*S. frugiperda*	China:YunNan:Yuanjiang	N 23°59'66''	E 101°99'80''	2019/3/17	whole body	Unknown	Larvae
SF44	*S. frugiperda*	China:YunNan:Yuanjiang	N 23°59'66''	E 101°99'80''	2019/3/17	whole body	Unknown	Larvae
SF45	*S. frugiperda*	China:YunNan:Yuanjiang	N 23°59'66''	E 101°99'80''	2019/3/17	whole body	Unknown	Larvae
SF46	*S. frugiperda*	China:YunNan:Yuanjiang	N 23°59'66''	E 101°99'80''	2019/3/17	whole body	Unknown	Larvae
SF47	*S. frugiperda*	China:YunNan:Yuanjiang	N 23°59'66''	E 101°99'80''	2019/3/17	whole body	Unknown	Larvae
SF48	*S. frugiperda*	China:YunNan:Yuanjiang	N 23°59'66''	E 101°99'80''	2019/3/17	whole body	Unknown	Larvae
SF49	*S. frugiperda*	China:YunNan:Yuanjiang	N 23°59'66''	E 101°99'80''	2019/3/17	whole body	Unknown	Larvae
SF50	*S. frugiperda*	China:YunNan:Yuanjiang	N 23°59'66''	E 101°99'80''	2019/3/17	whole body	Unknown	Larvae
SF51	*S. frugiperda*	China:YunNan:Yuanjiang	N 23°59'66''	E 101°99'80''	2019/3/17	whole body	Unknown	Larvae
SF52	*S. frugiperda*	China:YunNan:Yuanjiang	N 23°59'66''	E 101°99'80''	2019/3/17	whole body	Unknown	Larvae
SF53	*S. frugiperda*	China:YunNan:Yuanjiang	N 23°59'66''	E 101°99'80''	2019/3/17	whole body	Unknown	Larvae
SF54	*S. frugiperda*	China:YunNan:Yuanjiang	N 23°59'66''	E 101°99'80''	2019/3/17	whole body	Unknown	Larvae
SF55	*S. frugiperda*	China:YunNan:Yuanjiang	N 23°59'66''	E 101°99'80''	2019/3/17	whole body	Unknown	Larvae
SF56	*S. frugiperda*	China:YunNan:Yuanjiang	N 23°59'66''	E 101°99'80''	2019/3/17	whole body	Unknown	Larvae
SF57	*S. frugiperda*	China:YunNan:Yuanjiang	N 23°59'66''	E 101°99'80''	2019/3/17	whole body	Unknown	Larvae
SF58	*S. frugiperda*	China:YunNan:Yuanjiang	N 23°59'66''	E 101°99'80''	2019/3/17	whole body	Unknown	Larvae
SF59	*S. frugiperda*	China:YunNan:Yuanjiang	N 23°59'66''	E 101°99'80''	2019/3/17	whole body	Unknown	Larvae
SF60	*S. frugiperda*	China:YunNan:Yuanjiang	N 23°59'66''	E 101°99'80''	2019/3/17	whole body	Unknown	Larvae
SF61	*S. frugiperda*	China:YunNan:Yuanjiang	N 23°59'66''	E 101°99'80''	2019/3/17	whole body	Unknown	Larvae
SF62	*S. frugiperda*	China:YunNan:Yuanjiang	N 23°59'66''	E 101°99'80''	2019/3/17	whole body	Unknown	Larvae
SF63	*S. frugiperda*	China:YunNan:Yuanjiang	N 23°59'66''	E 101°99'80''	2019/3/17	whole body	Unknown	Larvae
SF64	*S. frugiperda*	China:YunNan:Yuanjiang	N 23°59'66''	E 101°99'80''	2019/3/17	whole body	Unknown	Larvae
SF65	*S. frugiperda*	China:YunNan:Yuanjiang	N 23°59'66''	E 101°99'80''	2019/3/17	whole body	Unknown	Larvae
SF66	*S. frugiperda*	China:YunNan:Yuanjiang	N 23°59'66''	E 101°99'80''	2019/3/17	whole body	Unknown	Larvae

SF67	*S. frugiperda*	China:YunNan:Yuanjiang	N 23°59'66''	E 101°99'80''	2019/3/17	whole body	Unknown	Larvae
SF68	*S. frugiperda*	China:YunNan:Yuanjiang	N 23°59'66''	E 101°99'80''	2019/3/17	whole body	Unknown	Larvae
SF69	*S. frugiperda*	China:YunNan:Yuanjiang	N 23°59'66''	E 101°99'80''	2019/3/17	whole body	Unknown	Larvae
SF70	*S. frugiperda*	China:YunNan:Yuanjiang	N 23°59'66''	E 101°99'80''	2019/3/17	whole body	Unknown	Larvae
SF71	*S. frugiperda*	China:YunNan:Yuanjiang	N 23°59'66''	E 101°99'80''	2019/3/17	whole body	Unknown	Larvae
SF72	*S. frugiperda*	China:YunNan:Yuanjiang	N 23°59'66''	E 101°99'80''	2019/3/17	whole body	Unknown	Larvae
SF73	*S. frugiperda*	China:YunNan:Yuanjiang	N 23°59'66''	E 101°99'80''	2019/3/17	whole body	Unknown	Larvae
SF74	*S. frugiperda*	China:YunNan:Yuanjiang	N 23°59'66''	E 101°99'80''	2019/3/17	whole body	Unknown	Larvae
SF75	*S. frugiperda*	China:YunNan:Yuanjiang	N 23°59'66''	E 101°99'80''	2019/3/17	whole body	Unknown	Larvae
SF76	*S. frugiperda*	China:YunNan:Yuanjiang	N 23°59'66''	E 101°99'80''	2019/3/17	whole body	Unknown	Larvae
SF77	*S. frugiperda*	China:YunNan:Yuanjiang	N 23°59'66''	E 101°99'80''	2019/3/17	whole body	Unknown	Larvae
SF78	*S. frugiperda*	China:YunNan:Yuanjiang	N 23°59'66''	E 101°99'80''	2019/3/17	whole body	Unknown	Larvae
SF82	*S. frugiperda*	China:YunNan:Yuanjiang	N 23°59'66''	E 101°99'80''	2019/3/17	whole body	Unknown	Larvae
SF83	*S. frugiperda*	China:YunNan:Yuanjiang	N 23°59'66''	E 101°99'80''	2019/3/17	whole body	Unknown	Larvae
SF84	*S. frugiperda*	China:YunNan:Yuanjiang	N 23°59'66''	E 101°99'80''	2019/3/17	whole body	Unknown	Larvae
SF85	*S. frugiperda*	China:YunNan:Yuanjiang	N 23°59'66''	E 101°99'80''	2019/3/17	whole body	Unknown	Larvae
SF86	*S. frugiperda*	China:YunNan:Yuanjiang	N 23°59'66''	E 101°99'80''	2019/3/17	whole body	Unknown	Larvae
SF87	*S. frugiperda*	China:YunNan:Yuanjiang	N 23°59'66''	E 101°99'80''	2019/3/17	whole body	Unknown	Larvae

Table S21 | Databases of comparative genomics analysis contains *Spodoptera frugiperda* and other seven species including *Bombyx mori*, *Danaus plexippus*, *Drosophila melanogaster*, *Helicoverpa armigera*, *Manduca sexta*, *Plutella xylostella* and *Spodoptera litura*.

Species	Version	Wingspan(mm)
Bombyx mori	PRJDA20217	40-50
Danaus plexippus	PRJNA72423	86-124
Drosophila melanogaster	PRJNA13812	5-10
Helicoverpa armigera	PRJNA378437	27-38
Manduca sexta	PRJNA81037	100-120
Plutella xylostella	PRJNA78271	12-16
Spodoptera frugiperda	Our Study	37-42
Spodoptera litura	PRJNA416314	32-40
